# Pan‐3D Genome Analysis Reveals the Roles of Structural Variation in Chicken Chromatin Architectures, Domestication and Production Traits

**DOI:** 10.1002/advs.202520068

**Published:** 2026-02-15

**Authors:** Zhen Zhou, Danfeng Cai, Changbin Zhao, Siyu Zhang, Jiahao Li, Zhaofeng Zhang, Shaofen Kong, Xin Yang, Xiaoli Zhou, Farhad Bordbar, Fayi Chen, Yaohuan Xu, Zhe Zhang, Lihong Gu, Zhenhui Li, Xiquan Zhang, Wen Luo, Jingting Shu, Bolin Cai, Qinghua Nie

**Affiliations:** ^1^ State Key Laboratory of Swine and Poultry Breeding Industry Guangdong Laboratory for Lingnan Modern Agriculture Guangdong Provincial Key Lab of Agro‐Animal Genomics and Molecular Breeding Key Laboratory of Chicken Genetics, Breeding and Reproduction Ministry of Agriculture and Rural Affairs National‐Local Joint Engineering Research Center for Livestock Breeding College of Animal Science South China Agricultural University Guangzhou China; ^2^ Key Laboratory for Poultry Genetics and Breeding of Jiangsu Province Jiangsu Institute of Poultry Science Yangzhou China; ^3^ Institute of Animal Science & Veterinary Medicine Hainan Academy of Agricultural Sciences Haikou China; ^4^ Wuhan Generead Biotechnology Co., Ltd Wuhan China; ^5^ Huazhi Biotechnology Co., Ltd Changsha China

**Keywords:** Gallus gallus, graph‐based pan‐genome, loop interaction, pan‐3D genome, structure variations

## Abstract

As the first sequenced non‐mammalian amniote, the chicken (*Gallus gallus*) has served as a major source of cost‐effective and protein‐enriched foods since domestication. However, how structural variations (SVs) affect 3D genome reorganization to influence domestication and production traits remains unclear in chickens. Here, fifteen *de novo* chromosome‐level genome assemblies are newly generated, along with high‐throughput chromosome conformation capture (Hi‐C), ATAC and RNA sequencing data. By integrating 13 published assemblies, the first pan‐3D genome resource is constructed, spanning genes, SVs, and chromatin architectures, to investigate the dynamic characteristics of the 3D genome at different levels and the roles of SVs in the conservation and reorganization of chromatin architectures. Furthermore, candidate SVs and their linked genes are identified for domestication and production traits based on 1,735 resequencing accessions. Notably, the 240‐bp and 81‐bp SVs in the *TSHR* and *DIO2* genes are considered the key targets in artificial selection for seasonal reproduction, and a 266‐bp deletion upstream of the *KLF3* gene affects carcass performance by rewriting the chromatin loop interaction network. Finally, SVs significantly improve the predictive accuracy in genomic selection models. Collectively, this study presents a comprehensive pan‐3D resource to advance functional genomic research and breeding practice for the community.

## Graphical abstract

This study firstly presents a comprehensive and high‐resolution pan‐3D genome resource in chicken. Our findings reveal the role of structural variations in 3D genome architectures, and how they influence the domestication process and production traits at the 3D genome level. Our study provides a valuable reference for advancing functional genomics and breeding practice.

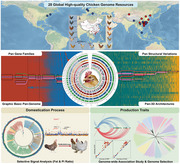

## 1 Introduction

Chicken (*Gallus gallus*) is an important poultry species, providing a cost‐effective source of animal protein [1], while also serving as a key model for research in evolution and developmental biology [2, 3]. Domestic chicken was initially domesticated from the wild red jungle fowl (RJF) approximately 9500 ± 3300 years ago [4]. Following domestication, chickens acquired a highly diverse genetic background via interbreeding with local wild fowl species [4], and the diversity was further enhanced by continuous crossbreeding among different breeds and international trade [5]. Currently, chicken is one of the most widely distributed poultry in the world, exhibiting high genetic diversity, particularly in Asian and African populations [6]. Since the first draft genome [7] was sequenced in 2004, to the completion of the first telomere‐to‐telomere (T2T) genome assembly [8] in 2023, genomic research in chicken has been consistently developing. However, the reference genome derived from a specific breed limits the identification of genetic diversity across different breeds, especially in structural variations (SVs) [9].

The development of long‐read sequencing technologies has facilitated the application of pan‐genome research from the microbial field to the animal and plant fields [10, 11]. Pan‐genome, especially graph‐based pan‐genome, provides a crucial platform for investigating the role of SVs in evolution and the regulation of complex traits [12, 13, 14, 15]. Current research on chicken pan‐genome has advanced from resequencing to the haplotype, even T2T‐based, which has laid solid foundation and frameworks for understanding genomic diversity, domestication process and genetic basis of production traits [16, 17, 18, 19, 20, 21]. Extensive research has demonstrated that SVs are closely associated with the rearrangement of chromatin architectures, which in turn regulates gene expression and impacts the phenotype [22, 23]. For example, investigators have demonstrated that SVs may affect key genes associated with high‐altitude adaptation through disrupting the stability of TAD structures [24, 25]. Furthermore, recent studies have conducted pan‐3D genome analysis to reveal how genetic variations identified from the pan‐genome influenced the reorganization of 3D chromatin architectures, including compartments, topologically associating domains (TADs), and chromatin loops [26, 27]. High‐resolution 3D genome maps can provide solid support for revealing the complex gene regulatory mechanisms in livestock and poultry [28, 29]. The advantage of the pan‐genome in depicting and interpreting the 3D chromatin architectures has been further highlighted [30]. Therefore, it is necessary to construct a pan‐3D genome to comprehensively investigate the role of SVs in the reorganization of chromatin architectures and their contribution to domestication and production traits in chicken.

In this study, we constructed the first chicken pan‐3D genome resource by integrating 28 global high‐quality genomes, 1,735 short‐read resequencing accessions, 15 high‐throughput chromatin conformation capture (Hi‐C) sequencing, along with ATAC‐seq and RNA‐seq. To begin with, we newly generated 15 high‐quality *de novo* genome assemblies of Chinese indigenous chicken based on Pacific Biosciences high‐fidelity (HiFi) long‐read and high‐resolution Hi‐C data. In this way, we observed the distinct divergence of the conserved landscape of gene families and SVs in the chicken pan genome. Then, we systematically investigated the dynamic characteristics in chromatin 3D architectures, particularly the roles of SV in reorganizing TADs and chromatin loops. Combined with population genetic analysis and Capture Hi‐C sequencing, we highlighted the key SVs linked to domestication and production traits, and further screened the candidate SV genes and SV‐loop genes. Finally, we demonstrated that SVs significantly improved the predictive accuracy in genomic selection (GS), offering a practical strategy for accelerating molecular marker‐assisted breeding.

## 2 Results

### 2.1 High‐Quality Assemblies and Annotation of Fifteen Chicken Genomes

**FIGURE 1:**
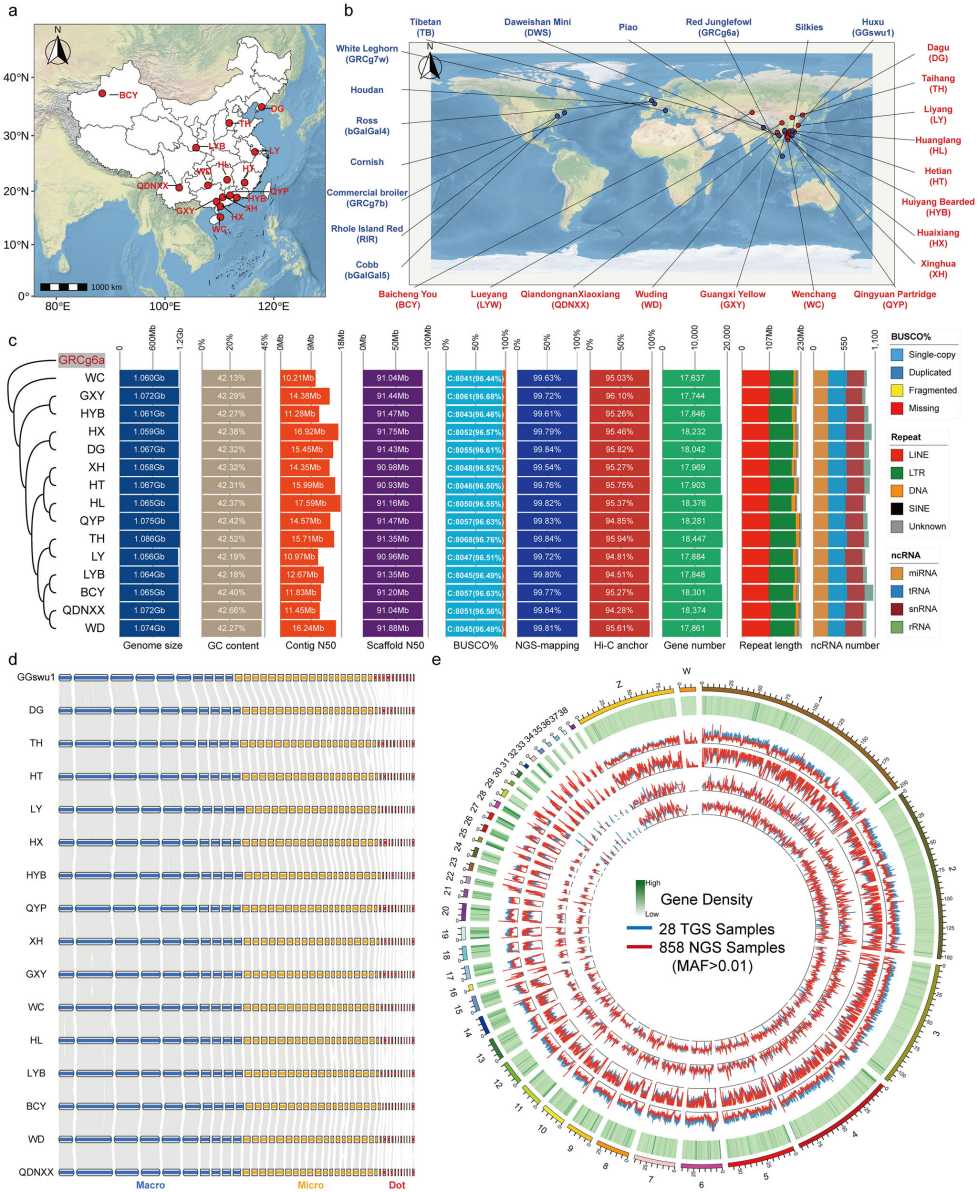
*De novo* assembly and annotation of fifteen chicken genomes. (a) Sampling locations of fifteen newly assembled genomes. (b) Geographic distribution of newly assembled (red) and published (blue) accessions. (c) Statistics of genome assembly, annotation, completeness, and contiguity assessment of fifteen newly assembled accessions. (d) Whole‐genome collinearity analysis between the newly assembled genomes and the T2T genome. (e) Distribution of SNP variations and genetic features based on 28 assemblies (blue line) and 858 global accessions (red line). The circles plot, from outer to inner, represents gene density, SNP density, nucleotide diversity (Pi), dN, and dS.

China is one of the main domestication centers for domestic chicken, characterized by extremely high genetic diversity [4, 31]. Utilizing HiFi long‐read (≈30× coverage) and Hi‐C (≈300× coverage) sequencing data (Tables S2–S4), we de novo generated 15 genomes from indigenous breeds spanning major geographic regions across China (Figure 1a). After Hi‐C‐assisted assembly, the contig N50 sizes ranged from 10.22 to 17.60 Mb, scaffold N50 from 90.94 to 91.88 Mb, and the final assembled genome sizes from 1.056 to 1.086 Gb (Table S12a). Remapping rate of their respective short‐read data yielded alignment rates exceeding 99.5% (Table S12b). Additionally, the completeness score of Benchmarking Universal Single‐Copy Orthologs (BUSCO) ranged from 96.4% to 96.8% (Table S12c). Consistent with the T2T genome (GGswu1) [8], a total of 10 macro chromosomes, 19 micro chromosomes, and 10 dot chromosomes were successfully assembled for each accession (Figure 1d). We annotated around 920–1,065 non‐coding RNA (miRNA, tRNA, rRNA, and snRNA) and 17,637–18,447 protein‐coding genes for each accession (Table S13a,d). Repetitive sequences made up around 19.15% of each accession, with long interspersed nuclear elements (LINE, 9.54%) and long terminal repeats (LTR, 8.47%) being the most abundant (Table S13c). The annotated gene sets showed high functional completeness, as supported by BUSCO scores of 94.57%–95.15% (Table S13b). The high completeness and contiguity achieved in these newly assembled genomes (Figure 1c) provide a robust foundation for subsequent pan‐genome and pan‐3D genome studies.

### 2.2 Pan‐Gene Family Characteristic of 28 Global Chicken Genomes

To comprehensively capture the genomic diversity of chicken, we constructed the pan‐genome by integrating 13 published high‐quality genomes [8, 17, 18, 32, 33] with our 15 newly assembled genomes (Figure 1b). This collection included 1 T2T genome (GGswu1), 1 ancestral breed (GRCg6a), 2 commercial layer‐type breeds (GRCg7w, RIR), 4 commercial meat‐type breeds (GRCg7b, bGalGal4, bGalGal5, Cornish), as well as 20 indigenous breeds (Houdan, DWS, Piao, Silkies, Tibetan, DG, TH, LY, HL, HT, HYB, HX, XH, QYP, WD, WC, GXY, QDNXX, LYW, BCY) (detailed in Table S1). The high representativeness of our pan genome was supported by the similar pattern (Figure 1e) and significant correlation (*p* < 0.001) (Figure S1) with a global dataset of *Gallus gallus* individuals [4]. The population genetic patterns were closely matched, including SNP density, nucleotide diversity (Pi), dN and dS. Using these 28 genome assemblies, we investigated gene family evolution in chicken and found that contracted gene families varied substantially (range: 334–7,608) while expanded gene families exhibited much less variation (range: 109–333) (Figure S2 and Table S14) These patterns might reflect adaptive expansions in sensory and immune functions, accompanied by structural simplification through gene family contractions (Figure S3).

**FIGURE 2:**
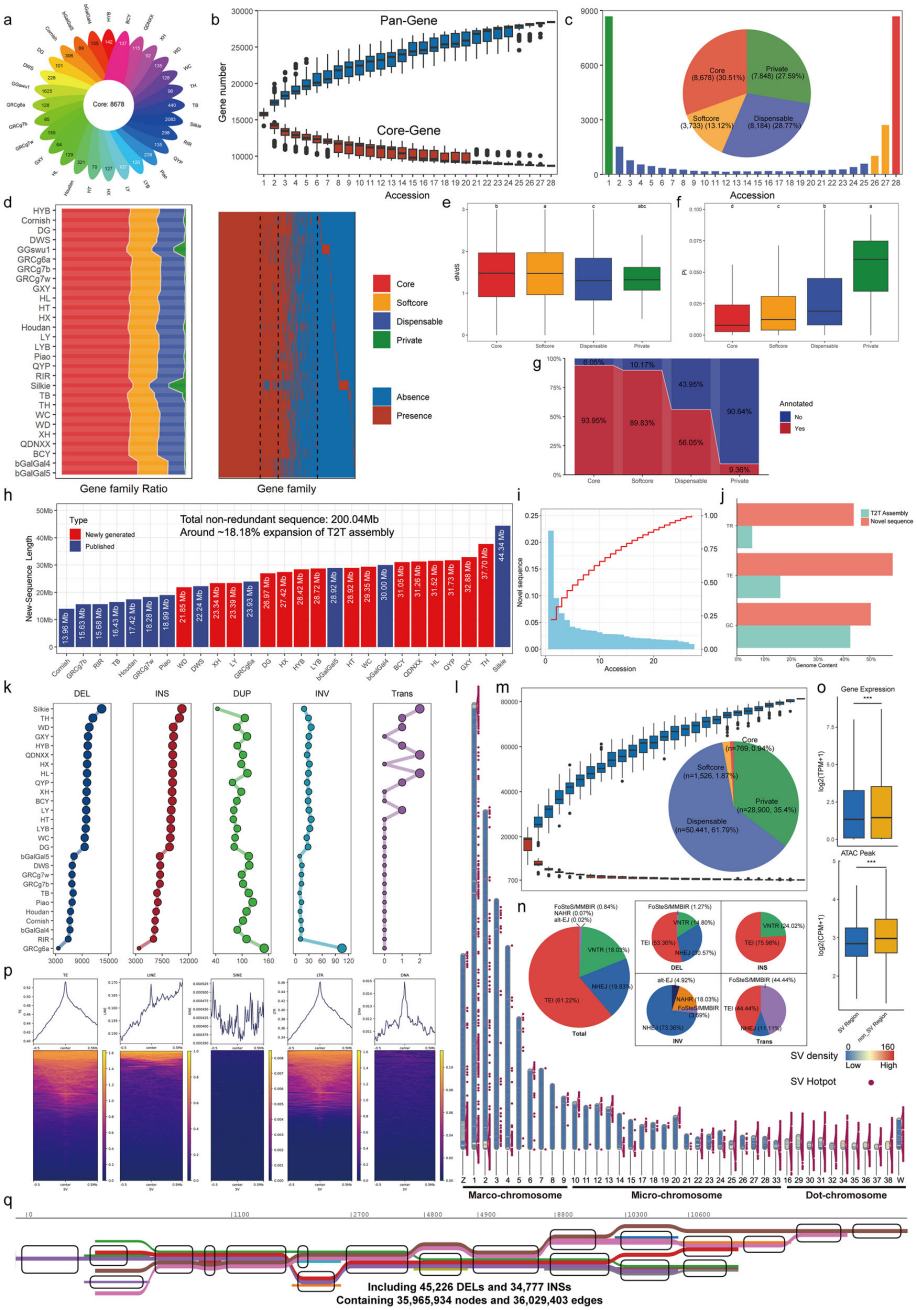
Pan genome pattern of gene families and structural variations (SVs) in chicken. (a) Statistics of conserved and lineage‐specific gene families. (b) The pan‐gene family model of chicken. (c) Composition of the pan‐gene family at different conserved levels. (d) Proportion of pan‐gene families and presence‐absence information for each genome. Comparison of (e) dN/dS value, (f) nucleotide diversity (Pi) and (g) InterPro domain annotation ratio among pan‐gene families. (h) Length of novel sequence detected from each assembly compared with the T2T genome. (i) Distribution and cumulative curve of observed frequencies of novel sequences. (j) TR, TE, and GC contents of the T2T genome and novel sequences. (k) Number of SVs detected in each assembly. (l) Distribution of SV hotspots in the genome. Red points represent SV hotspots. Heatmaps represent SV density in a 100‐kb window. (m) The pan‐SV model and proportion of pan‐SV in chicken. n) Proportion of driving formation mechanisms identified in the chicken's SV. (o) Comparison of gene expression and ATAC signal between the SV region and the non‐SV region. (p) Enrichment patterns of TE within the upstream and downstream 500‐kb region of SV. (q) Local visualization of chicken graph‐based pan‐genome. Significant differences in e) and f) were assessed by one‐way ANOVA followed by Tukey's HSD test for multiple comparisons. Groups not sharing the same letter were significantly different, *p* < 0.05. Significant differences in o) were assessed by a two‐sided independent *t*‐test; ^***^ *p* < 0.001. Abbreviation: TEI: transposable element insertion; NHEJ: nonhomologous end joining; VNTR: variable number of tandem repeats; FoSTeS/MMBIR: fork stalling and template switching/microhomology‐mediated break‐induced repair; NAHR: nonallelic homologous recombination; alt‐EJ: alternative end joining.

Pan genome analysis further clustered all genes into 28,443 pan‐gene families (Figure 2a; Table S15). As the number of genomes added, the pan‐set was increased and nearly approached saturation, while the core‐set decreased and then stabilized, confirming the representativeness of our pan‐genome pool (Figure 2b). The pan genome was composed of ∼30.51% core families (present in all genomes), ≈13.12% softcore families (present in more than 90% genomes), ≈27.59% private families (present in a specific genome) and ≈28.77% dispensable families (all others) (Figure 2c). However, as for individual genomes, highly conserved families (core and softcore) averaged more than three‐quarters, with private families constituting less than 2% (Figure 2d), which highlighted the crucial roles of the pan‐genome for capturing the complete extent of genetic diversity. Furthermore, highly conserved families exhibited elevated dN/dS ratios coupled with reduced Pi value, indicating a long‐term positive selection followed by recent selective sweeps in chicken (Figure 2e–f). Functionally, ≈90% genes in highly conserved families contained InterPro domains, compared to only ≈9% in private families (Figure 2g). Core families were enriched in essential cellular functions, while pan families might contribute to lineage‐specific adaptation through innovations in immune responses (Figure S4a,b).

### 2.3 Novel Sequence Identification and Structural Variation Landscape in Pan Genome

One of the advantages of the pan genome is the ability to capture the sequence not present in a single linear reference genome [34, 35]. Based on our pan‐genome resource, a total of 200.04 Mb of novel sequence was captured compared to the T2T genome, with 13.96–44.34 Mb for each accession (Figure 2h). It exceeded most published chicken pan‐genomes [16, 17, 18, 19] and was comparable to a recent haplotype‐resolved pan‐genome [20]. The detection rate of novel sequences was 2.73% in each assembly (Figure 2i). Consistent with recent studies [17, 19], the novel sequence exhibited significantly higher proportions of tandem repeat (TR, 43.94%) and transposable element (TE, 58.59%) compared to the normal sequence (TR: 5.74%; TE: 16.32%), which might contribute to novel sequence accumulation (Figure 2j). Interestingly, genes in novel sequence were significantly enriched in skeletal muscle‐related terms, likely reflecting their roles in production traits under strong selection in modern breeding (Figure S5). To further screen the high‐confidence set of genetic variants in chicken, we identified 20,529,969 SNPs, 4,672,013 InDels and 81,636 SVs based on our pan‐genome resource (Figure S6a and Table S28). As a kind of large‐scale genomic variation, SV regions were enriched with SNPs and InDels (Figure S6b), suggesting the key roles of SVs in genomic innovation. Among SVs, we detected five types of SVs, including deletions (DEL), insertions (INS), inversions (INV), duplications (DUP), and translocations (Trans) (Figure 2k; Figure S6c). Most of them were in the intergenic region (56.8%), followed by introns (27.3%) (Figure S6d). These SVs were primarily small‐scale (< 2 kb); only INVs were mainly large‐scale (>20 kb) (Figure S6e). We randomly selected 30 SVs for each genome and verified them in Integrative Genomics Viewer using their long‐read sequencing data. The results showed that most of them (> 93%) were confirmed to be present (Figure S6f). We also identified SV hotspots and found that most SV hotspots were enriched in regions close to the telomeres of chromosomes (Figure 2l), indicating these regions were susceptible to accumulating genetic variation. Furthermore, SVs were probably associated with reduced transcriptional activity and chromatin accessibility (Figure 2o).

SVs in chicken exhibited remarkably high lineage‐specific diversity, with highly conserved types constituting less than 3% of all pan‐SV dataset (Table S16). Consequently, unlike the pan‐gene families, the pan‐SV accumulation curve didn't saturate (Figure 2m), and the increase in SVs across chicken genomes was primarily driven by poorly conserved SVs (dispensable and private) (Figure S7a,b). Notably, private‐type SVs became the predominant source of SVs as the size increased (Figure S7c). Functionally, core SVs were mainly associated with the fundamental biological processes, while dispensable SVs were enriched in the adaptive functional pathways, including olfactory signal transduction, dopaminergic synaptic regulation, and circadian rhythm entrainment (Figure S4c,d). Furthermore, a total of 81.99% of SVs were assigned to a specific formation mechanism, with transposable element insertion (TEI) being the predominant driver (61.22%), followed by non‐homologous end joining (NHEJ, 19.83%) and variation in tandem repeat number (VNTR, 18.03%) (Figure 2n; Table S16), which was consistent with previous studies [20, 36]. Detailed Mechanisms varied by SV subtypes and their characteristics. NHEJ dominated INVs but was absent in INSs (Figure 2n) Besides, the contribution of NHEJ was increased with SV size, while TEI was linked to the formation of poorly conserved SVs (Figure S7d,e). Importantly, most of SV regions and breakpoints were significantly enriched for TEs, particularly LTR elements, emphasizing their important role in genomic innovation of chicken (Figure 2p; Figure S7f). To capture and represent this complex landscape of structural variation at population levels, we built a graph‐based pan‐genome, including 35,965,934 nodes and 36,029,403 edges (Figure 2q).

### 2.4 Construction and Characterization of High‐Resolution Pan‐3D Genome Atlas

**FIGURE 3:**
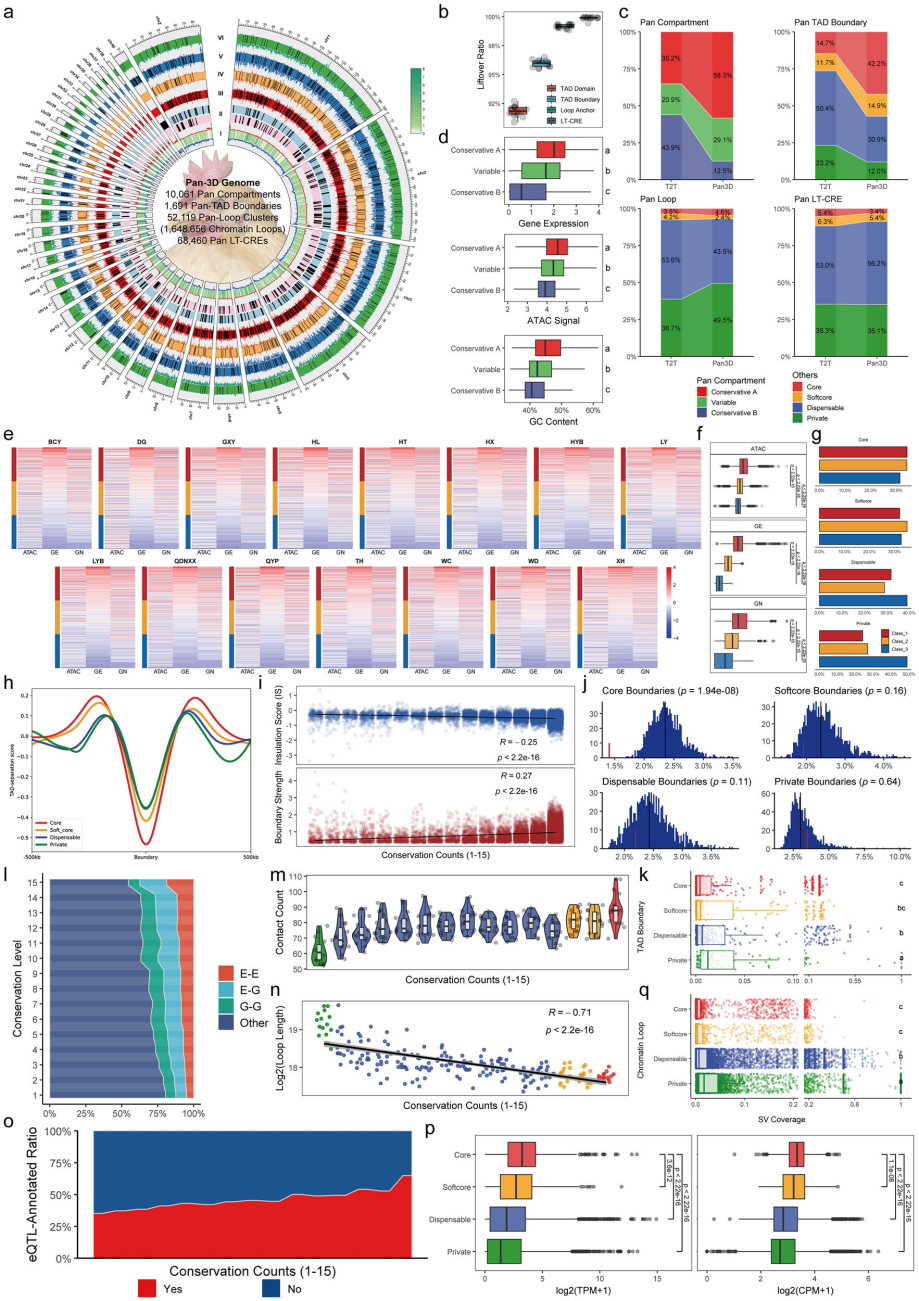
Dynamic landscape of chicken pan‐3D genome. (a) Circos plot showing the landscape of 3D chromatin architectures in the chicken pan‐3D genome atlas. I: gene (heatmap), TE (lower blue line) and SV (upper red line) density. II: Alternative TAD domains (lower pink tile: fusion events, upper blue tile: neo events). III‐VI: Distribution of pan TAD boundary types (core‐type: red, softcore‐type: orange, dispensable‐type: blue, private‐type: green), loop anchor density (upper line) and LT‐CREs density (lower line). (b) LiftOver rates of 3D chromatin architectures from 15 newly assembled genomes to the T2T genome. (c) Proportion of pan types in chromatin architectures between the pan‐3D genome and the T2T genome. (d) Comparisons of gene expression, ATAC signal and GC content among pan compartments. (e) Classification of TAD boundaries into active, neutral and inactive types based on the ATAC signal (ATAC), gene expression (GE), and gene number (GN). (f) Comparison of three‐type TAD boundaries in ATAC, GE and GN. (g) Proportion of three‐type TAD boundaries in different pan‐TAD boundaries. (h) Enrichment pattern of isolation scores upstream and downstream 500‐kb region of pan‐TAD boundaries. (i) Correlation analysis of insulation score and boundary strength across different conservation levels. (j) Observed (red bar) and expected distribution (blue histograms) of SV coverage in different pan‐TAD boundaries. (k) Comparisons of SV coverage among different pan‐TAD boundaries. (l) Proportion of LT‐CRE and gene annotation of loops with different conserved levels. E and G represent LT‐CRE and the gene, respectively. (m) Distribution of the contact count of loops with different conserved levels. (n) Correlation analysis between the span of loop length and conserved levels. (o) Proportion of eQTL annotation in loops with different conserved levels. (p) Comparison of gene expression and ATAC signal between pan loops. (q) Comparisons of SV coverage among different pan loops. Significant differences in d) k) and q) were assessed by one‐way ANOVA followed by Tukey's HSD test for multiple comparisons. Groups not sharing the same letter were significantly different, *p* < 0.05. Significant differences in f), j) and p) were assessed by a two‐sided Wilcoxon rank‐sum test. Correlations in i) and n) were assessed by Pearson's correlation analysis.

Understanding how genomic variation shapes the 3D genome is a fundamental challenge in genome research [22, 23]. To address this approach, we integrated Hi‐C, ATAC‐seq, and RNA‐seq of 15 chicken genomes to generate a high‐resolution 3D genome architecture atlas from compartments to loops in chicken (Figures S8 and S9; Tables S4–S8). Compared to using a single T2T reference genome, aligning the Hi‐C data to their assembled genomes could improve the yield of valid pair reads (Figure S10a). Using the newly assembled genomes, we identified a total of 13,909 A and 15,756 B compartments, 20,785 TAD boundaries, 21,217 TAD domains, 3,349,274 chromatin loops, and 348,287 long‐range cis‐regulatory elements (LT‐CREs) in chicken. Compartment and TAD showed little difference from those in a single reference. In contrast, a marked increase in the number of loops and LT‐CREs was detected, providing an abundant resource to capture the complex regulatory networks between genes and genetic variation (Figure S10c–e). Using these 3D resources, we built the first comprehensive pan‐3D genome atlas for chicken (Figure 3a). Our pan‐3D genome successfully identified 10,061 of 10,780 (93.33%) single‐copy orthologous pairs in genome regions to categorize conservative A/B and variable compartments (Table S17). For TAD and loop levels, over 90% chromatin architectures and elements were also successfully incorporated to construct our pan‐3D genome (Figure 3b). Compared to the single reference, the pan‐3D genome showed obvious divergence in conserved 3D chromatin architectures (Figure 3c; Tables S17–S24). It likely indicates that our pan‐3D genome could correct for systematic artifacts from single‐reference read mapping while also avoiding boundary distortions in compartments, TADs, and loop anchors caused by large‐scale SVs. Around 30% compartments were variable across different accessions and showed intermediate gene expression level, ATAC signal and GC content compared to the conservative A/B compartment (Figure 3d).

TAD is defined as one of the fundamental structural and functional units in 3D genome, and the conservation of their boundaries is tightly linked to gene regulatory patterns and epigenetic landscapes [37]. In chicken, over half of TAD boundaries were highly conserved, while only 12% were private across 15 genomes (Figure 3c). Besides, we categorized all pan‐TAD boundaries into three activity classes according to the ATAC signal strength, gene expression and gene number (Figure 3e,f). We found that class 3 (relatively inactive) was predominant in private boundaries, indicating lower chromatin accessibility and transcriptional activity in species‐specific boundaries (Figure 3g). Highly conserved TAD boundaries exhibited stronger insulation, in contrast to the weaker insulation observed inside their flanking domains (Figure 3h), supporting the crucial role of conserved boundaries in maintaining stable gene expression. Overall, the conservation degree of TAD boundaries was positively correlated with boundary strength degree (Figure 3i), which is consistent with previous study [26]. The high boundary strength likely demonstrates strong selective constraints against genetic variation, and previous study showed that SVs are more likely to occur in the interiors of TAD rather than boundaries due to the selection pressure [38]. Consistently, we observed that SVs were depleted at almost all pan‐TAD boundaries except private type, with the strongest depletion in core type (Figure 3j), which suggested strong selection pressure on boundary regions of chicken. This signal was less pronounced in private boundaries, potentially due to their more recent origin with insufficient time for purifying selection (Figure 3j,k). Similarly, we investigated the SV pattern in the TAD reorganization events, including conserved, fusion and neo‐TADs (Figure S11a, Table S25). Although most domains were conserved, some fusion and neo events were also observed across different genomes (Figure S11b). Correspondingly, genes and ATAC peaks involved in these domains were prone to be differentially expressed and accessible compared to those in conserved domains (Figure S11c). However, SVs were significantly absent in all three types of domains, indicating the limited roles of SVs in driving domain reorganization in chicken (Figure S11d).

Our pan‐3D genome provided a greatly expanded dataset of chromatin loops, which is defined as key elements for translating genetic variation into regulatory impact in the genome [39, 40]. Moreover, we combined loop anchors, ATAC peaks, and distance to transcription start site (TSS) to identify LT‐CREs for loop annotation, including LT‐CREs‐LT‐CREs (E‐E), genes‐genes (G‐G) and LT‐CREs‐genes (E‐G) types (Figure S10b). While a similar annotation profile of loops was observed between the pan‐3D genome and a single reference, the total number of loops was greater in our pan‐3D resource, providing a more comprehensive loop interaction network (Figure S10e,f). Over 40% of ATAC‐seq peaks were identified as LT‐CREs, highlighting that long‐range chromatin interactions were widespread in the chicken genome (Figure S10g). To investigate the relationship between loop conservation and functional feature, we clustered all 1,648,656 non‐redundant chromatin loops into a total of 52,119 pan‐loop clusters, including 4.6% core, 2.4% softcore, 43.5%dispensable and 49.5% private types (Table S22). According to LT‐CREs, they both exhibited relatively low conservation degree (Core < 5%) compared to other chromatin architectures (Figure 3c). Interestingly, we found that as the conservation degree decreased, the span of loops was significantly increased, while we observed marked reductions in LT‐CRE/gene annotation rate, interaction counts and eQTL [41] annotation rate (Figure 3l‐o). Correspondingly, core loops exhibited significantly higher gene expression and ATAC signal strength than other‐type loops (Figure 3p). Highly conserved loops were also significantly depleted in SVs compared to poorly conserved loops (Figure 3q). Functional enrichments showed that more extensive terms were significantly enriched in core loops than other‐type loops, particularly in muscle development‐related terms, suggesting their important roles in production traits in broiler (Figure S12). Together, although fewer proportion of highly conserved loops in our pan‐3D genome, they served as the crucial and conserved regulatory functional hub in chicken.

### 2.5 Selection on the TSHR‐DIO2 Axis During Chicken Domestication

**FIGURE 4:**
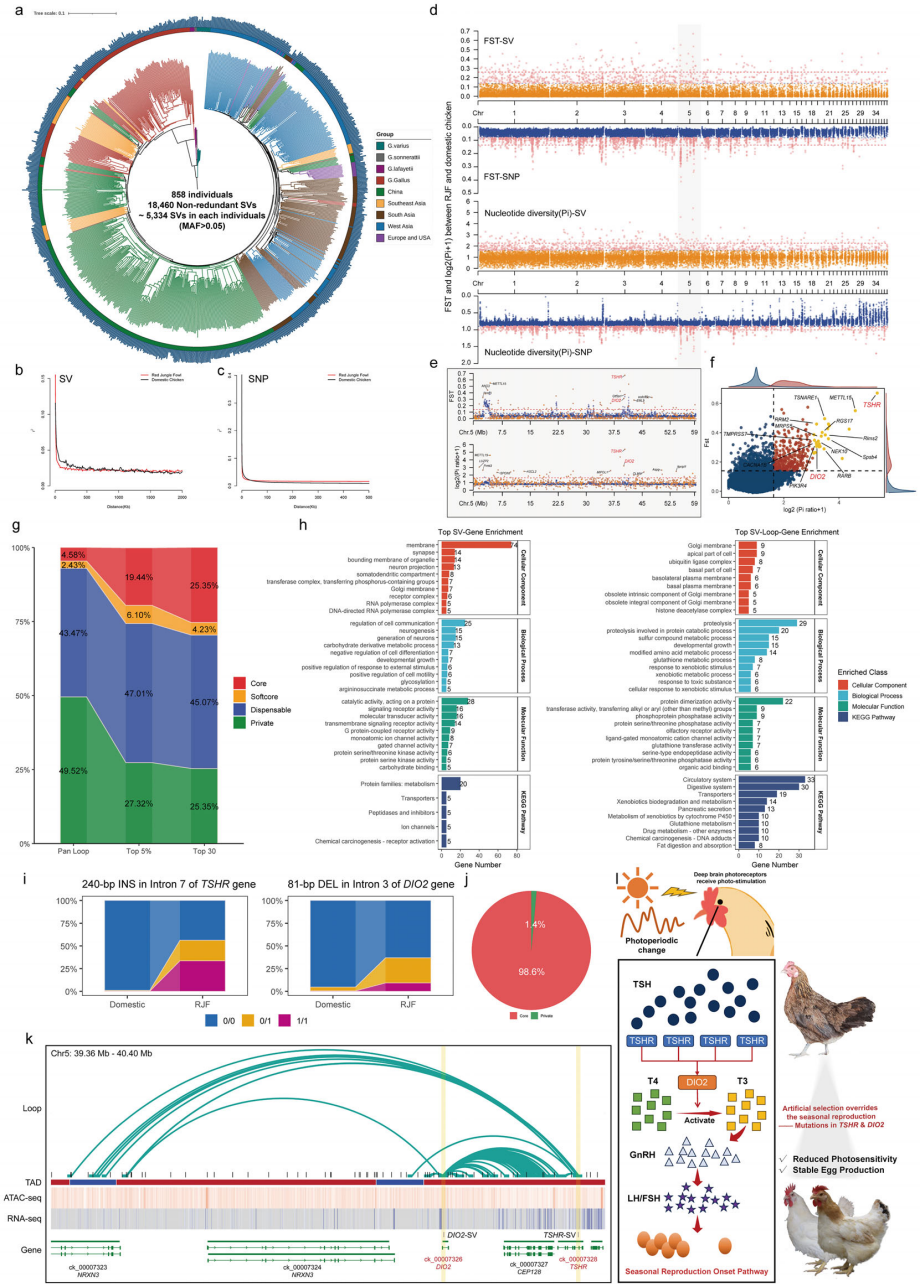
TSHR‐DIO2 axis is a critical regulatory axis in chicken domestication. (a) Phylogenetic tree of 858 global individuals based on SVs. The blue bars outside the tree referred to the SV number detected for each accession. LD decay of (b) SVs and (c) SNP between RJF and domestic chicken. (d) The selection signal (Fst and Pi‐ratio) of RJF vs. domestic chicken based on SNPs and SVs. The horizontal dashed lines correspond to the top 5% threshold (blue) and 1% threshold (red). The orange and blue points represented SVs and SNPs, respectively. (e) Zoom in view of selection signals on chromosome 5. (f) Integrating Fst and Pi‐ratio to screen candidate SVs related to domestication. Dashed lines represent top 5% thresholds (Fst: horizontal; Pi‐ratio: vertical). Highlighted points: top 30 candidate SVs ranked by Euclidean distance. (g) Proportion of pan‐type loops across the top 30, top 5% candidate SV regions and the genome‐wide degree. (h) Functional enrichment of candidate SV genes and SV‐loop genes. All displayed terms correspond to the top 10 ranked by adjusted *p* value. (i) Genotype distribution of candidate SVs in the *TSHR* and *DIO2* genes between RJF and domestic chicken. (j) Proportion of pan‐type of candidate SV‐related loops in *TSHR* and *DIO2* genes. (k) Integrative genomics viewer (IGV) of loops network related to candidate SVs in *TSHR* and *DIO2* genes. (l) Schematic diagram of photoperiod to seasonal reproduction via the TSHR‐DIO2 axis and the signatures of artificial selection during domestication.

As described above, SVs exhibit more distinct lineage specificity compared to gene families, suggesting their potential role in driving divergence during domestication and artificial selection [42, 43]. By aligning resequencing accession to our graph‐based pan‐genome (Table S26), a total of 70,090 SVs were identified across 858 global *Gallus gallus* individuals (Table S28). After filtering (MAF > 0.05), we obtained a final set of 18,460 SVs (ranging from 1,422 to 9,000 per individual) for subsequent selection signal analysis (Figure 4a; Table S29). Compared to SNPs, SVs showed faster LD decay in RJF than that in domestic chicken, indicating stronger purifying selection on SV during domestication (Figure 4b,c). Selective signal analysis further revealed that SVs exhibited more distinct genetic differentiation (Fst) and selective sweep signatures (Pi‐ratio) between RJF and domestication (Figure 4d; Tables S32 and S33). Notably, the signals on chromosome 5 were particularly strong in the regions spanning 2.29–4.22 Mb and 39.09–45.78 Mb, including several candidate genes such as protein arginine methyltransferase 3 (*Prmt3*), methyltransferase 15 (*METTL15*), thyroid stimulating hormone receptor (*TSHR*), and deiodinase iodothyronine type II (*DIO2*) genes, which were linked to methylation procession and photoperiodic regulation (Figure 4e). By integrating the top 5% of Fst and Pi‐ratio signals, we identified 167 candidate SVs and 180 SV genes associated with domestication, and the *TSHR* and *DIO2* were also among the top 30 candidate SV genes ranked by Euclidean distance (Figure 4f). To further explore their regulatory functions, we linked the 167 candidate SVs to 1,154 genes via 468 pan‐loop clusters, defining these as SV‐loop genes (Table S36). Interestingly, candidate SVs were strongly enriched in core‐type chromatin loops compared to the genome‐wide level (Figure 4g), which suggests these SVs occurring in loops potentially affected key functional units in the genome. Enrichment analysis revealed the different functional profiles between SV genes and SV‐loop genes, extending our understanding of SV‐mediated gene regulation mechanisms (Figure 4h). A classic example was that the 240‐bp INS in the *TSHR* gene and an 81‐bp DEL in the *DIO2* gene (Figure 4f), particularly the 240‐bp INS in *TSHR*, was only detected in the GRCg6a (genome of RJF) in our pan‐SV database (Figure S13). These two genes were both under strong selective pressure in domestic chickens compared to RJF (Figure 4i). Besides, the loops containing these two SVs were almost core‐type (Figure 4j). Specifically, these loops mediated frequent interactions between *TSHR* and *DIO2* genes, as well as interaction with the nearby *CEP128* gene and the distal *NRXN3* gene located in another TAD (Figure 4k). The TSHR‐DIO2 axis has been proven to serve as a central photoperiodic regulator, particularly in regulating seasonal reproduction and environmental adaptation [44, 45, 46, 47]. Collectively, our pan genome and pan‐3D genome further confirmed this axis to be a central selective target during domestication, thus regulating seasonal reproduction and enabling stable egg production (Figure 4l).

### 2.6 SV Affected Carcass Traits by Regulating Distal Genes Through Chromatin Loops

Beyond analyzing the domestication process, the pan genome also significantly enhanced the power to detect genetic variation affecting production traits [48]. By aligning 877 resequencing F2 accessions to our graph‐based pan‐genome (Table S27), we detected a total of 68,985 SVs and yielded a final set of 23,645 SVs (MAF > 0.05) for subsequent analysis (Figure S14a; Tables S28 and S30). The high concordance between SNP and SV‐based population genetic analyses validated the genetic robustness of selected F2 population (Figure S14b‐i). Analysis of the linkage between SVs and SNPs within ± 500 kb revealed that over half (51%) of SVs showed low‐to‐moderate linkage (R^2^ < 0.7) with nearby SNPs, suggesting the presence of hidden genetic variation not detectable through SNPs alone (Figure S15a). Using both SV and SNP markers, we performed the genome‐wide association study (GWAS) to identify the variations associated with 49 production traits (Table S31). Multiple SV‐based signals were detected in genomic regions where SNP‐based signals were absent, supporting that SVs could capture genetic information inaccessible to SNP‐based analyses (Figure S16–S24; Tables S34 and S35). A total of 241 SVs and 327 SV genes were significantly associated with production traits, especially carcass and growth performance traits (Figure S15b,d). We validated several candidate SVs to confirm their presence and genotype in other independent populations (Figure S25). We also identified SV‐loop genes for candidate SVs in SV‐GWAS, and a total of 673 non‐redundant pan‐loop clusters and 2,061 SV‐loop genes were screened (Figure S15d;; Table S37). Like the pattern observed in Section 2.5, these loops also showed a greatly higher proportion of core loops than the genome‐wide degree (Figure S15c).

**FIGURE 5:**
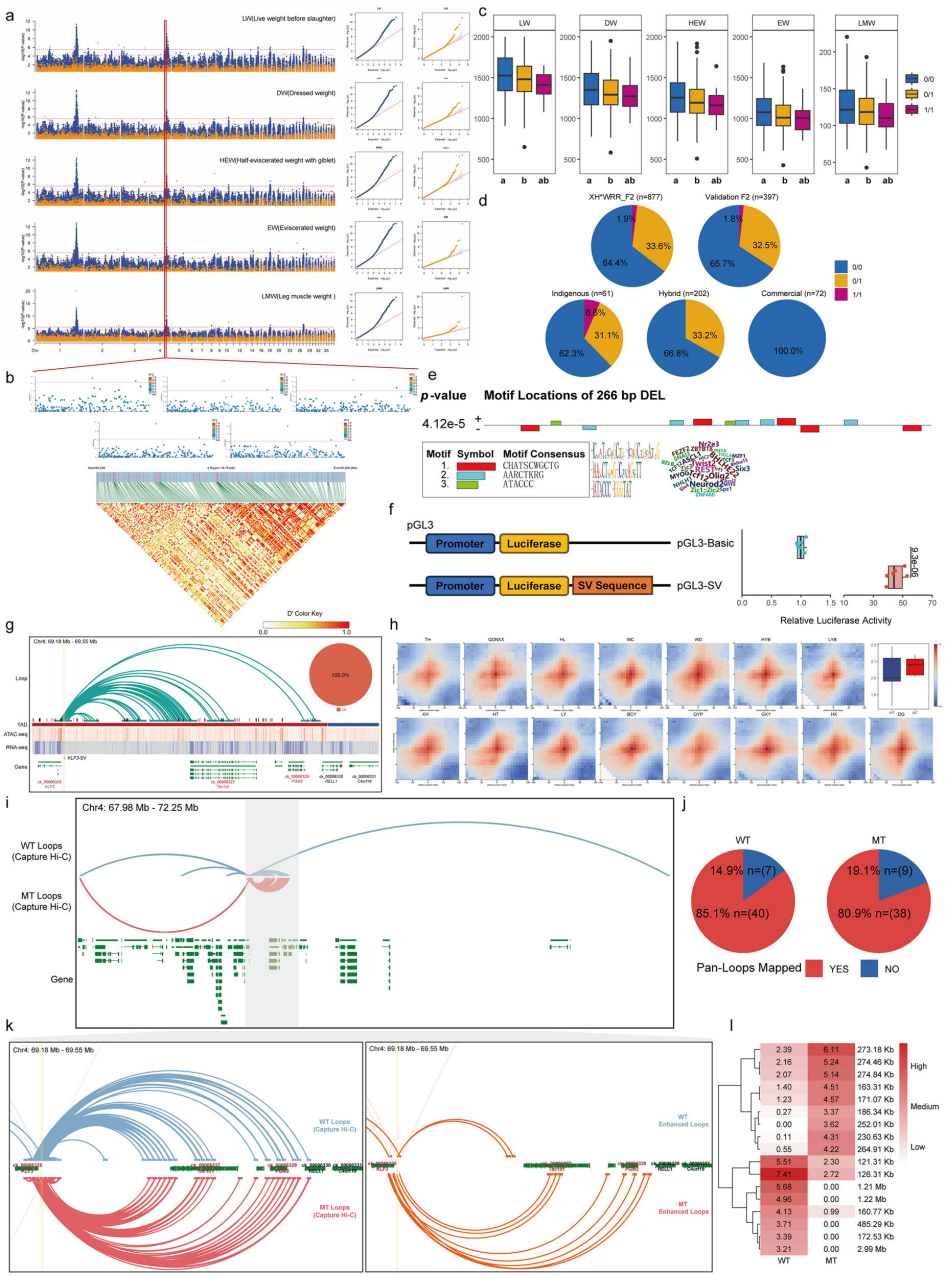
An upstream SV of *KLF3* affects carcass performance traits by regulating distal genes through chromatin loops. (a) Manhattan and quantile‐quantile plots of SNP‐based and SV‐based GWAS for five carcass performance traits. The blue and orange points represent SNP and SV markers. Upper and lower horizontal dashed lines were the significance threshold of SNPs and SVs (*p* = 1/N, where N referred to the number of markers remaining after LD pruning) (b) Linkage between candidate SV and other variations within 10 kb upstream and downstream. (c) Comparisons of five carcass performance traits among different SV genotypes. (d) Genotype distribution of candidate SV in XH*WRR F2, other validation F2, commercial, hybrid, and indigenous broiler populations. (e) Transcription factor (TF) motif prediction in candidate SV sequence. (f) A dual‐luciferase reporter assay evaluated the activity of the candidate SV sequence in chicken primary myoblasts. (g) IGV visualization of loop networks related to the candidate SV upstream of *KLF3* gene. The pie plot represents the proportion of pan‐type of these SV‐related loops. h) Pileup analysis of candidate SV‐related loops in each accession. The boxplot represents the loop intensity between wild and mutant accessions. (i) Capture Hi‐C analysis identifies the loop interactions landscape in candidate SV regions between wild (WT) and mutant‐type (MT) muscle. (j) Mapping rate of loops between Capture Hi‐C and pan‐3D genome. (k) Zoom in view of the mainly regulatory region of candidate SV and enhanced loops between WT and MT groups. (l) Heatmap of interaction scores of significantly enhanced loops between WT and MT groups. Significant differences in c) were assessed by one‐way ANOVA followed by Tukey's HSD test for multiple comparisons. Groups not sharing the same letter were significantly different, *p* < 0.05. Significant differences in f) were assessed by a two‐sided independent *t*‐test.

Notably, a 266‐bp DEL upstream of Kruppel‐like factor 3 (*KLF3*) was considered a potential marker to affect carcass performance, including live weight before slaughter, dress weight, half‐eviscerated weight with giblet, eviscerated weight, and leg muscle weight (Figure 5a). It showed extremely low linkage with nearby SNPs (Figure 5b), indicating that this information couldn't be captured in SNP‐based GWAS. In the analyzed F2 population, this SV occurred primarily in heterozygotes, showing a negative impact on carcass performance. This genotype distribution was also validated in another independent F2 population (Figure 5c,d). Moreover, this SV was completely absent in commercial broiler (relatively high carcass performance), presented as heterozygotes in hybrid broilers (intermediate carcass performance), and showed the highest genotype frequency in indigenous broiler (relatively low carcass performance), supporting its negative impact on chicken carcass performance (Figure 5d). Mechanistically, the SV sequence enriched several muscle development‐related transcription factors, including myogenin (MYOG) and myogenic factor 5 (MYF5) [49, 50], and this sequence showed significantly strong enhancer activity as validated by the dual‐luciferase reporter assay (Figure 5e‐f). It should be noted that this SV was located on an anchor for 47 completely conserved loops, with a span of 40–280 kb, which overlapped distal genes upstream of *KLF3*, including TBC1 domain family member 1 (*Tbc1d1*) and phosphoglucomutase 2 (*PGM2*) (Figure 5g). *KLF3*, *Tbc1d1*, and *PGM2* play important roles in metabolic modulation, especially insulin signaling and glucose metabolism [51, 52]. Furthermore, previous studies have identified them as the candidate genes affecting production traits in chicken [53, 54, 55]. In our study, we found that the SV could significantly down‐regulate the expression of SV‐nearby gene *KLF3* and distal genes *Tbc1d1* and *PGM2* (Figure S26a). Moreover, these three genes exhibited highly similar expression patterns across different development stages of broiler and during myoblast proliferation and differentiation (Figure S26b,c). Contrary to expectation, the interaction strength of these loops was enhanced in the mutant‐type (MT) group comparted to wild‐type (WT) in our pan‐3D genome (Figure 5h). Considering the potential artifacts introduced by genomes from diverse genetic backgrounds, we performed Capture Hi‐C in individuals with the same genetic background to validate the SV impact of reorganizing the loop interaction network (Figure S27a–c; Table S9‐S11). The Capture Hi‐C results validated the candidate loops identified in our pan‐3D genome, confirming the present and highly conserved degree in this region (Figure 5i,j). Beyond the pen‐3D genome results, Capture Hi‐C revealed additional long‐range interaction (3.9 Mb for WT, 1.2 Mb for MT) in this region (Figure 5i; Figure S27c). We noticed that the MT exhibited significantly weakened long‐range interactions compared to WT, while the relatively short‐range interactions with *Tbc1d1* and *PGM2* were significantly enhanced (Figure 5k,l; Table S38). The rewriting of the loop interaction network might represent a compensatory effect for the functional loss induced by the SV. Collectively, these results demonstrated that the 266‐bp deletion upstream of *KLF3* affected carcass performance traits by rewriting the loop interaction network.

### 2.7 Structural Variation Improved Predictive Accuracy of Genomic Selection in Chicken

**FIGURE 6:**
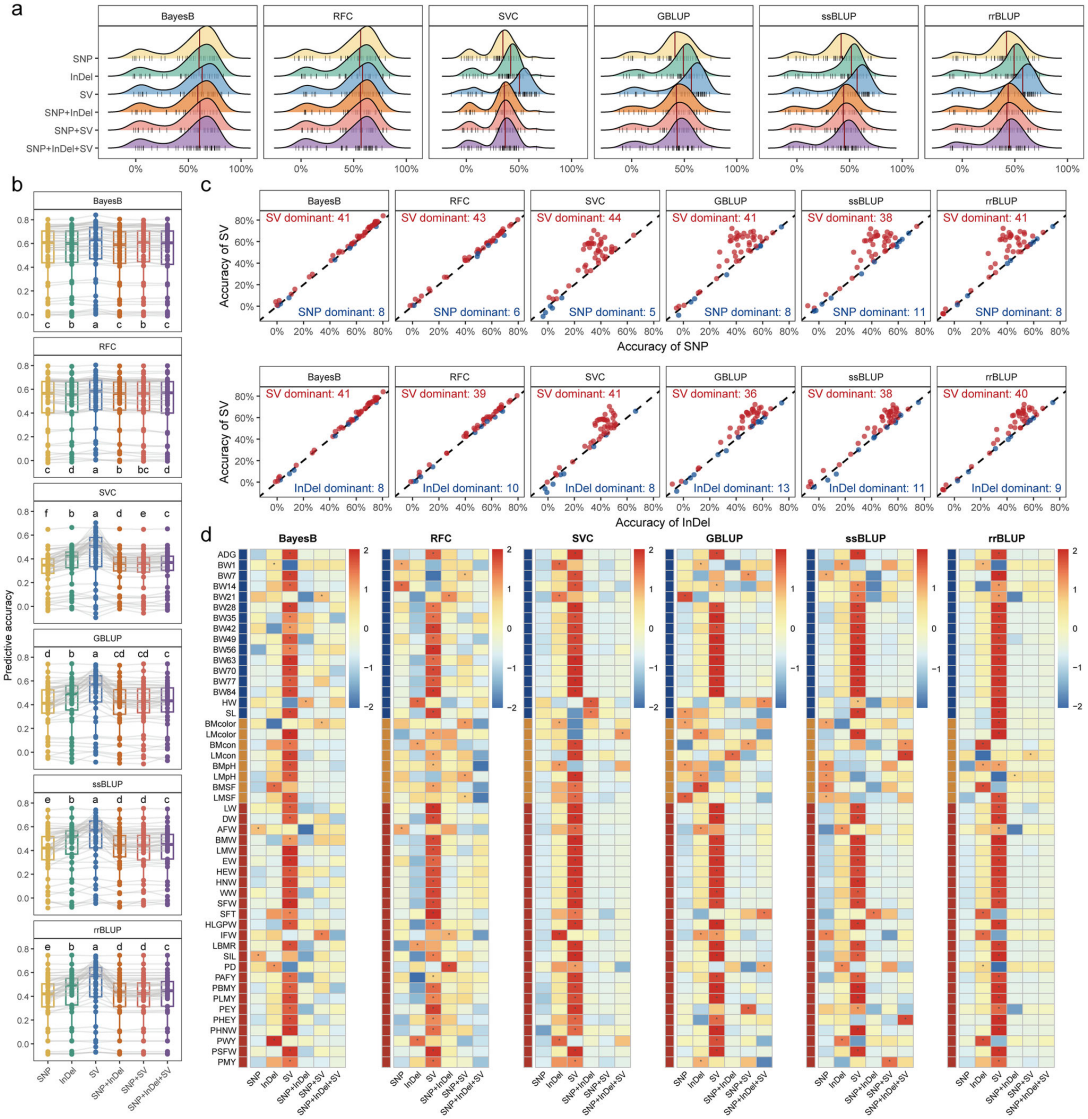
Prediction accuracy of genomic selection (GS) models using different genetic variations. (a) Distribution of predictive accuracy of different GS models using different combinations of genetic variants. The vertical red lines represent the median of predictive accuracy of 49 production traits. Individual data points are displayed as vertical lines at the bottom of each ridge plot. (b) Comparison of GS accuracy using different types of genetic variants. (c) Comparison of GS accuracy using SNPs, InDels, and SVs as markers. The red points represent the traits where SV markers yielded higher predictive accuracy than SNP/InDel markers. The blue points represent the opposite case. The numbers indicate the count of traits where each type of marker dominated. (d) Heatmaps of predictive accuracy for each production trait. Heatmap color represents the row‐scaled values of predictive accuracy. The asterisks in the heatmap indicate the combination of genetic variants with the highest predictive accuracy in this trait. Significant differences in b) were assessed by a paired sample t‐test. Groups not sharing the same letter were significantly different (*p* < 0.05). Abbreviation: BayesB: Bayesian Regression Model B; RCF: Random Cluster Forests; SVC: Support Vector Classification; GBLUP, ssBLUP, and rrBLUP: Genomic/Single‐step/ Ridge Regression Best Linear Unbiased Prediction. The full names of 49 production traits were listed in Table S39.

SVs could significantly improve the predictive accuracy of GS models, laying a solid foundation for the application of MAS in breeding practice [56, 57]. Therefore, we further utilized different genetic marker combinations to construct 6 GS models in 49 production traits, including Bayesian regression model B (BayesB), random cluster forests (RCF), support vector classification (SVC), genomic, single‐step, and ridge regression best linear unbiased prediction (GBLUP, ssBLUP and rrBLUP) models (Table S39). We found that utilizing different combinations of genetic markers influenced the predictive accuracy of GS models for production traits. Particularly in SVC, GBLUP, ssBLUP, and rrBLUP, the predictive accuracy based on SVs was significantly superior to that of other marker combinations (Figure 6a,b). Although SVs could enhance predictive accuracy, their incorporation into models based on SNPs or SNP‐InDel combinations didn't yield significant improvements, suggesting that SNP‐based signals may dominate or mask SV effects in these models (Figure 6a,b). Across most traits, SV‐based predictions were superior to those using only SNPs or InDels, further highlighting the importance of using SVs in GS model construction (Figure 6c). Improvements were most pronounced in carcass and growth performance traits, with more limited effects on the meat‐quality performance trait, which were consistent with the results of SV‐GWAS (Figure 6d; Figure S15b). As mentioned in the literature review, meat‐quality performance is one kind of complex trait with various biochemical properties, which are largely determined by metabolites serving as flavor precursors [58, 59, 60]. A recent study has developed a valuable roadmap to associate flavor‐related metabolites with genetic variations through metabolome‐based GWAS (mGWAS) [61]. Future studies with more focus on flavor‐related metabolites utilized as molecular phenotypes in SV‐based mGWAS or GS are therefore suggested. Overall, our analysis demonstrated that incorporating SVs into GS could substantially enhance the predictive accuracy of GS models, providing a valuable tool for SV‐based MAS application in chicken breeding practice.

## 3 Discussion

Pan‐genome has been demonstrated to characterize evolutionary features, improve completeness of the genome, and capture genetic markers linked to complex production traits [16, 17, 18, 19, 20]. Recently, the pioneering T2T‐based pan‐genome has provided a nearly complete pan‐genome resource and a fundamental framework for analyzing the contribution of SVs in complex traits genetics [21], which also conceptually inspired our general pan‐genome analysis. However, it remains unclear how SVs reorganize the 3D genome to affect domestication and production traits in chicken. To address these challenges, the core innovation of our present study was designed to generate a comprehensive and high‐resolution pan‐3D genome resource in chicken. The genetic representativeness of our pan‐3D genome was comparable to or even exceeding those of currently available pan‐genome resources in chicken [16, 17, 18, 19, 20]. Importantly, we firstly extend pan‐genome analysis into 3D genome level to study how genetic variations, especially SVs, affect domestication and production traits through chromatin architectures.

Seminal studies in plant pan‐3D genomes have demonstrated that SVs are closely associated with the reorganization of chromatin architectures, particularly at the TAD and loop levels [26, 27]. However, one unexpected finding in our work is that we didn't find a significantly SV‐enriched pattern in both pan‐TAD boundaries and TAD domain reorganization events in chicken. In contrast, the depletion pattern of SVs was observed at almost all pan‐TAD boundaries and TAD domains reorganization events, indicating strong negative selection pressure and functional constraints on SVs at TAD levels. As a kind of relatively large‐scale genetic variation, SV exhibited highly lineage‐specific diversity in chicken. Although it may play a positive role in evolutionary adaptation and phenotypic diversity [25], its negative impact on the genome is often more significant [62]. TADs are a kind of crucial functional units in the 3D genome, maintaining genomic stability by restricting the chromatin interactions [63]. and the unexpected TAD changes will potentially lead to developmental disorders and diseases [64, 65]. The enrichment of SVs in plants may be attributed to the absence of CTCF, which results in the formation of only TAD‐like structures that are more plastic and susceptible to reorganization compared to animals [66, 67].

Unlike TADs, we observed that the proportion of core‐type loops overlapping SVs linked to domestication and production traits was markedly higher than the global genomic average. Our findings suggest that SVs could exert long‐range regulatory effects on the genome rather than only influencing nearby genes. An important example is that two SVs in *TSHR* and *DIO2* genes showed a strong selective signal between RJF and domestic chicken. The loops overlapping with these two SVs exhibited frequent interactions between *TSHR* and *DIO2* genes, providing novel 3D‐genome insights to understand the roles of the TSHR‐DIO2 axis in chicken domestication. Additionally, another example is that a 266‐bp DEL upstream of the *KLF3* genes regulate distal genes *Tbc1d1* and *PGM2* through highly conserved loops to affect carcass performance. Generally, SVs were defined as the unstable factors serving as sites of the regulatory network and genomic fragility [68]. However, the loop interaction strength observed in this region was higher in MT compared to WT. Capture Hi‐C validation further revealed that the DEL overlapped not only relatively proximal interactions in this candidate region, but also long‐range regulatory interactions in extremely distal regions. The presence of this DEL led to the complete loss of long‐range interactions, but enhanced the proximal interaction of *Tbc1d1* and *PGM2* genes. Hence, a possible explanation might be that the DEL sequence with strong activity regulated the candidate genes via these conserved proximal loops. The DEL reduced their transcriptional activity, which in turn triggered the rewriting of loop interaction to compensate by suppressing long‐range contacts and enhancing these conserved proximal ones. This finding challenges the view of SVs as merely destabilizing factors. Benefiting from the comprehensive pan‐3D genome resource, we were able to systematically investigate the complexity of the 3D regulatory network, particularly enhancing our understanding of SV function in rewriting chromatin loop interactions.

Several limitations in the present study could be addressed in future research. First, our pan‐3D genome resource, including pan SV, graph‐based pan‐genome, and pan‐3D architectures, relied on the first chicken T2T genome [8] as a reference backbone. A future T2T‐based pan‐genome resource will be essential to reveal a more detailed regulatory network of the chicken genome. Second, considering the role of repetitive sequences in driving genomic innovation, further research will be necessary to construct a comprehensive pan‐repetitive sequence atlas. Third, given the genetic heterogeneity across different accessions, the findings about the SV‐3D relationship should be interpreted with caution, and further validate the causality by targeted CRISPR editing followed by 3D genome assessment.

## 4 Conclusion

This study provided the first pan‐3D genome resource in chicken and systematically characterized its landscape across gene families, SVs, chromatin compartments, TADs, and loops. We further assessed the dynamic features and SVs' contribution to chromatin architectures reorganization. Leveraging these resources, multiple crucial SVs and their regulatory networks were identified for domestication and production traits, which also enhanced the accuracy of genomic selection. Our work provides an important resource and new insights for the research community.

## 5 Experimental Section

### 5.1 Samples Collection for Genome Assembly

Fifteen Chinese indigenous chicken breeds were sampled in their original habitats (Table S1). For each breed, 8‐week‐old (inflection point of chicken growth curve) [69] females were selected, and tissue samples were collected for sequencing. An equal quantity of heart, liver, spleen, breast muscle, and leg muscle was combined to give the mixed RNA sample for RNA‐seq for annotation. Breast muscle or liver was subjected to DNA extraction for constructing short‐read and long‐read sequencing libraries (Table S2). For pan‐3D genome construction, Hi‐C, ATAC‐seq, and RNA‐seq libraries were generated from the same breast muscle tissue of each female accession (Tables S3–S8).

### 5.2 Samples for Population Genetic Analysis

To analyze the role of SV in chicken domestication, we employed the global individuals (*n* = 858) from published research [4], including Red jungle fowl (RJF) with all five subspecies, the other three types of jungle fowls, and domestic chickens at home and abroad. To analyze the role of SV in production traits, we generated ≈30× short‐read sequencing data for XH×WRR F2 individuals (*n* = 877) using the DEBSEQ‐T7 platform (BGI, Shenzhen, China). The F2 population was made up of a reciprocal cross between Xinghua broiler (a slow‐growing broiler) and White Recessive Rock broiler (a fast‐growing broiler) in our previous work [70, 71].

### 5.3 Samples for SV Validation

To validate SV genotyping and its effect in chromatin loop rewriting, we extracted DNA from four chicken populations, which were distinct from those breeds used in our pan‐3D genome analysis. These included a commercial population (*n* = 72, bred by Cobb and Aviagen broilers), an indigenous population (*n* = 61, bred by Chinese “882” yellow broilers), a hybrid population (*n* = 202, bred by commercial and indigenous broilers) from Foshan Nanhai Poultry Breeding Co., Ltd (Guangdong, China), and an F2 population (*n* = 397, made up of a reciprocal cross between partridge‐feather broiler and yellow‐feather broiler) from KwangFeng Industrial Co., Ltd (Guangdong, China).

### 5.4 Short and Long‐Read Library Construction

High‐quality genomic DNA samples from 15 accessions' breast muscle or liver were extracted following the standard CTAB (Cetyltrimethylammonium Bromide) method. For short‐read sequencing, the libraries were prepared using the Nextera DNA Flex Library Prep Kit (Illumina, San Diego, CA, USA), and the Illumina NovaSeq 6000 platform (Illumina, San Diego, CA, USA) was employed to perform short‐read sequencing. For high‐fidelity (HiFi) long‐read sequencing, SMRTbell target size libraries were constructed using 15‐kb preparation solutions according to PacBio's standard protocol with SMRTbell prep kits (Pacific Biosciences, CA, USA). The SMRTbell libraries were purified using AMPure PB Beads (Pacific Biosciences, CA, USA). HiFi (CCS) sequencing was performed on PacBio Sequel II with Sequencing Primer V5 and Sequel II Binding Kit 2.2.

### 5.5 Library Construction of Hi‐C

The breast muscles of the same accessions in genomic assembly were subjected to construct Hi‐C sequencing. Two replicates were performed for each accession. The sample was cross‐linked with formaldehyde and quenched with glycine. The cell nuclei were extracted, resuspended, and purified to digest DNA with 150U MboI enzyme. The digested DNA was marked by biotin‐14‐dCTP. Ligated DNA was obtained by T4 DNA ligase and purified by QIAamp DNA Mini Kit (Qiagen, Hilden, Germany). Purified DNA was sheared to ≈400 bp fragments, and the point ligation junctions were pulled down by Dynabeads MyOne Streptavidin C1 (ThermoFisher, MA, USA). The Hi‐C library was sequenced on the Illumina HiSeq X Ten platform (San Diego, CA, USA). The Hi‐C sequencing data with an average sequencing depth of ≈300× per sample were obtained. Fastp (v0.23.2) [72] was used to perform quality filtering, and the clean data were treated with the Distiller pipeline (https://github.com/open2c/distiller‐nf). HiCRep (v0.2.6) [73] was used to analyze the correlation efficiency of library replicates for each sample, and then pool the data from replicates together for further analysis. After pooling, the valid pairs were binned into nonoverlapping genomic intervals (5, 10, 20, 40, 100 and 200 kb) to generate a contact map. Finally, the contact maps were normalized to eliminate systematic biases using the iterative normalization method. The map resolution of the Hi‐C map was defined as the bin size used to construct a particular contact matrix, namely the smallest locus size such that 80% of bins have at least 1,000 contacts [74].

### 5.6 Experiment and Analysis of ATAC‐seq

The breast muscles of the same accessions in genomic assembly were subjected to ATAC‐seq. Two replicates were performed for each accession. Approximately 50,000 nuclei were allocated to perform tagmentation according to, and Tn5 transposed DNA were purified by AMPure DNA magnetic beads (Beckman, CA, USA). Amplified libraries were run on an Agilent Tapestation 2100 (Agilent Technologies, Böblingen, Germany) using a D5000 DNA ScreenTape to assess quality. The final library was sequenced on Illumina NovaSeq 6000 platform (Illumina, CA, USA) with paired‐end 150‐bp reads. FastQC (v0.11.9) and Trimmomatic (v0.39) were used to perform quality control, trim any remaining adapters and low‐quality bases, and remove reads shorter than 8 nt. Clean reads were aligned to both their corresponding genome and T2T genome [8] using Bowtie2 (v2.4.5) [75] and the following options ‐X 2000 –very‐sensitive. MarkDuplicates (Picard) was used to remove PCR duplicates. ATAC peaks were called using MACS2 (v2.1.4) [76] with parameters ‘‐f BED –nomodel –extsize 150 –shift ‐75 –keep‐dup all’. Peaks were filtered at a q‐value below 0.05. Bedtools (v2.31.1) [77] was used to merge peaks from each replicate and accession into a union set. Peaks were annotated to the nearest feature using ChIPseeker (v1.32.0) [78]. Differentially accessible regions (DAR) were identified using DESeq2 (v1.18.1) [79]. Regions with log2FoldChange ≥ 1 and *p* value < 0.05 were considered differentially accessible. Normalized ATAC‐seq signals were derived from bigwig files using bedGraphToBigWig.

### 5.7 Experiment and Analysis of RNA‐seq

The breast muscles of the same accessions in genomic assembly were subjected to RNA‐seq. Three replicates were performed for each accession. The total RNA was subjected to construct a library using NEBNext UltraTM RNA Library Prep Kit (Illumina, CA, USA) according to the standard protocol. Fragments of ≈200 bp were selected using AMPure XP beads (Beckman, CA, USA), amplified by PCR, and purified to obtain the final library. The libraries were sequenced on the Illumina NovaSeq 6000 platform (Illumina, CA, USA). HISAT2 (v2.2.1) [80] was used to align clean reads to both their corresponding genome and T2T genome [8] with the parameters ‘–phred33 –no‐mixed –no‐discordant’. FeatureCount (v2.0.1) [81] was used to calculate the read counts of each gene, and TPM values were calculated for normalization by both gene length and sequencing depth. Differentially expressed genes (DEGs) were identified using DESeq2 (v1.18.1) [79]. The obtained *p* values were adjusted using Benjamini and Hochberg's method to control the false discovery rate. Genes with |log2(FoldChange)| > 1.0 & p‐adj ≤ 0.05 were considered differentially expressed

### 5.8 Genome Assembly, Assessment and Annotation

The HiFi reads were assembled into contigs using Hifiasm (v0.19.5) [82] with default parameters. These contigs were anchored to chromosome‐level assemblies using the Juicer/JuiceBox/3D‐DNA pipeline. Briefly, Hi‐C reads of each breed were filtered to obtain valid pairs using Juicer software (v1.6) [83]. The 3D‐DNA (https://github.com/aidenlab/3d‐dna) was used to conduct Hi‐C‐assisted assembly to anchor contigs onto the chromosome. JuicerBox (v2.13.07) [84] was used to manually adjust and correct the placement and orientation errors in chromatin's Hi‐C interaction patterns. The completeness of 15 genome assemblies was assessed with BUSCO (v5.1 and the aves_odb10 database) [85] with the parameters ‘‐m genome –augustus’. The short‐read data of each breed was aligned to their genome assembly to inquire mapping rate and genome coverage using BWA (v0.7.17–r1188) [86]. The collinearity analyses of the T2T genome and 15 newly assembled genomes were performed using JCVI (v1.4.5) [87] with default parameters. Homology‐based comparison and de novo structure analyses were performed for repeat annotation, including RepeatMasker and RepeatProteinMask (open‐4.0,9) [88] with the Repbase TE library [89, 90], RepeatScout (v1.0.5) [91], RECON (v1.08) [92], LTR_FINDER (v1.0.7) [93], and Tandem Repeats Finder (v4.09) [94]. After masking the genome using RepeatMasker (open‐4.0,9) [88], de novo gene prediction was performed using Augustus (v3.3.1) [95] and Genescan (v1.0) [96]. For homology‐based gene prediction, protein sequences were aligned to the genome assembly and predicted coding genes using Exonerate (v2.2.0) [97] with default parameters. RNA‐seq (pooling sequencing of mixed tissue) assisted gene prediction was carried out using TopHat (v2.1.1) [98] and Cufflinks (v2.2.1) [99]. Finally, MAKER (v3.00) [100] was used to integrate the prediction results of the three methods to predict gene models. Gene function was annotated using InterPro, GO, KEGG, NR, and UniProt databases, respectively [101, 102, 103, 104]. For non‐coding RNA annotation, tRNA was predicted using tRNAscan‐SE (v1.3.1) algorithms [105] with default parameters. rRNA sequences from the Ensembl database were aligned against our genome using BLASTN [106] with a cutoff of E‐value < 0.00001, identity ≥ 85% and match length ≥ 50 bp to predict rRNA. The miRNAs and snRNAs were identified by Infernal (v1.1.2) [107] against the Rfam (v14.1) database [108] with default parameters.

### 5.9 Gene‐Based Pan‐Genome Construction

Except for 15 newly generated *de novo* genome assemblies, 13 published high‐quality chicken assemblies were also integrated in this study, including Red jungle fowl (GCA_000002315.5), Cobb (GCA_027408225.1), Ross (GCA_027557775.1), Commercial broiler (GCF_016699485.2), White leghorn (GCF_016700215.2), Silkie (GCA_033088195.1), Piao (GCA_030914265.2), DWS (GCA_030849555.2), Tibetan (GCA_025370635.1), Houdan (GCA_024653045.1), Cornish (GCA_024653035.1), Rhode Island Red (GCA_024652985.1) and T2T genome (GCA_024206055.2) [8, 17, 18, 32, 33] (Table S1). The longest transcript of each gene was chosen to perform gene clustering analysis to classify the gene family using OrthoFinder (v2.5.2) [109] with the following parameters: ‘‐M msa ‐S diamond’. Based on single‐copy orthologous genes from gene clustering analysis, the phylogenetic tree was constructed using RAxML (v8.2.12) [110] with the PROTGAMMAAUTO model, and the GRCg6a was used as outgroup. The divergence time was estimated using MCMCtree in the PAML (v4.8) [111]. According to the published research, the divergence time calibration point of the outgroup was selected from 9500 years ago to 3500 years ago [4]. The expansion and contraction of gene family number were calculated using CAFE (v5.1.0) [112] with default parameters. Core and pan gene families were counted based on the results of gene family clustering in 28 chicken assemblies. Gene families were divided into the following four types: Core families, Softcore families, Private families, and Dispensable families, referring to families detected in all assemblies, ≥ 90% of assemblies, only one specific assembly, and others, respectively.

### 5.10 Variation Calling and Analysis of Pan‐SV

For SNPs and InDels detection, high‐quality reads were aligned to the T2T genome using Minimap2 (v2.10) [113] with the parameters of ‘‐ax map‐pb ‐k 19 ‐O 5,56 ‐E 4,1 ‐B 5’. SNPs and InDel were called from the resulting BAM files using the show‐snps tools in MUMmer (v3.1) with the parameters of ‘‐ClrT’. For SV detection, the 27 genome accessions were aligned to the T2T genome using the NUCMER tool in MUMmer (v3.1) [114] with the parameters of ‘–maxmatch ‐l 100 ‐c 100’ and identified SV using Assemblytics (v1.2.1) [115] with the parameters of ‘10000 50 100000’. Besides, we also employed PBSV (v2.8.0) with ‘‐m 50 ‐A 3 ‐O 3’ parameters and Sniffles (v2.0.7) [116] with default parameters to identify SV, and only concordant SVs detected by both tools were retained. Finally, SVs detected from the assembly‐based pipeline and long‐read‐based pipeline were merged using SURVIOUR (v1.0.7) [117] with the parameters of ‘1000 1 1 0 0 50’ to construct a non‐redundant SV set. SVs were divided into five types: Deletion (DEL), Insertion (INS), Inversion (INV), Duplication (DUP), and Translocation (TRAN). BAM files from long‐read sequencing were subjected to manual inspection using Integrative Genomics Viewer (IGV). SVs were randomly selected per assembly, with GRCg6a excluded due to insufficient sequencing depth. Genes located within 5 kb upstream and downstream of an SV's breakpoints were defined as SV‐gene according to the previous study [118]. The SV hotspot was identified by calculating the distribution of SV breakpoints for each 100‐kb window with a 100‐kb step size along each chromosome according to previous research [36]. The LD analysis between SVs and SNPs was performed and visualized using PLINK (v1.90b6.10) [119]. The classification criteria for pan‐SV align with those for pan‐gene families. Based on published workflows [36,120] and Meerkat (v0.189) [121], we developed a custom script to identify the driving formation mechanism of SVs. A total of six detailed mechanism types were identified in chicken's SV, including transposable element intersection (TEI), non‐homologous end joining (NHEJ), variable number of tandem repeats (VNTRs), fork stalling and template switching/microhomology‐mediated break induced repair (FoSTeS/MMBIR), nonallelic homologous recombination (NAHR), and alternative end joining (alt‐EJ). The detailed identification standard of those mechanisms was described in a previous work [36]. The BEDTools (v2.31.1) [77] tool was used to calculate the TE coverage of SVs driven by different mechanisms.

### 5.11 Identification of 3D Chromatin Architectures

The 3D chromatin architectures were identified based on the mapping data of both their corresponding genome and the T2T genome, respectively. A/B compartments and TAD boundaries were delineated in Hi‐C interaction matrices with 100‐kb and 40‐kb resolution using Cooltools (v0.4.1) [122], respectively. For the compartment, each bin with a positive or negative first eigenvector was divided into A or B, and the continuous A or B bins were merged to obtain A or B compartment [123]. For TAD, the insulation score [124] of each bin was obtained by sliding window approaches. The threshold for TAD boundary strength was determined by threshold_li. TADs were identified as the regions between two adjacent boundaries. The contacts between 10‐kb bins derived from inter‐chromosomal interactions were transferred into FitHiC2 (v2.0.8) [125] to identify significant chromatin loops for each accession using a threshold of qvalue < 1e‐6. LT‐CREs were identified as described in published work [26], namely the regions was overlapped with ATAC peaks, located at least 2 kb away from the transcription start site (TSS) of a gene, and covered by at least one loop anchor. The anchor on a loop overlapping with at least an LT‐CRE was defined as an LT‐CRE anchor (E), while an anchor only overlapping within 2kb upstream of the TSS of a gene was defined as a Gene anchor (G). Based on this classification, we divided the loops into E‐E, E‐G, and G‐G types.

### 5.12 Conservation Analysis of 3D Genome

We performed conservation analysis of chromatin architectures in pan‐3D genome according to published pipelines [26, 27, 126]. Based on the pan‐3D genome, we used OrthoFinder (v2.5.2) [109] to identify single‐copy orthologous genes in 15 newly generated genome assemblies, and their compartmentalization status (A/B) was compared across all 15 genomes to classify conservative and variable compartments. For conservation analysis of TAD boundaries, loops and LT‐CREs, we aligned 15 newly generated genomes to the T2T genome using Minimap2 (v2.10) [113] with the parameters of ‘‐ax map‐pb ‐k 19 ‐O 5,56 ‐E 4,1 ‐B 5’. Chain files were generated using Transanno (v0.4.5) (https://github.com/informationsea/transanno) with the default parameters. TAD boundaries, loop anchors and LT‐CREs for each genome were converted to T2T genome coordinates through the chain files using Transanno (v0.4.5). For TAD boundaries, Bedtools (v2.31.1) [77] was used to merge the converted boundary regions, allowing a maximum gap of 40 kb between adjacent boundaries. For TAD domain reorganization events, BEDTools (v2.31.1) [77] was used to calculate the proportion of overlapped TADs between HYB and the other 14 accessions and divided them into 3 types, including Fusion, Neo, and Stable, according to the published method [127]. For chromatin loops, a loop was considered conserved between two genomes if both of its mapped anchors corresponded and overlapped by more than 50%. For LT‐CREs, the set of pan LT‐CREs was generated and classified using the same pipeline as for pan‐TAD boundaries, allowing a maximum gap of 500 bp between adjacent LT‐CREs. Based on these, we generated sets of pan‐TAD boundaries, loops, and LT‐CREs across 15 genomes, and further categorized them into core (all genomes), softcore (> 90% of genomes), dispensable, and private (species‐specific) types. Core and softcore features were defined as highly conserved, while dispensable and private features were defined as poorly conserved. As a comparison, we also conducted conserved analysis of 3D genome based on conventional single reference genome. For a single T2T reference, the conservative compartments were defined as compartment bins with the same status in all 15 accessions, and the variable compartments referred to the compartment bins with different status in at least one accession. We further merged the coordinates of TADs, loop anchors, and LT‐CREs from 15 accessions onto the T2T genome. The classification methodology of pan‐type was identical to that used for the pan‐3D genome.

### 5.13 SV Distribution in 3D Chromatin Architectures

For TAD domains and boundaries change events and four types of pan‐TAD boundaries, we investigated the distribution of observed and expected values of SV coverage in these regions using BEDTools (v2.31.1) [77], and the random TAD domain and boundary regions were generated 1,000 times in the genome by the boot‐strapping method. We also investigated the coverage of SV in the four types of pan‐loop anchors using BEDTools (v2.31.1) [77], Loops with at least one anchor overlapped by an SV were defined as an SV‐loop, and genes located on the opposite anchor were defined as SV‐loop genes.

### 5.14 Graph‐Based Pan‐Genome Construction

For graph‐based genome construction and variation detection, we selected the T2T genome to serve as the backbone of the graph structure using vg tools (v1.53.0) [128]. The non‐redundant deletions and insertions were saved to perform the graph‐based genome construction using the vg construct tool with the parameters of ‘‐a ‐f ‐S’. The sequenceTubeMap (https://github.com/vgteam/sequenceTubeMap) was used to visualize the graph‐based pan‐genome.

### 5.15 SNPs and InDels Calling for Population

For 858 global individuals and 877 F2 individuals, we aligned the filtered reads to the T2T genome using BWA (v0.7.17‐r1188) [86] with the parameters of ‘‐M ‐Y’. SNPs and InDels were genotyped using HaplotypeCaller tools in the GATK package (v4.2.2.0) [129] with the parameters of ‘‐ERC GVCF’.

### 5.16 SVs Calling for Population

The clean reads of 858 global individuals and 877 F2 individuals were aligned to our graph‐based pan‐genome using the vg giraffe tool [130] with default parameters. Then, based on the GAM‐format alignment file, the compressed coverage index was calculated using vg pack tool with the parameters of ‘‐Q 5 ‐s 5’. The snarls were generated using the vg snarls tools with default parameters. The SV genotyping of 1,735 chicken accessions was performed using the vg call tool [131] with the parameters of ‘‐a’. The quality control for SVs derived from the graph‐based pan‐genome was implemented with thresholds: DP > 2 and GQ > 10.

### 5.17 Population Genetic Analysis

The SNP and SV markers of both populations were filtered by PLINK (v1.90b6.12) [119] using ‘–geno 0.2 –maf 0.05’ and ‘–geno 0.5 –maf 0.05’ parameters, respectively. Neighbor‐Joining (NJ) tree was built and visualized using VCF2Dis (v1.54) [132], FastMe 2.0 [133], and iToL [134]. Principal‐component analysis (PCA) was performed using PLINK (v 1.90b6.10) [119]. Genetic structure clustering was performed using the ADMIXTURE program after filtering high‐LD loci by PLINK (v 1.90b6.10) [119] with ‘–indep‐pairwise 50 10 0.1’ parameters. LD decay analysis was performed using PopLDdecay (v3.43) [135].

### 5.18 Selective Signal Analysis

For selective signal analysis, the comparisons were made between RJF and domestic chicken. The samples of other jungle fowls were excluded. The weighted fixation statistics (Fst) and nucleotide diversity (Pi) of SV and SNP markers were calculated using the VCFtools (v0.1.17) package [136]. We calculated the Fst statistic and the Pi‐ratio (RJF/domestic chicken) for each SV marker. SNPs were performed using 50‐kb sliding windows with a shifting increment of 25‐kb step size.

### 5.19 Genome‐Wide Association Analysis

SNPs and SVs were screened across 877 individuals of the F2 population using PLINK (v1.90b6.10) [119] with the parameters ‘–geno 0.2 –maf 0.05’ for SNPs and ‘–geno 0.5 –maf 0.05’ for SVs, respectively. The genetic variations on sex chromosomes were excluded. SNP‐GWAS were performed using a univariate Linear Mixed Model (LMM) in GEMMA (v0.98.5) [137] with the parameters of ‘‐miss 1 ‐maf 0 ‐lmm 1 ‐c’. SV‐GWAS were performed using the fixed and random model Circulating Probability Unification (FarmCPU) [138] model in rMVP package (v1.4.0) [139]. The kinship matrix was constructed using all genome‐wide SNPs and SVs, excluding those on the sex chromosomes. Population stratification and genetic relatedness were corrected by the first three principal components (PCs) derived from SNPs and SVs (without sex chromosomes), as well as the kinship matrix, as covariates. Furthermore, the gender effect was adjusted by adding gender information as a covariate. The λ values and quantile‐quantile (QQ) plots were used to verify whether the models were overfitted or not. The significance threshold for both SNP and SV‐GWAS was determined based on the effective number of independent markers. The SNP and SV markers were pruned using PLINK (v1.90b6.10) [119] with the parameters ‘–indep‐pairwise 50 10 0.2’ to screen the independent genetic markers. The significance threshold was self‐defined as *p* < 1/N, where N referred to the number of markers remaining after LD pruning. The Manhattan and QQ plots of GWAS results were visualized using the CMplot package [139].

### 5.20 Genome Selection Analysis

The SNPs, InDels, and SVs datasets of 877 individuals in the F2 population were used to conduct genomic selection (GS) model prediction. The missing genotypes were imputed using Beagle (v4.1) [140] with default parameters. Biallelic loci were kept and further filtered using PLINK (v1.90b6.12) [119] with the parameters of ‘–geno 0.2 –maf 0.05’ for SNPs and Indels, and ‘–geno 0.5 –maf 0.05’ for SVs. SNPs and InDels were pruned to exclude high‐linkage markers using PLINK (v1.90b6.12) [119] with ‘–indep‐pairwise 50 5 0.2’ parameters. The Genomic/Single‐step Best Linear Unbiased Prediction (GBLUP and ssBLUP) model was constructed using the sommer package [141] in R program and HiBLUP (v1.4.0) [142], respectively. Besides, we also performed Ridge Regression Best Linear Unbiased Prediction (rrBLUP), Bayesian Regression Model B (BayesB), Random Cluster Forests (RCF) and Support Vector Classification (SVC) models using G2P packages [143] in R program. Among them, GBLUP model utilized the genetic relationship matrix (G matrix) to model the covariance between genomic breeding values, while ssBLUP integrated the G matrix and pedigree‐derived additive genetic relationship matrix (A matrix) into the hybrid relationship matrix (H matrix). The predictive accuracy was performed via a ten‐fold cross‐validation method with ten repetitions according to the previous study [144]. Briefly, the whole dataset of 877 individuals was randomly divided into ten subsets. For each iteration, one subset served as the validation set while the remaining four subsets served as the training sets. This process was repeated ten times, ensuring each subset was used for the validation set once. The averaged predictive accuracy across ten iterations was considered the final predictive accuracy.

### 5.21 PCR Validation of SVs

The DNA samples were extracted from the chicken brood from the validation population using the NRBC Blood DNA Kit (Omega, Georgia, CA). The PCR experiment was performed using KOD One PCR Master Mix‐Blue (Toyobo, Osaka, Japan) according to the manufacturer's protocol. Agarose gel electrophoresis was employed to genotype the individuals for the above three populations. PCR primers were designed flanking the breakpoints of candidate SVs. The information on primers was listed in Table S40. The PCR products were sequenced using ABI‐3730XL by Tsingke Biotech Co., Ltd (Beijing, China).

### 5.22 RNA extraction, cDNA synthesis, and qPCR

Breast muscles of individuals in the indigenous population (Foshan Nanhai Poultry Breeding Co., Ltd, Guangdong, China) were selected to extract RNA, synthesize cDNA, and perform qPCR. Phenol‐chloroform extraction was conducted to isolate and purify the total RNA of breast muscles using RNAiso Plus (Takara, Kyoto, Japan) and HiPure Universal RNA Mini Kit (Magen, Guangzhou, China). Reverse transcription PCR was performed to synthesize cDNA using MonScript 5× RTIII All‐in‐one Mix kits (Monad, Shanghai, China). After extraction and synthesis, RNA and cDNA samples were stored in −80°C freezers. qPCR was performed using ChamQ Universal SYBR qPCR Master Mix (Vazyme Biotech, Nanjing, China) on a QuantStudio5 Real‐Time PCR system (Applied Biosystems, Carlsbad, CA). The 2^−∆∆Ct^ method [145] and internal normalization were performed to analyze quantification results. *GAPDH* was employed as a housekeeping gene. The information on primers was listed in Table S40.

### 5.23 Experiment and Analysis of Capture Hi‐C

To validate the role of the SV upstream of *KLF3* in rewriting chromatin loops, two replicates of breast muscle from each of the WT and MT genotypes were selected to construct Capture Hi‐C libraries and compare the different landscapes of loops mediated within the target SV region. The assayed samples were derived from the indigenous chicken population (Foshan Nanhai Poultry Breeding Co., Ltd, Guangdong, China) sharing the same genetic background. Before library construction, the SV genotype was confirmed by PCR validation. Libraries were constructed according to previous studies [74, 146]. The DNA baits were subjected to capture target regions of the Hi‐C library using QuarHyb DNA Plus 3Reagent Kit (Dynegene Technologies, Shanghai, China) according to the manufacturer's protocol. After library enrichment, the post‐capture PCR amplification was carried out, purified with DNA clean beads, determined by Bioanalyzer profiles (Agilent Technologies, Böblingen, Germany) and sequenced on the MGI‐seq T7 platform with PE150 mode. (BGI‐Tech, Shenzhen, China). Raw sequencing reads were processed and mapped di‐tags against the T2T genome using the standard HiCUP pipeline [147]. The Interaction confidence scores were determined using the CHiCAGO pipeline at the binned level [148]. Interactions with a CHiCAGO score ≥ 3 were considered high‐confidence interactions. Based on the score of each interaction across groups, we screened differentially enhanced interactions between groups. For MT vs. WT, an MT's different interaction was defined as: (1) significant interactions in MT, (2) non‐significant interactions in WT, (3) MT score − WT score > 3, and vice versa.

### 5.24 Dual‐Luciferase Reporter Assay

Sequences containing the entire SV sequence were inserted into the pGL3 promoter to assess its activity in chicken primary myoblast. The chicken primary myoblast was isolated and cultured from the E11 chicken leg muscle according to our previous studies [149, 150]. Dual‐luciferase reporter assays were performed using the Dual‐luciferase reporter assay system (Promega, Madison, WI, USA) according to the manufacturer's instructions. The pGL3‐basic vectors were co‐transfected with pRL‐TK as a control. Firefly and Renilla luciferase activities were measured according to our previous method [151]. All the data were acquired by averaging the results from six independent replicates.

### 5.25 Statistical Analysis

Details on statistical analyses used in our study, including the statistical tests used and the number of replicates, were provided in the corresponding figure legends. Statistical analysis and data visualization were performed using R (v.4.4.2), Python (v.3.13.2), Microsoft Excel 2021 and TBtools‐II [152].

## Author Contributions

Z.Z. contributed to writing the original draft, writing – review and editing, formal analysis, visualization, and software development. D.C. contributed to writing – review and editing, formal analysis, visualization, and software development. C.Z. contributed to formal analysis, visualization, and software development. S.Z. contributed to formal analysis and software development. J.L. and Z.Z. contributed to validation and investigation. S.K. and X.Y. contributed to formal analysis and investigation. X.Z. and F.B. contributed to writing – review and editing. F.C., Y.X., and Z.Z. contributed to software development. L.G. and Z.L. contributed to resources and data curation. X.Z. contributed to conceptualization, supervision, and resources. W.L. contributed to conceptualization, writing – review and editing, project administration, supervision, resources, data curation, and funding acquisition. J.S. and B.C. contributed to conceptualization, writing – review and editing, project administration, supervision, resources, and data curation. Q.N. contributed to conceptualization, writing – review and editing, project administration, supervision, resources, data curation, and funding acquisition.

## Ethics Statement

All animal experiments performed in this study satisfied the requirements of the Institutional Animal Care and Use Committee at South China Agricultural University (Approval ID: SCAU‐2024F018). Experimental animals were slaughtered following the Chinese national standard (GB/T 19478‐2018).

## Funding

This work was supported by Biological Breeding‐National Science and Technology Major Project (2023ZD04064), National Key R&D Program of China (2021YFD1300100), National Natural Scientific Foundation of China (U24A20440 and 32302728), China Agriculture Research System of MOF and MARA (CARS‐41), Science and Technology Program of Guangdong Province (2023B1212060057), and Project of the Seed Industry Revitalization of Department of Agriculture (2024‐XPY‐00‐003).

## Conflicts of Interest

The authors declare no conflict of interest.

## Supporting information




**Supporting File 1**: advs74431‐sup‐0001‐SuppMat.pdf.

**Supporting File 2**: advs74431‐sup‐0002‐TableS1‐S40.xlsx.

**Supporting File 3**: advs74431‐sup‐0003‐DataFile.txt.

## Data Availability

The datasets and genomes newly generated in this study have been deposited in the Genome Sequence Archive (GSA) and Genome Warehouse (GWH) database (https://ngdc.cncb.ac.cn) under accession code BioProject PRJCA043752. All raw sequencing data (HiFi, Hi‐C, ATAC‐seq, RNA‐seq, Capture Hi‐C) in this study have been deposited in the GSA database (ID: CRA028625, CRA030010, CRA029313, CRA028664, CRA029337, CRA036611). The newly assembled genomes are available in the GWH database (ID: PRJCA043752). The T2T genome, 12 published de novo assemblies, and short‐read resequencing data of 858 global individuals are obtained from previous studies [4, 8, 17, 18, 32, 33]. The vg files of our graph‐based pan‐genome have been deposited in our GitHub repository. All software and packages used in the study are publicly available from the Internet as described in the Experimental Section. The customized scripts and codes used in the present study are available under the MIT license in GitHub repository at https://github.com/ZhenZhou0720/Pan3D‐Chicken.
