# Supplementary material for: Pan‐3D Genome Analysis Reveals the Roles of Structural Variation in Chicken Chromatin Architectures, Domestication and Production Traits
Source: Adv Sci (Weinh). 2026 Feb 15;13(24):e20068. doi: 10.1002/advs.202520068 (PMC13116375; doi:10.1002/advs.202520068)
Supplement: Supplementary file 1 — Supporting File 1: advs74431‐sup‐0001‐SuppMat.pdf. [file ADVS-13-e20068-s001.pdf]

Supporting Information

Pan-3D Genome Analysis Reveals the Roles of Structural Variation in Chicken Chromatin Architectures, Domestication and Production Traits

Zhen Zhou [1], Danfeng Cai [1], Changbin Zhao [1], Siyu Zhang [1], Jiahao Li [1], Zhaofeng Zhang [1], Shaofen Kong [1], Xin Yang [1], Xiaoli Zhou [1], Farhad Bordbar [1], Fayi Chen [4], Yaohuan Xu [4], Zhe Zhang [5], Lihong Gu [3], Zhenhui Li [1], Xiquan Zhang [1], Wen Luo [1,], Jingting Shu [2,*], Bolin Cai [1,*], Qinghua Nie [1,*]*

1. State Key Laboratory of Swine and Poultry Breeding Industry, Guangdong Laboratory for Lingnan Modern Agriculture, Guangdong Provincial Key Lab of Agro-Animal Genomics and Molecular Breeding, Key Laboratory of Chicken Genetics, Breeding and Reproduction, Ministry of Agriculture and Rural Affairs, National-Local Joint Engineering Research Center for Livestock Breeding, College of Animal Science, South China Agricultural University, Guangzhou, China.
2. Key Laboratory for Poultry Genetics and Breeding of Jiangsu Province, Jiangsu Institute of Poultry Science, Yangzhou, China.
3. Institute of Animal Science & Veterinary Medicine, Hainan Academy of Agricultural Sciences, Haikou, China.
4. Wuhan Generead Biotechnology Co., Ltd, Wuhan, China.
5. Huazhi Biotechnology Co., Ltd, Changsha, China.

* **Correspondence** should be addressed to **Qinghua Nie**, **Bolin Cai**, **Jingting Shu**, and **Wen Luo**.

(1) Qinghua Nie (nqinghua@scau.edu.cn): College of Animal Science, South China Agricultural University, Guangzhou 510642, China.

(2) Bolin Cai (bolincai@scau.edu.cn): College of Animal Science, South China Agricultural University, Guangzhou 510642, China.

(3) Jingting Shu (sjt@jips.cn): Poultry Institute, Chinese Academy of Agricultural Sciences, Yangzhou, 225125, China.

(4) Wen Luo (luowen729@scau.edu.cn): College of Animal Science, South China Agricultural University, Guangzhou 510642, China.

This Supporting Information file includes:

- Supplementary Figure S1 to S27 (This file)
- Supplementary Table S1 to S40 (Separate EXCEL file)
- Supplementary file of Table S21 (Separate TXT file)

Table of Contents

Legends for Supplementary Figures S1-S27

Figure S1. Correlation analysis of genetic features between 28 genome assemblies and 858 global individuals.

Figure S2. Expansion and contraction of gene families in chicken.

Figure S3. Functional enrichment of expanded and contracted gene families.

Figure S4. Functional enrichment of core and dispensable gene families and SVs.

Figure S5. Enrichment analysis of novel sequences in 27 accessions compared to T2T assembly.

Figure S6. Characteristics of SVs in chicken.

Figure S7. Characteristics of pan-SVs in chicken.

Figure S8. Correlation among Hi-C libraries and available resolutions.

Figure S9. Circos plots showing the landscape of 3D chromatin architectures for 15 accessions based on their corresponding genomes.

Figure S10. Identification of 3D chromatin architectures based on single T2T genome and their corresponding genome

Figure S11. Characteristics of TAD reorganization events in chicken.

Figure S12. Significantly functional enrichment of genes in different pan-loops.

Figure S13. Integrative genomics viewer of 240-bp INS of *TSHR* genes in Pan-SV database.

Figure S14. Population genomic analysis of F2 population based on SVs and SNPs.

Figure S15. Summary of SV-GWAS in 49 production traits of F2 population.

Figure S16. Manhattan plots of SNP and SV-GWAS for 12 growth performance traits.

Figure S17. QQ plots of SV-GWAS for 12 growth performance traits.

Figure S18. QQ plots of SNP-GWAS for 12 growth performance traits.

Figure S19. Manhattan plots of SNP and SV-GWAS for 8 meat-quality performance traits.

Figure S20. QQ plots of SV-GWAS for 8 meat-quality performance traits.

Figure S21. QQ plots of SNP-GWAS for 8 meat-quality performance traits.

Figure S22. Manhattan plots of SNP and SV-GWAS for 25 carcass performance traits.

Figure S23. QQ plots of SV-GWAS for 25 carcass performance traits.

Figure S24. QQ plots of SNP-GWAS for 25 carcass performance traits.

Figure S25. PCR and Sanger sequencing validation of partial candidate structural variations.

Figure S26. *KLF3*, *Tbc1d1* and *PGM2* expression profiles across different SV genotypes and stages of broiler growth and myoblast development.

Figure S27. Characterization of Capture Hi-C data in KLF3-SV wild-type and mutant-type samples.

Supplementary Tables S1-S40 (detailed in separate EXCEL file)

Table S1. Information on 28 chicken assemblies used in pan-genome analysis.

Table S2. Summary statistics of HiFi data generated in this study.

Table S3. Quality assessment of Hi-C data generated in this study.

Table S4. Summary statistics of clean Hi-C data generated in this study (aligned against the T2T genome and their corresponding genomes).

Table S5. Quality assessment of ATAC-seq data generated in this study.

Table S6. Summary statistics of clean ATAC-seq data generated in this study (aligned against the T2T genome and their corresponding genomes).

Table S7. Quality assessment of RNA-seq data generated in this study.

Table S8. Summary statistics of clean RNA-seq data generated in this study (aligned against the T2T genome and their corresponding genomes).

Table S9. The information on bait fragments used in Capture Hi-C.

Table S10. Quality assessment of Capture Hi-C data generated in this study.

Table S11. Summary statistics of clean Capture Hi-C data generated in this study.

Table S12. Genome assembly quality assessment of newly assembled genomes.

Table S13. Gene, repeat, and non-coding RNA annotations of newly assembled genomes.

Table S14. Gene family classification across 28 chicken genomes.

Table S15. Pan-gene family classification across 28 chicken genomes.

Table S16. Non-redundant structural variations (SVs) in pan genome

Table S17. Classification results of pan compartment based on pan-3D genome.

Table S18. Classification results of pan compartment based on T2T genome.

Table S19. Classification results of pan-TAD boundary based on pan-3D genome.

Table S20. Classification results of pan-TAD boundary based on T2T genome.

Table S21. Classification results of pan chromatin loops based on pan-3D genome.

Table S22. Classification results of pan chromatin loops based on T2T genome.

Table S23. Classification results of pan LT-CRE based on pan-3D genome.

Table S24. Classification results of pan LT-CRE based on T2T genome.

Table S25. TAD reorganization compared to HYB genome.

Table S26. QC and mapping statistics of 858 global individuals in the graph-based pan-genome.

Table S27. QC and mapping statistics of 877 F2 individuals in the graph-based pan-genome.

Table S28. The statistics of genome variations identified in this study.

Table S29. Structural variations (SVs) were used for selective signal analysis in 858 individuals (MAF>0.05).

Table S30. Structural variations (SVs) were used for SV-GWAS of 49 production traits in 877 F2 individuals (MAF>0.05).

Table S31. Genomic inflation factor of SV-GWAS and SNP-GWAS in production traits of 877 F2 individuals.

Table S32. Candidate structural variations (SVs) associated with chicken domestication.

Table S33. Candidate SNP windows associated with chicken domestication.

Table S34. Candidate structural variations (SVs) significantly associated with production traits in the 877 F2 population.

Table S35. Candidate SNPs significantly associated with production traits in the 877 F2 population.

Table S36. Candidate SV-loop genes associated with chicken domestication.

Table S37. Candidate SV-loop genes significantly associated with production traits in the 877 F2 population.

Table S38. The significant and differentially enhanced loops identified in Capture Hi-C.

Table S39. Predictive accuracy of genomic selection using different combinations of genetic variations.

Table S40. The information on primers used in this study.

Supplementary file of Table S21 (detailed in a separate TXT file)

Details of the clustering result of chromatin loops in chicken pan-3D genome

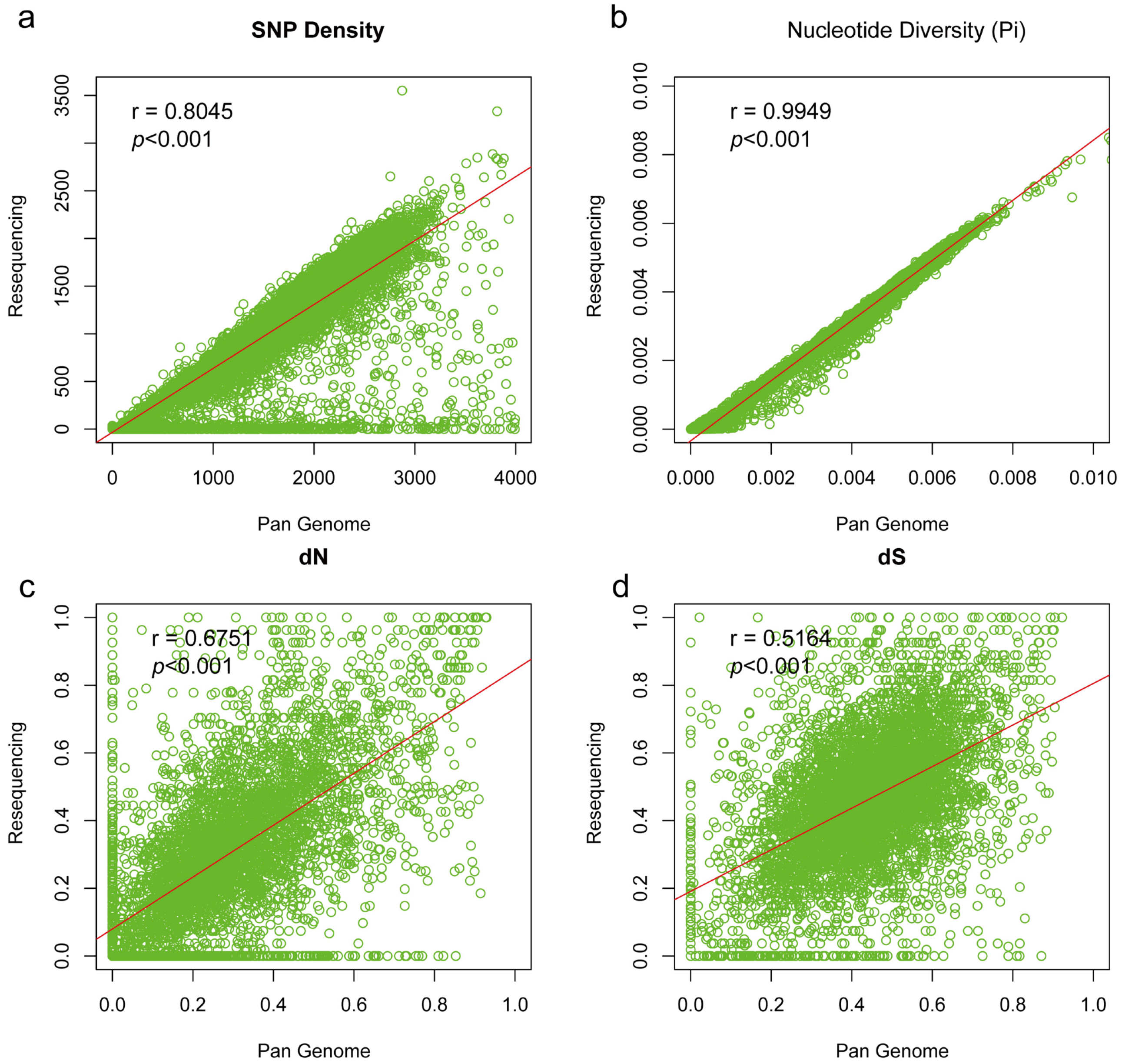

Figure S1. Correlation analysis of genetic features between 28 genome assemblies and 858 global individuals. Spearman's correlation of a) SNP density, b) nucleotide diversity, c) dN, and d) dS between 28 assemblies and 858 global individuals.

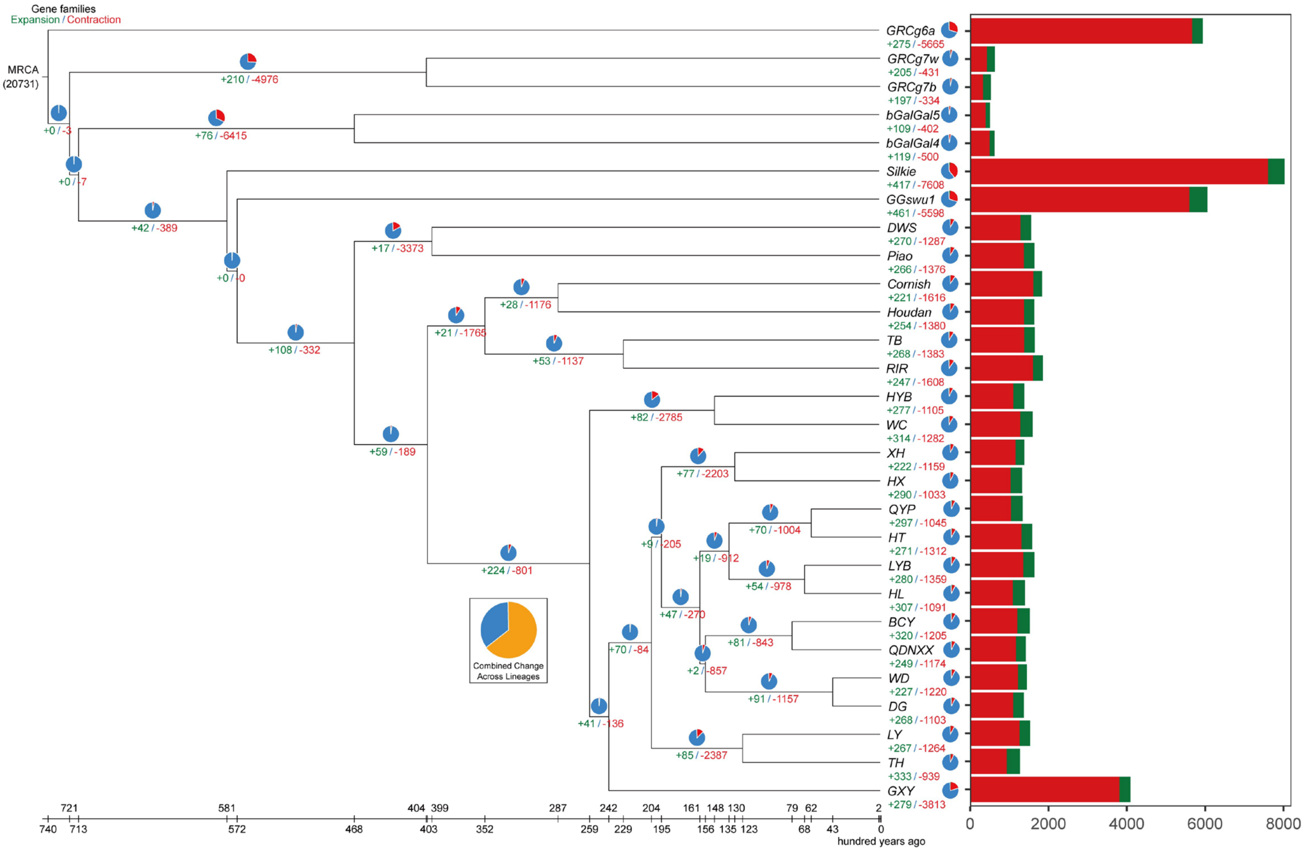

Figure S2. Expansion and contraction of gene families in chicken. Green and red numbers on the phylogenetic tree represent the counts of expanded and contracted gene families for each accession.

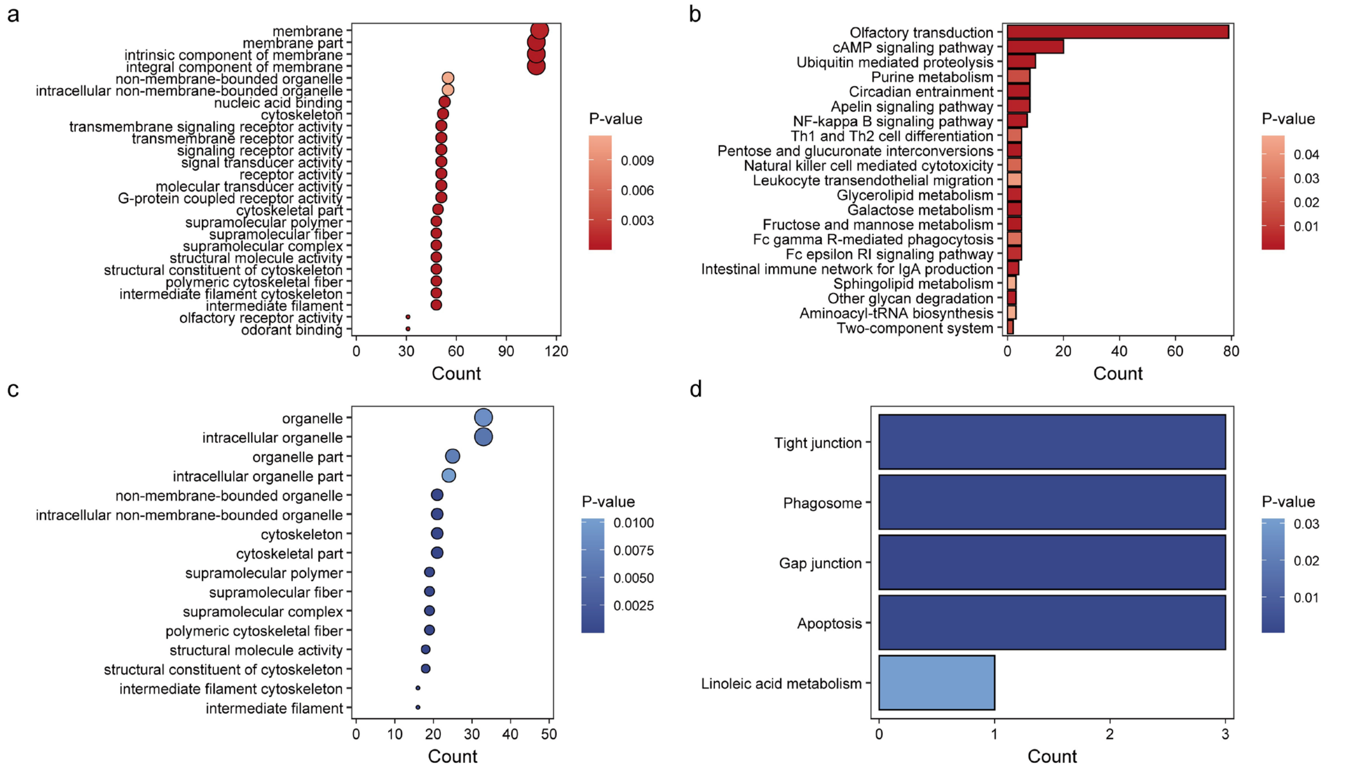

Figure S3. Functional enrichment of expanded and contracted gene families. a) GO and b) KEGG enrichment of expanded gene families. c) GO and d) KEGG enrichment of contracted gene families.

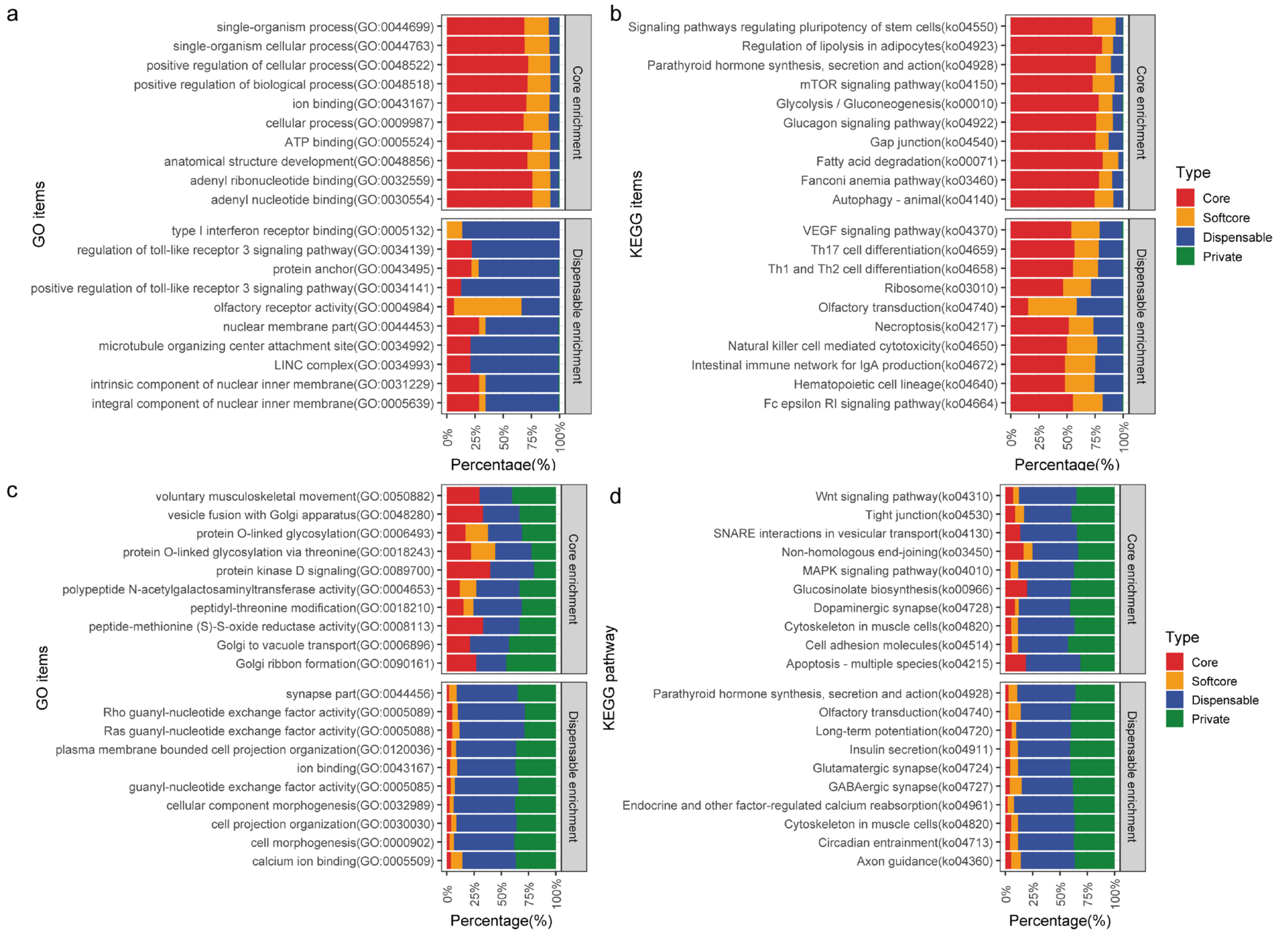

Figure S4. Functional enrichment of core and dispensable gene families and SVs. a) GO and b) KEGG enrichment analysis of core and dispensable gene families. c) GO and d) KEGG enrichment analysis of core and dispensable SVs.

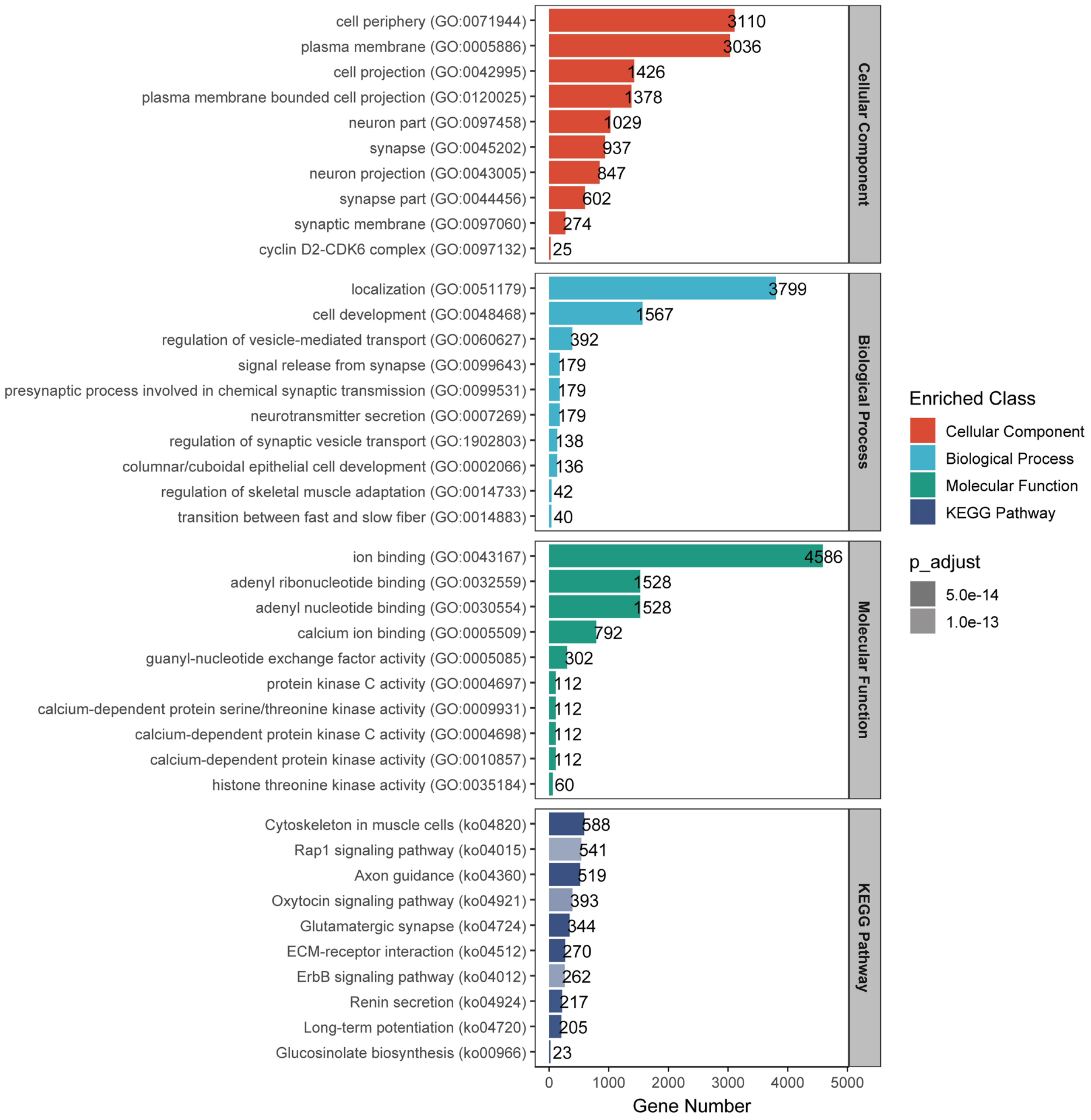

Figure S5. Enrichment analysis of novel sequences in 27 accessions compared to T2T assembly. GO and KEGG enrichment analysis of all non-redundantly novel sequences identified from pan genome. All displayed terms correspond to the top 10 ranked by adjusted *p*-value.

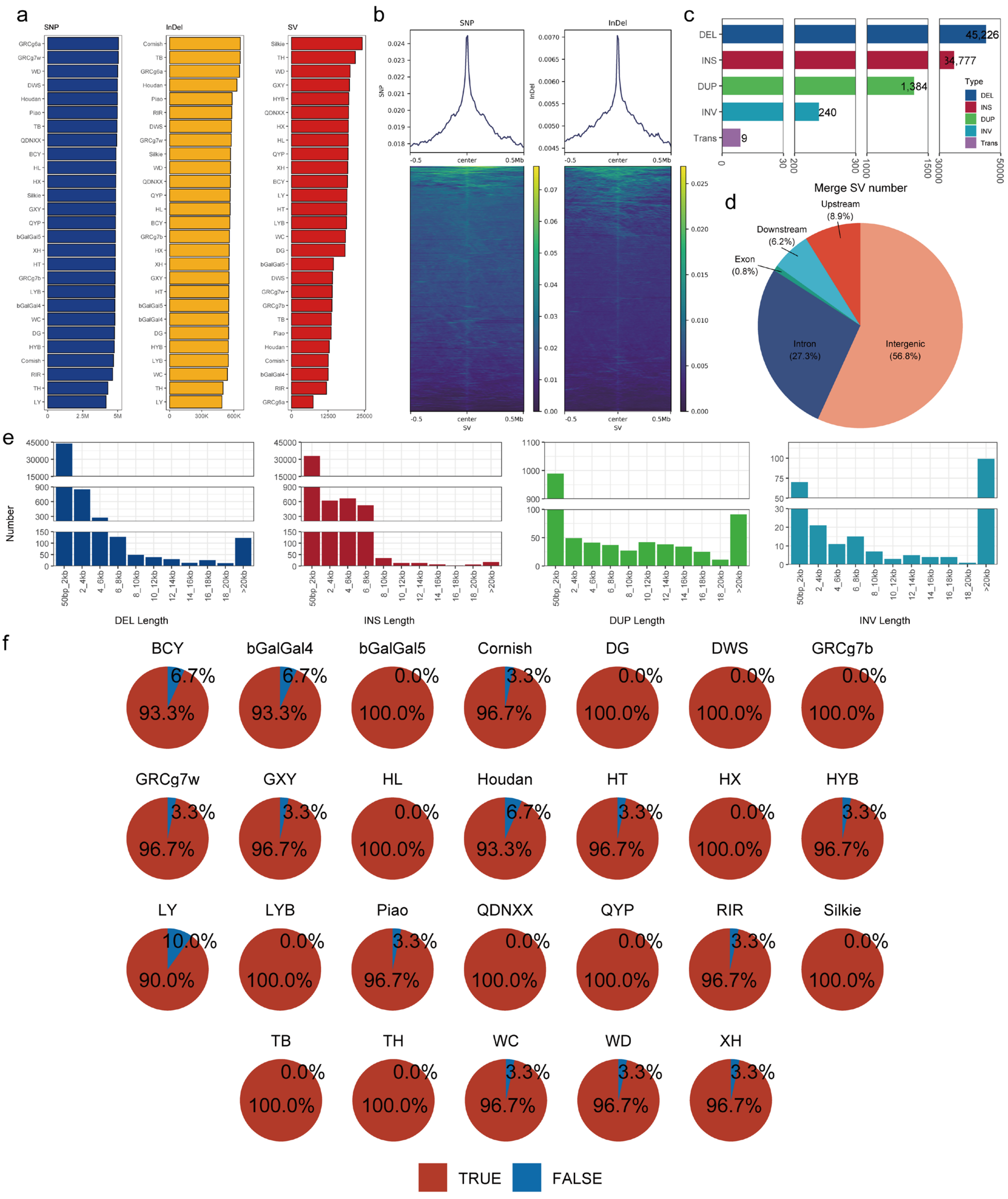

Figure S6. Characteristics of SVs in chicken. a) Number of genetic variations (SNPs, InDels, SVs) detected in each assembly. b) Enrichment patterns of SNPs and InDels within the upstream and downstream 500 kb of SV. c) Number of non-redundant SVs after merging. d) ANNOVAR annotation of non-redundant SVs. e) Length distribution of non-redundant DEL, INS, DUP, and INV. f) IGV validation of randomly selected SVs using long-read sequencing data, with GRCg6a excluded from analysis due to insufficient long-read sequencing depth.

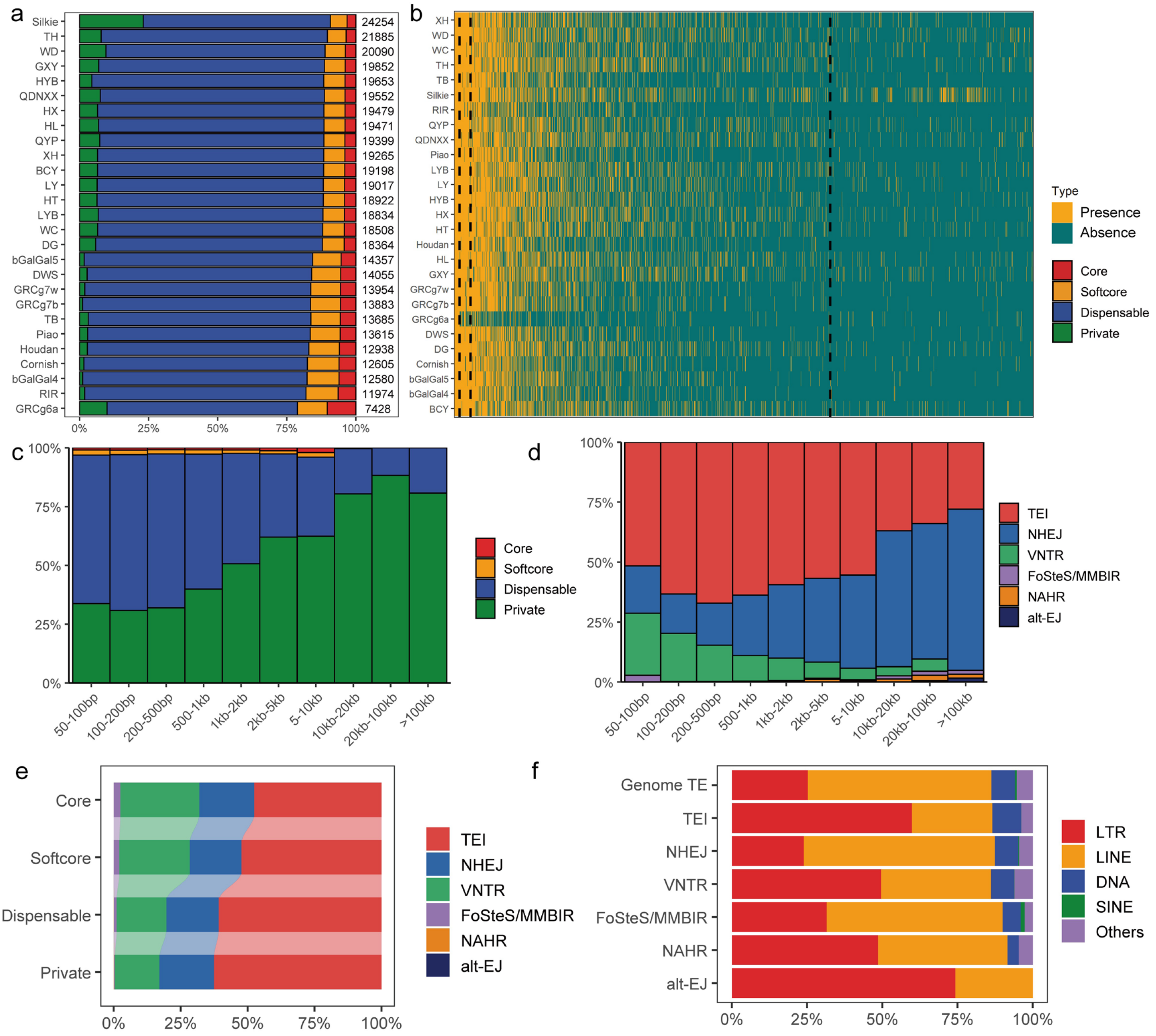

Figure S7. Characteristics of pan-SVs in chicken. a) Proportion of pan-SVs in each chicken assembly. The number on the right represents SV number detected for each assembly. b) Presence-absence information of SV in chicken. c) Proportion of pan-type in SVs with different SV sizes. d) Proportion of formation mechanisms of SVs with different sizes. e) Proportion of different mechanisms forming pan-SVs. f) A comparison of proportion of TE subtypes across different categories of TEs. Abbreviation: TEI: transposable element insertion; NHEJ: nonhomologous end joining; VNTR: variable number of tandem repeats; FoSTeS/MMBIR: fork stalling and template switching/microhomology-mediated break induced repair; NAHR: nonallelic homologous recombination; alt-EJ: alternative end joining.

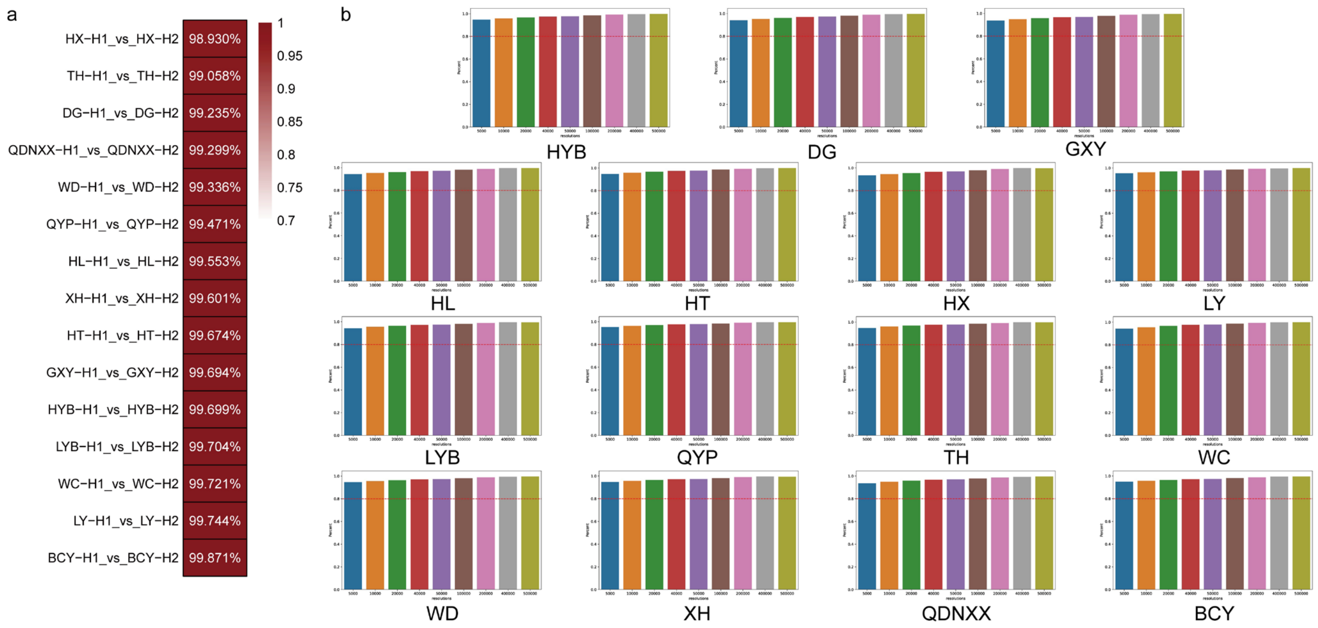

Figure S8. Correlation among Hi-C libraries and available resolutions. a) Correlation matrix of Hi-C libraries for 15 accessions in HiCRep. b) Matric resolution analysis of Hi-C library for each accession (80 % of loci have at least 1,000 interactions).

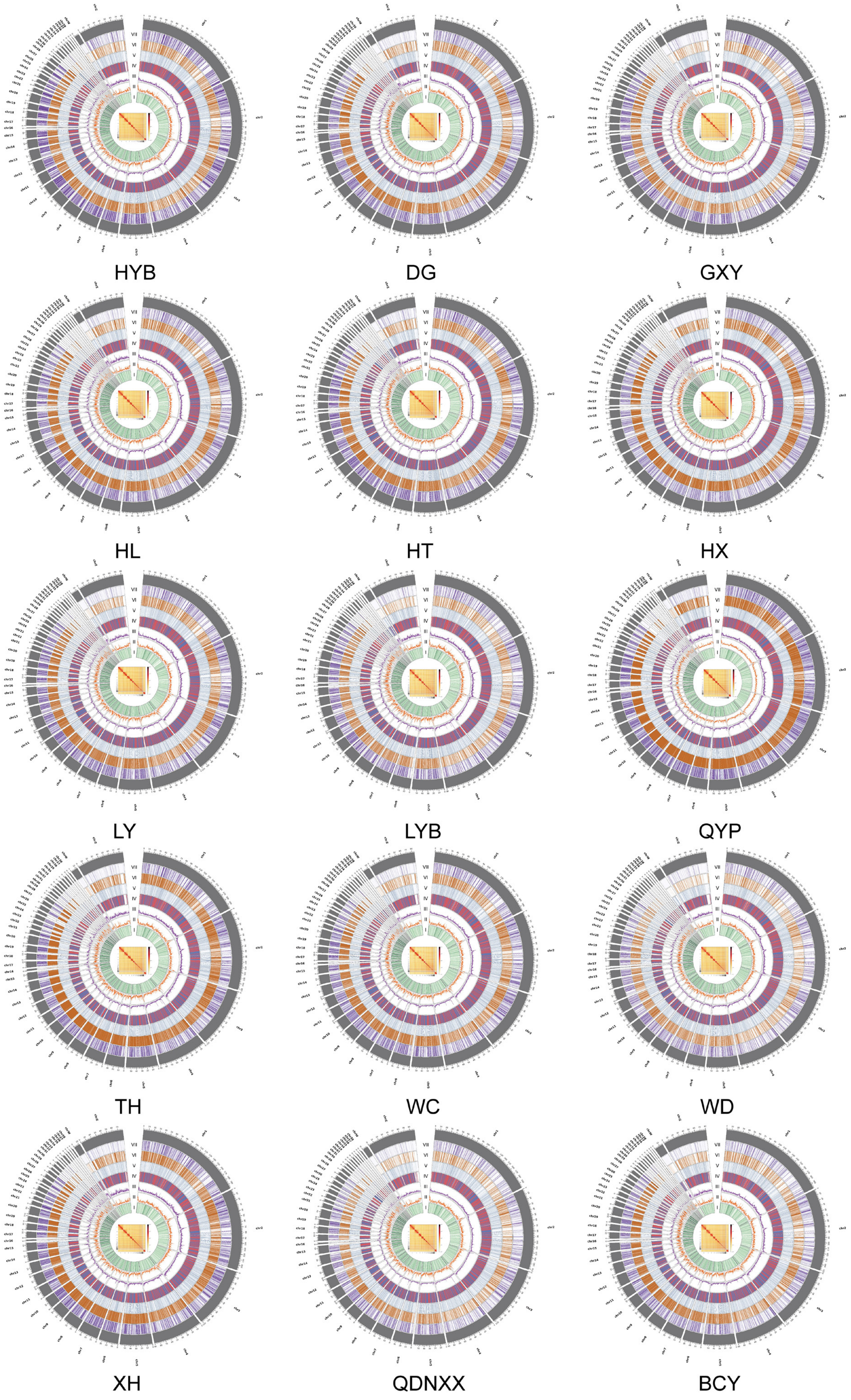
HYB
DG
GXY
HL
HT
HX
LY
LYB
QYP
TH
WC
WD
XH
QDNXX
BCY

Figure S9. Circos plots showing the landscape of 3D chromatin architectures for 15 accessions based on their corresponding genomes. The central part of the circos plots displays the heatmaps of whole-genome Hi-C interaction for each accession in a resolution of 400 kb. Tracks from inside to outside were as follows: I: Gene density; II: GC content; III: Repetitive sequence density; IV: Distribution of A/B compartments (Red: A, Blue: B); V: Distribution of TAD boundaries; VI: Distribution of loop anchors; VII: Distribution of LT-CREs.

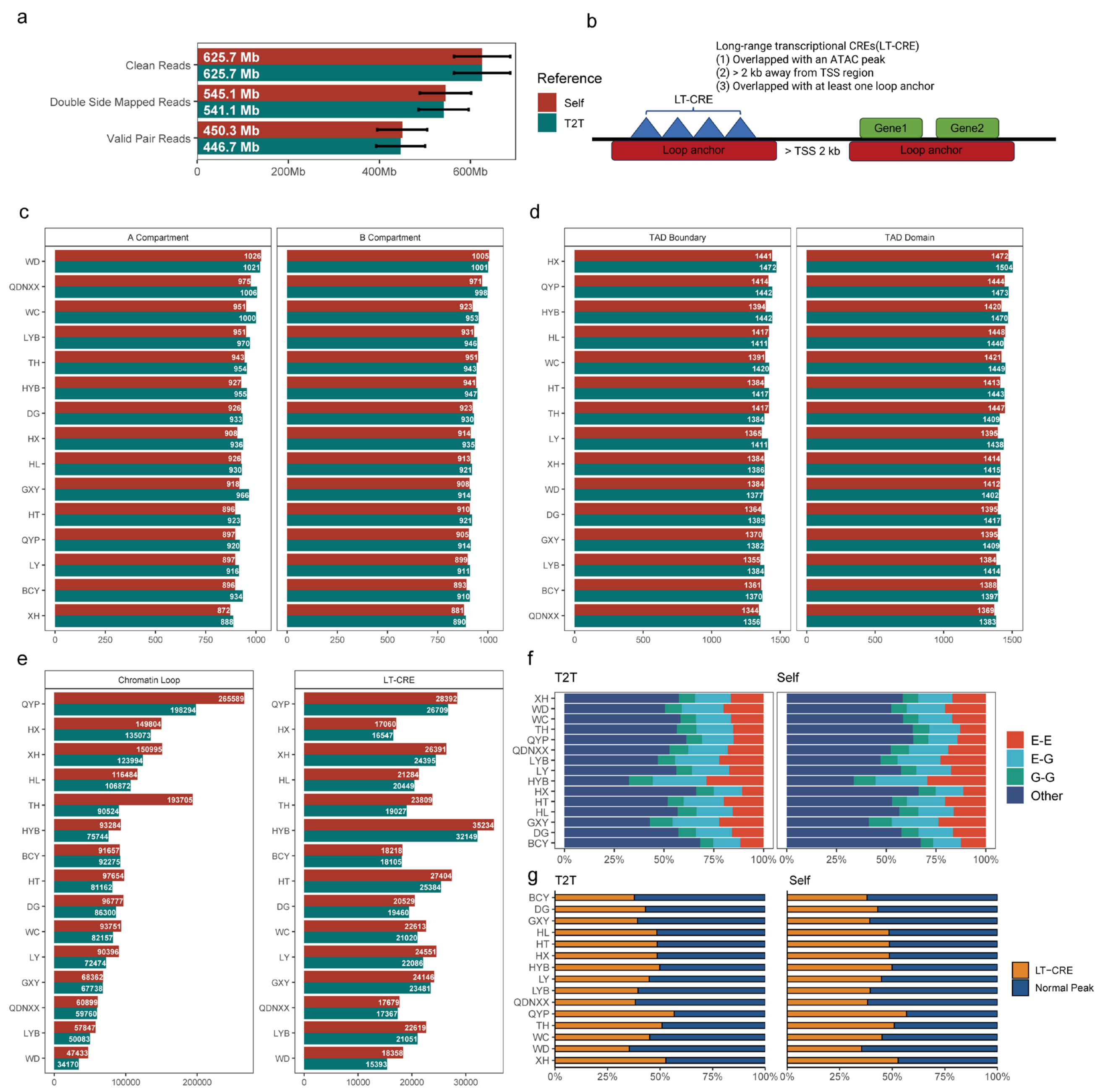

Figure S10. Identification of 3D chromatin architectures based on single T2T genome and their corresponding genome. a) Clean reads, double-sided mapped reads, and valid pair reads statistics of the Hi-C library based on T2T genome and their corresponding genome. b) Schematic diagram of the method for identifying LT-CREs. c) The number of A and B compartments identified based on T2T genome and their corresponding genome d) The number of TAD boundaries and domains identified based on T2T genome and their corresponding genome e) The number of chromatin loops and LT-CREs identified based on T2T genome and their corresponding genome f) Proportion of LT-CRE-LT-CRE (E-E), LT-CRE-Gene (E-G) and Gene-Gene (G-G) loops for each accession based on based on T2T genome and their corresponding genome g) Proportion of LT-CREs in all ATAC peaks identified for each accession based on T2T genome and their corresponding genome.

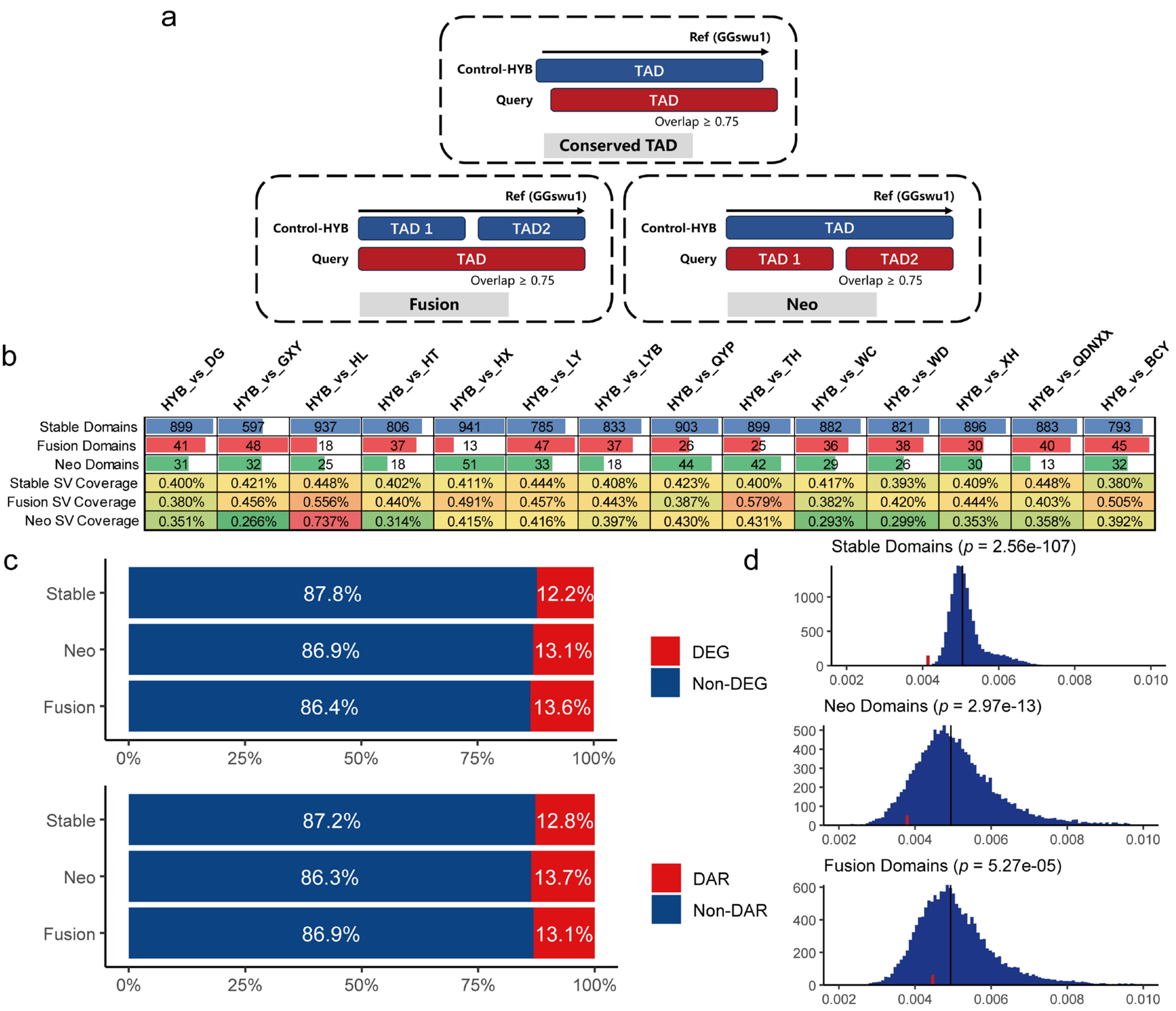

Figure S11. Characteristics of TAD reorganization events in chicken. a) Schematic diagram of the method for identifying TAD domain events. b) Number of TAD domains events and the SV coverage in each reorganization type for each accession. c) Proportion of differentially expressed genes (DEG) and differentially accessible regions (DAG) in TAD domains with different reorganization types. d) Observed (red bar) and expected distribution (blue histograms) of SV coverage in three-type reorganization events. Significant difference was calculated using a two-sided Wilcoxon rank-sum test.

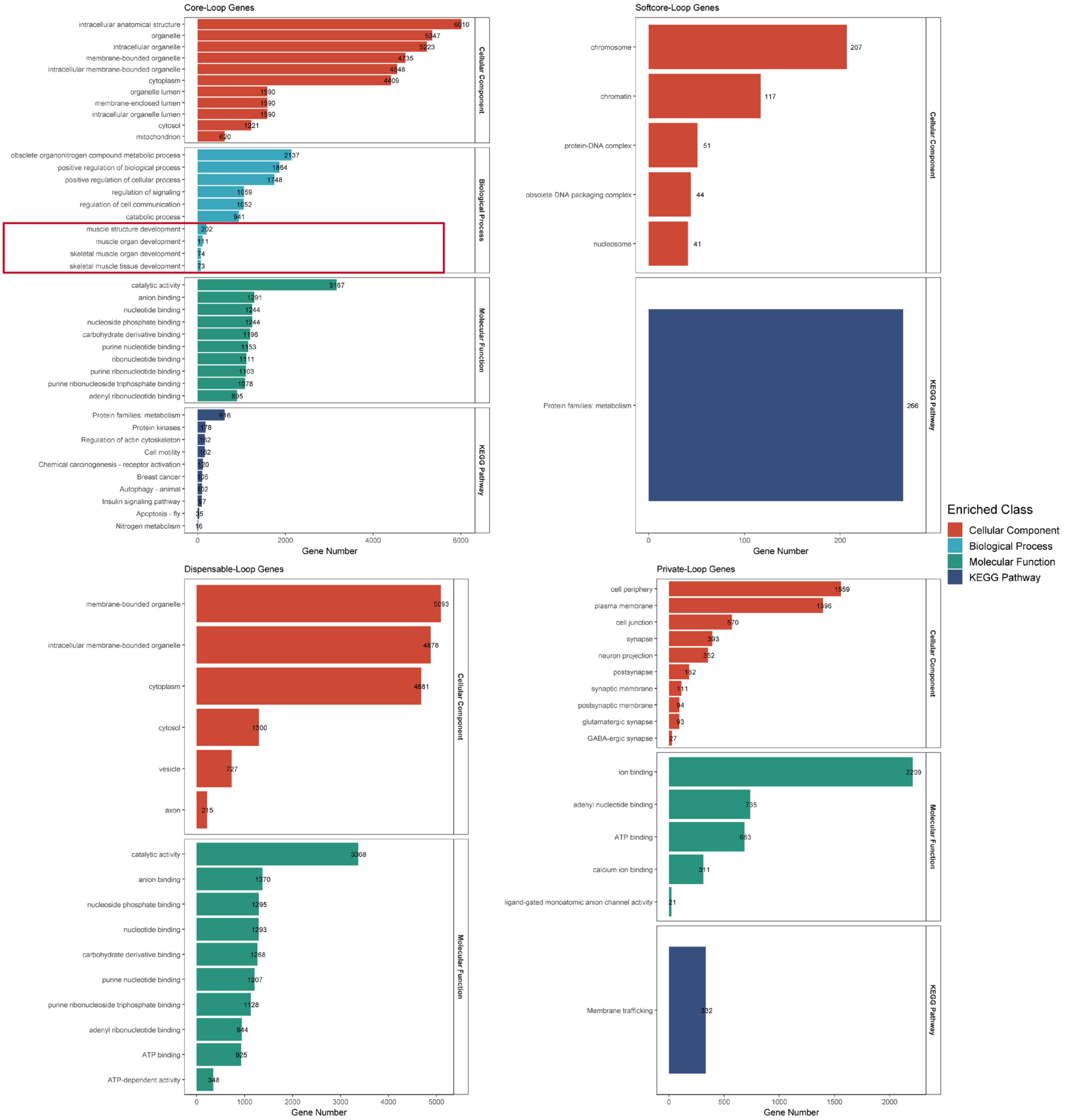

Figure S12. Significantly functional enrichment of genes in different pan-loops. All displayed enrichment terms were filtered with adjusted *p* value < 0.05. Terms associated with muscle growth and development are highlighted in red box.

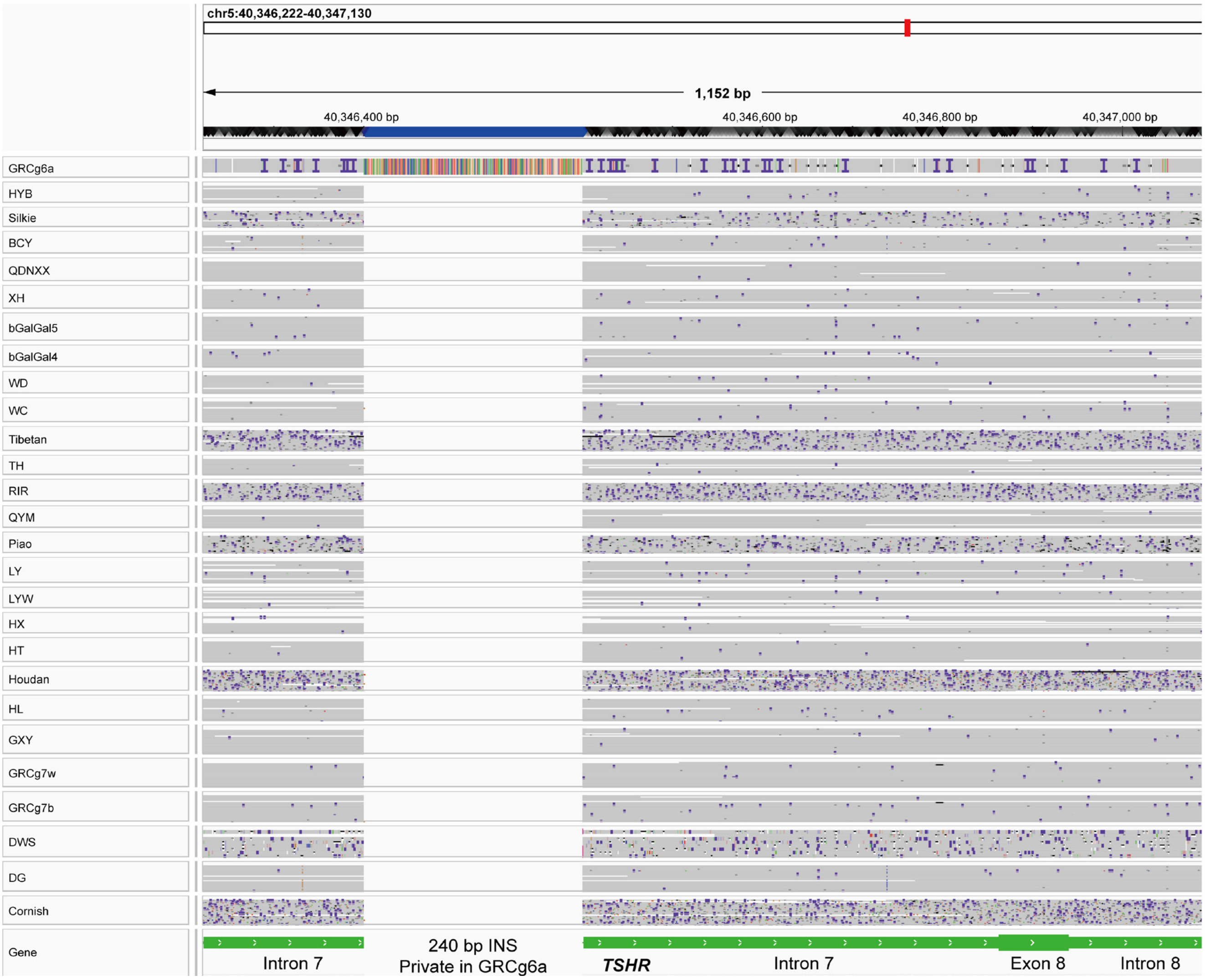

Figure S13. Integrative genomics viewer of 240-bp INS of *TSHR* genes in Pan-SV database. The first 27 tracks represent alignment results of 27 assemblies against the T2T assembly. The final track is the gene structure of *TSHR*.

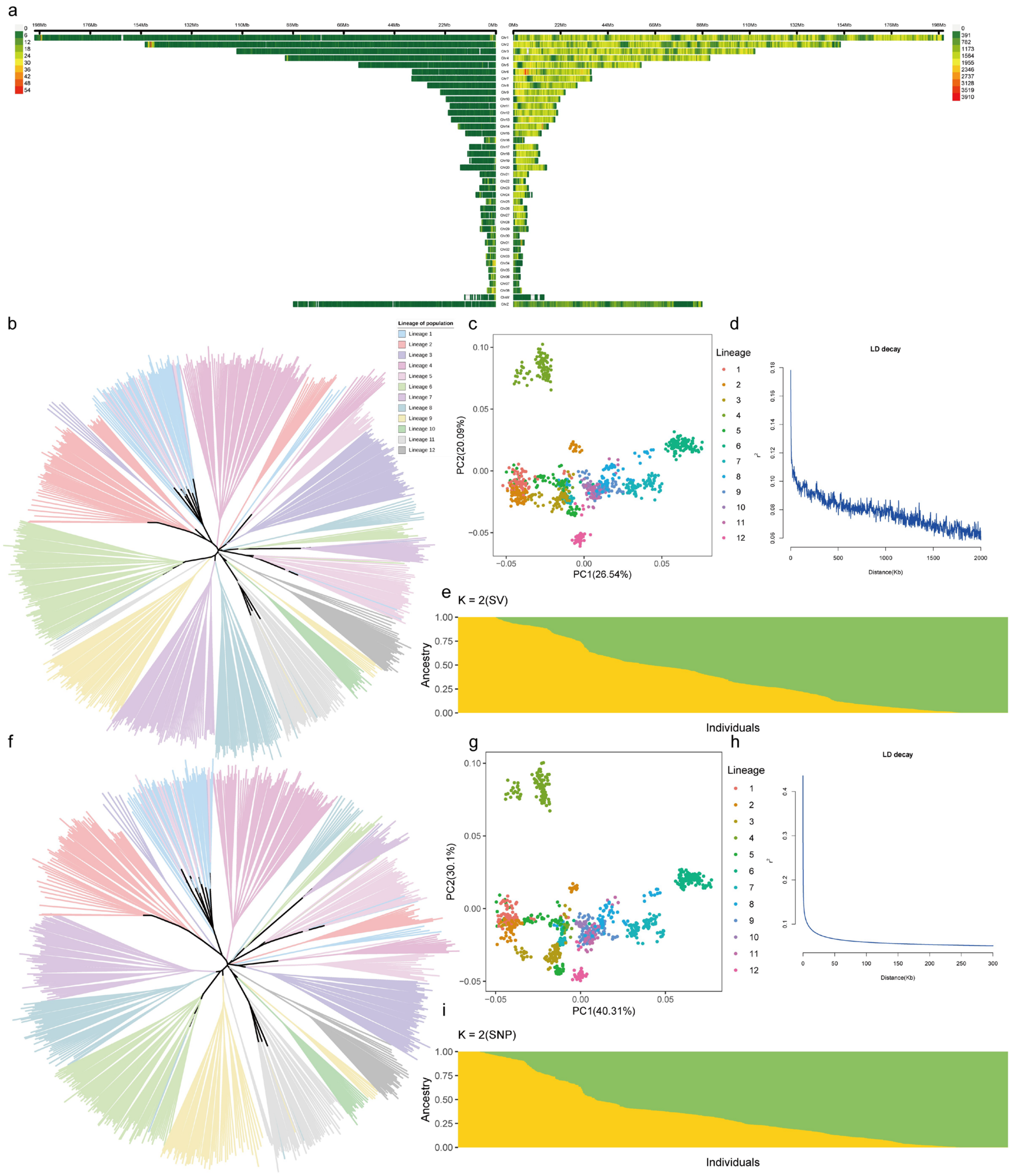

Figure S14. Population genomic analysis of F2 population based on SVs and SNPs. a) Genome distribution of SVs (left) and SNPs (right) of F2 population after filtering (MAF > 0.05). The analysis of b) phylogenetic tree, c) PCA, d) LD decay and e) genetic structure clustering of F2 population based on SVs. The analysis of f) phylogenetic tree, g) PCA, h) LD decay and i) genetic structure clustering of F2 population based on SNPs.

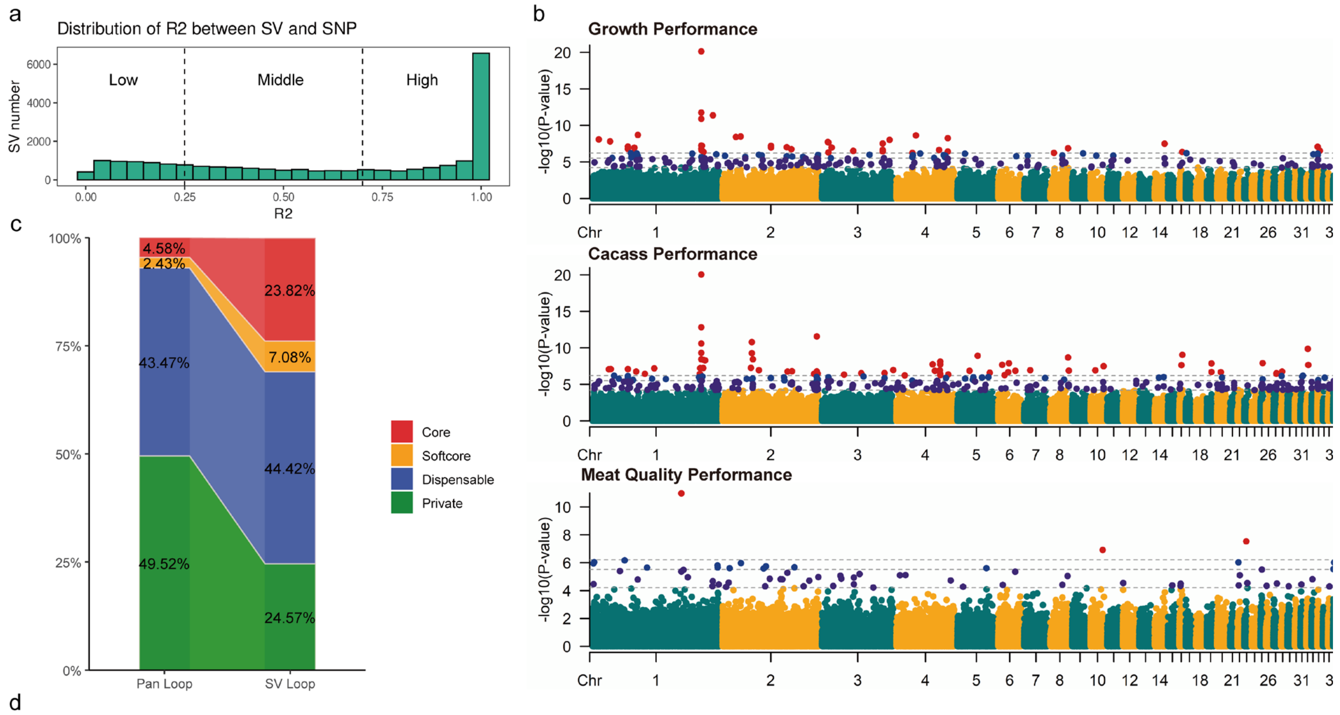

d

Trait type	Traits	Full Name	SV Number	Gene Number	Loop-Gene Number
Growth performance	ADG	Average daily gain(4-8w)	21	10	50
	BW1	Body weight at 1 days of age	24	17	82
	BW7	Body weight at 7 days of age	16	11	121
	BW14	Body weight at 14 days of age	13	3	42
	BW21	Body weight at 21 days of age	13	4	52
	BW28	Body weight at 28 days of age	21	9	79
	BW35	Body weight at 35 days of age	20	13	22
	BW42	Body weight at 42 days of age	13	9	32
	BW49	Body weight at 49 days of age	18	8	44
	BW56	Body weight at 56 days of age	19	14	70
	BW63	Body weight at 63 days of age	18	12	43
	BW70	Body weight at 70 days of age	22	13	51
	BW77	Body weight at 77 days of age	13	8	25
	BW84	Body weight at 84 days of age	18	6	73
	HW	Head width	7	4	19
	SL	Shank length	1	0	0
Meat quality performance	BMcolor	Breast muscle color(L*value)	4	3	7
	LMcolor	Leg muscle color(L*value)	6	5	58
	BMcon	Breast muscle conductivity	15	11	51
	LMcon	Leg muscle conductivity	7	2	35
	BMpH	pH value of breast muscle	3	2	17
	LMpH	pH value of leg muscle	12	9	32
	BMSF	Shear force of breast muscle	20	15	155
	LMSF	Shear force of leg muscle	12	6	33
Carcass performance	LW	Live weight before slaughter	13	8	81
	DW	Dressed weight	15	4	92
	AFW	Abdominal fat weight	19	13	71
	BMW	Breast muscle weight	18	8	70
	LMW	Leg muscle weight	17	11	66
	EW	Eviscerated weight	19	9	108
	HEW	Half-eviscerated weight with giblet	18	7	182
	HNW	Head and neck weight	19	14	87
	WW	Wing weight	16	13	180
	SFW	Shank and foot weight	19	12	86
	SFT	Subcutaneous fat thickness	34	28	101
	HLGPW	Heart liver gizzard and proventriculus weight	13	7	84
	IFW	Intermuscular fat width	12	6	72
	LBMR	Leg to breast muscle ratio	20	11	60
	SIL	Small intestine length	11	8	64
	PD	Dressed percentage	2	1	8
	PAFY	Percentage of abdominal fat yield	14	8	102
	PBMY	Percentage of breast muscle yield	18	13	161
	PLMY	Percentage of leg muscle yield	13	4	46
	PEY	Percentage of eviscerated yield	4	4	6
	PHEY	Percentage of half-eviscerated yield with giblet	1	1	6
	PHNW	Percentage of head and neck weight	12	10	35
	PWY	Percentage of wing yield	3	1	9
	PSFW	Percentage of shank and foot weight	17	11	70
	PMY	Percentage of meat yield	2	2	4

Figure S15. Summary of SV-GWAS in 49 production traits of F2 population. a) Linkage analysis

between SVs from the graph-based pan-genome and their nearby (± 500 kb) SNPs. The vertical dashed lines on the left and right represent R2 = 0.25 and 0.7, respectively. b) Manhattan plot of SV-GWAS in growth, carcass, and meat-quality performance traits. Three horizontal dashed lines represent significance thresholds at 0.01/N, 0.05/N, and 1/N, respectively (N referred to number of SVs remaining after LD pruning). c) Proportion of pan-type of loops between trait-associated SVs regions and whole genome. d) Statistics of the number of significantly associated SVs, SV-genes, and SV-loop-genes in SV GWAS for each trait.

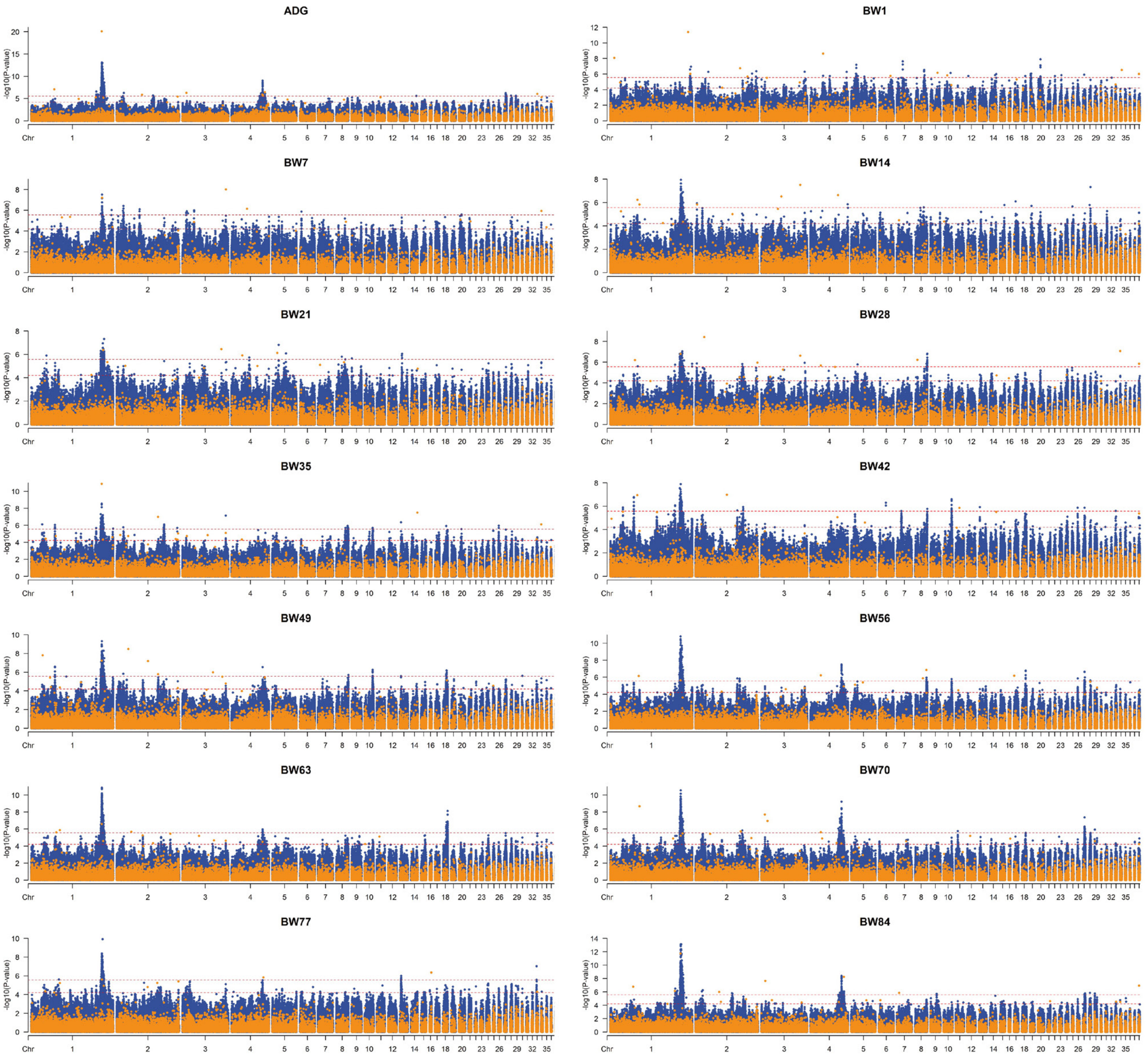

Figure S16. Manhattan plots of SNP and SV-GWAS for 12 growth performance traits. The blue and orange points represented SNP and SV markers. Upper and lower horizontal dashed lines are the significance threshold of SNPs and SVs (p = 1/N, N referred to number of markers remaining after LD pruning), respectively. Abbreviation: ADG: Average daily gain (4-8 weeks); BW1: Body weight at 1 days of age; BW7: Body weight at 7 days of age; BW14: Body weight at 14 days of age; BW21: Body weight at 21 days of age; BW28: Body weight at 28 days of age; BW35: Body weight at 35 days of age; BW42: Body weight at 42 days of age; BW49: Body weight at 49 days of age; BW56: Body weight at 56 days of age; BW63: Body weight at 63 days of age; BW70: Body weight at 70 days of age; BW77: Body weight at 77 days of age; BW84: Body weight at 84 days of age; HW: Head width; SL: Shank length.

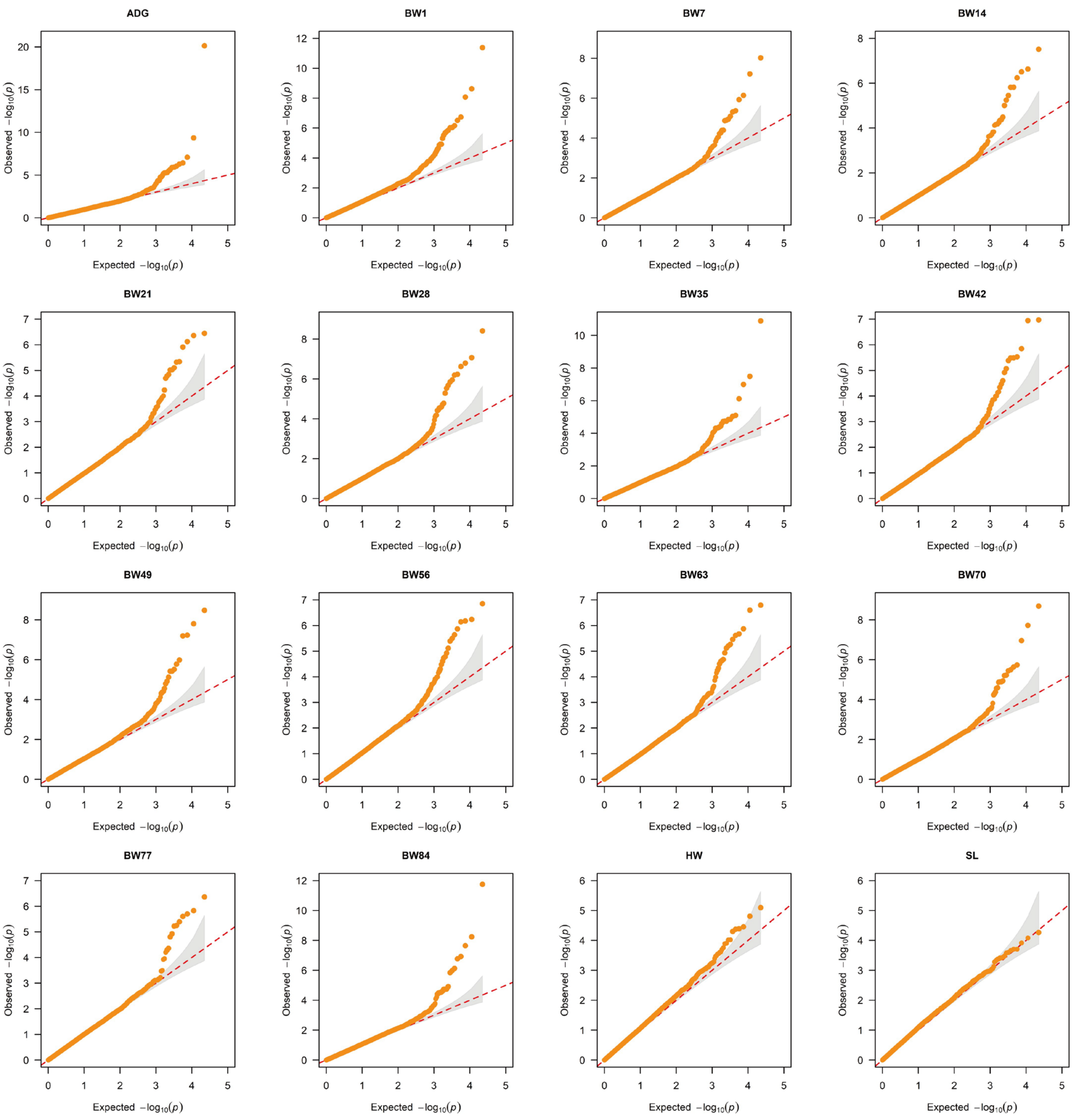

SV-GWAS

Figure S17. QQ plots of SV-GWAS for 12 growth performance traits. The orange points represented SV markers. The significance thresholds of SV-GWAS were self-defined as p = 1/N, where N referred to number of markers remaining after LD pruning. Full names of abbreviations were listed in Figure S16.

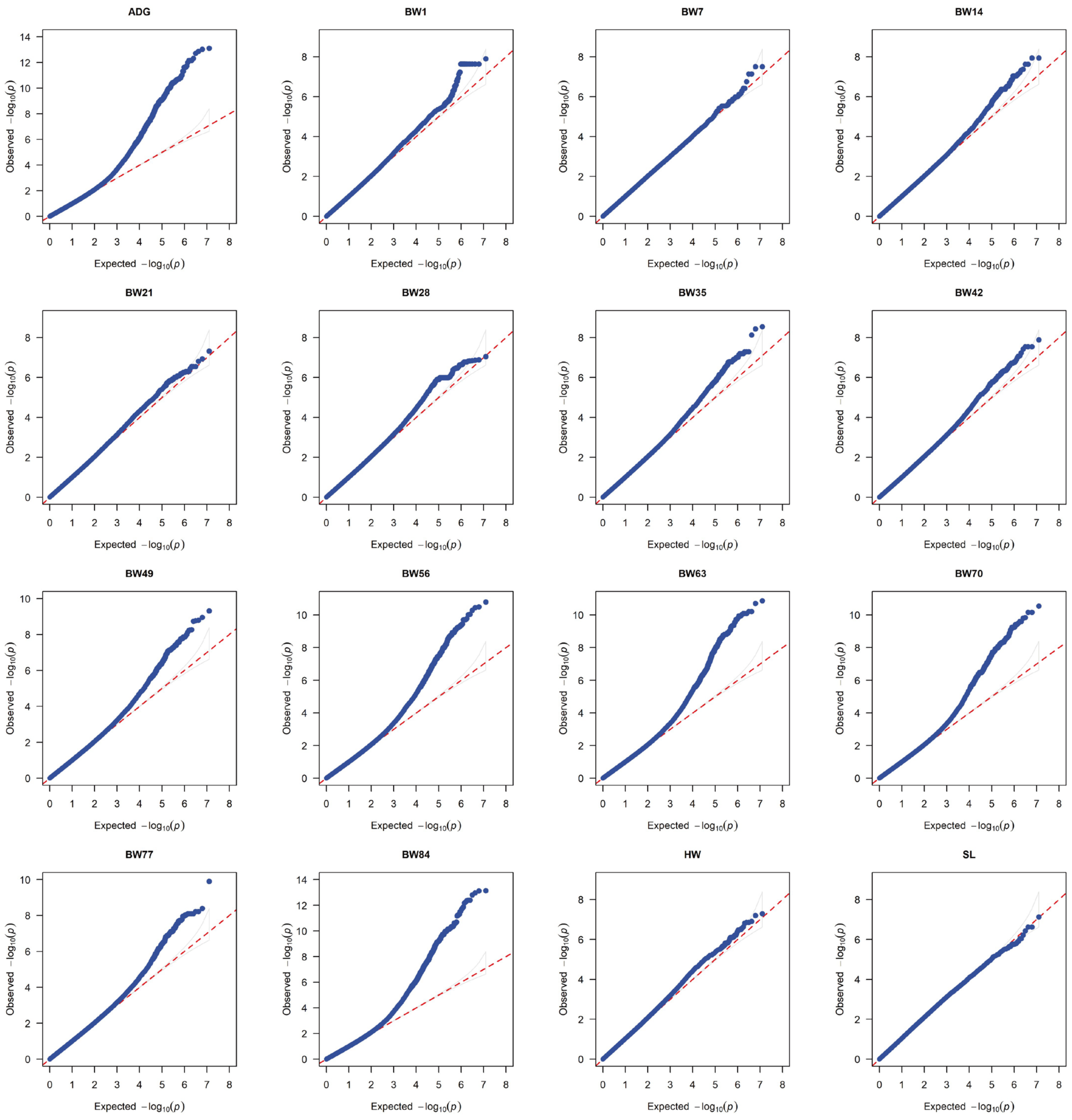

SNP-GWAS

Figure S18. QQ plots of SNP-GWAS for 12 growth performance traits. The blue points represented SNP markers. The significance thresholds of SNP-GWAS were self-defined as p = 1/N, where N referred to number of markers remaining after LD pruning. Full names of abbreviations were listed in Figure S16.

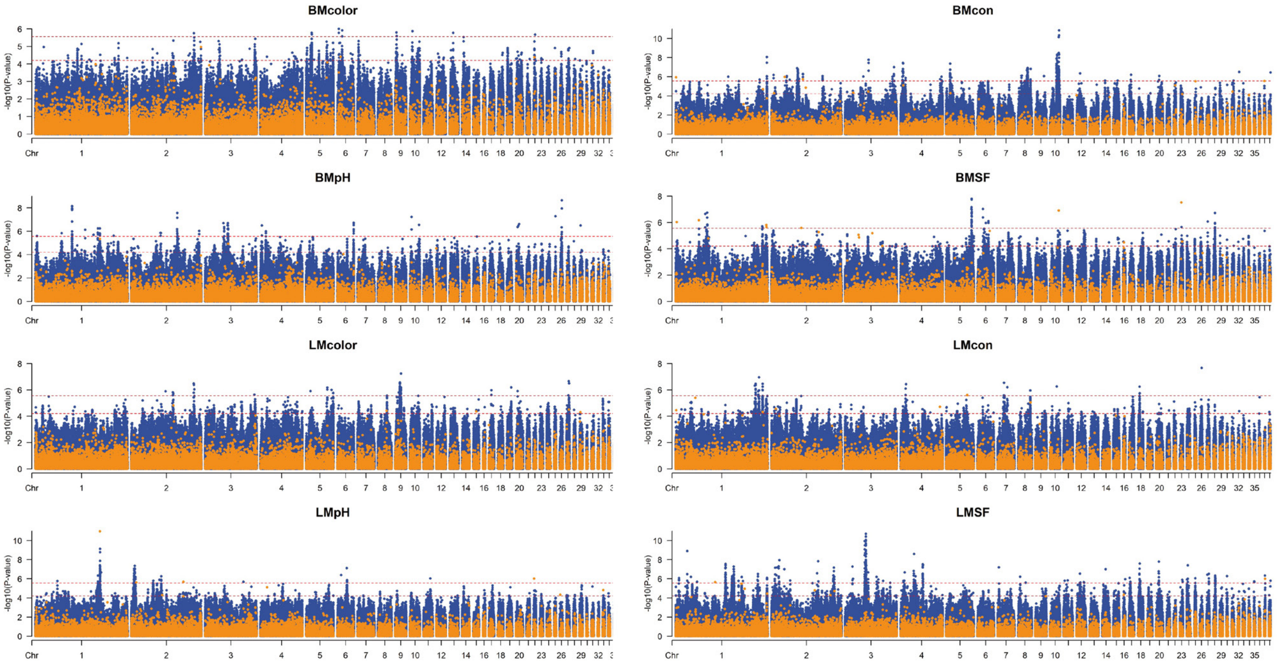

Figure S19. Manhattan plots of SNP and SV-GWAS for 8 meat-quality performance traits. The blue and orange points represented SNP and SV markers. Upper and lower horizontal dashed lines are the significance threshold of SNPs and SVs (*p* = 1/N, N referred to number of markers remaining after LD pruning), respectively. Abbreviation: BMcolor: Breast muscle color (L*value); BMcon: Breast muscle conductivity; LMcolor: Leg muscle color (L*value); LMcon: Leg muscle conductivity; BMpH: pH value of breast muscle; LMpH: pH value of leg muscle; BMSF: Shear force of breast muscle; LMSF: Shear force of leg muscle.

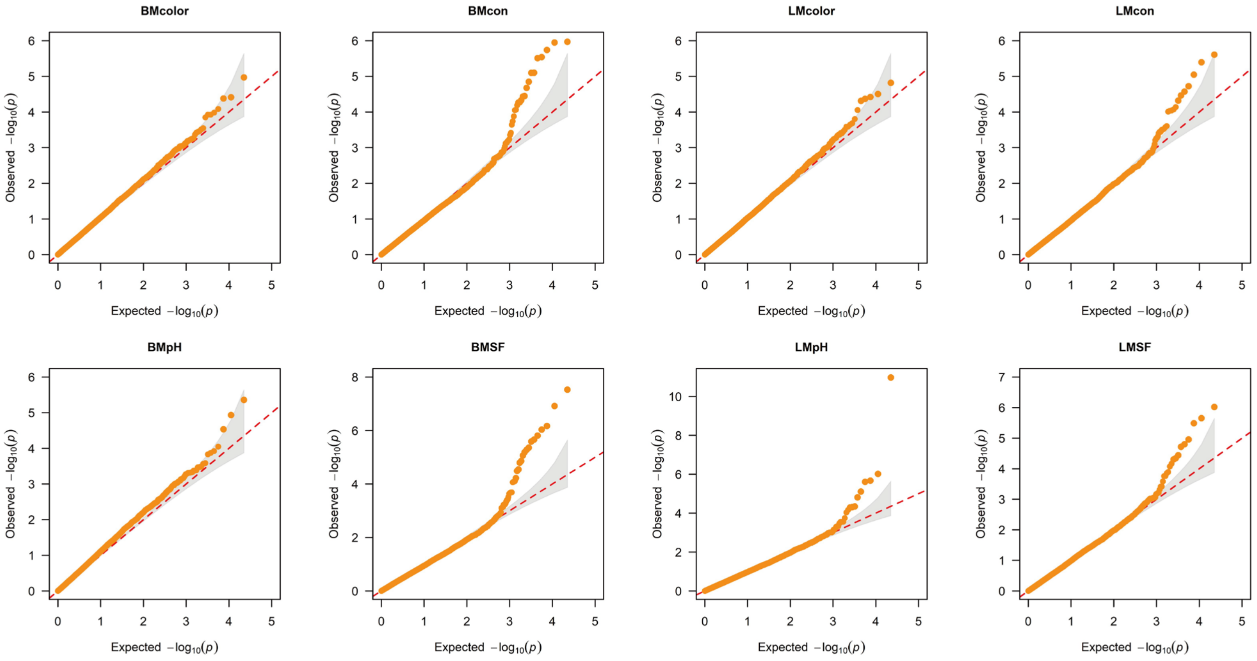

SV-GWAS

Figure S20. QQ plots of SV-GWAS for 8 meat-quality performance traits. The orange points represented SV markers. The significance thresholds of SV-GWAS were self-defined as $p = 1/N$, where N referred to number of markers remaining after LD pruning. Full names of abbreviations were listed in Figure S19.

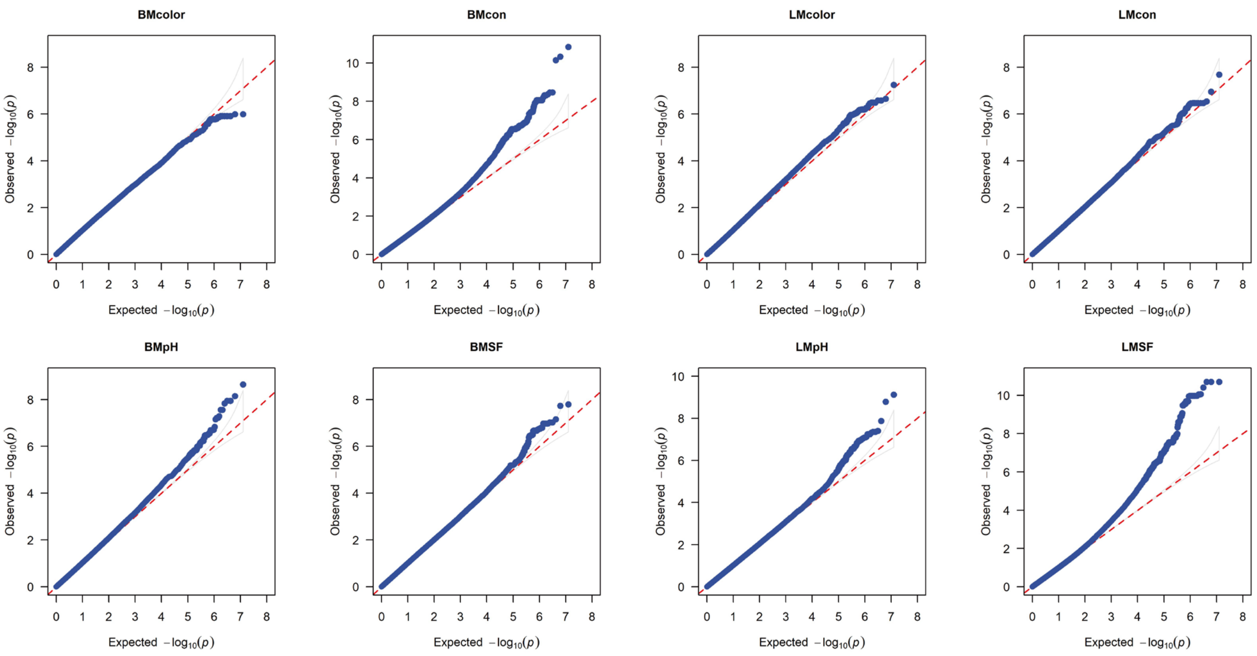

SNP-GWAS

Figure S21. QQ plots of SNP-GWAS for 8 meat-quality performance traits. The blue points represented SNP markers. The significance thresholds of SNP-GWAS were self-defined as $p = 1/N$, where N referred to number of markers remaining after LD pruning. Full names of abbreviations were listed in Figure S19.

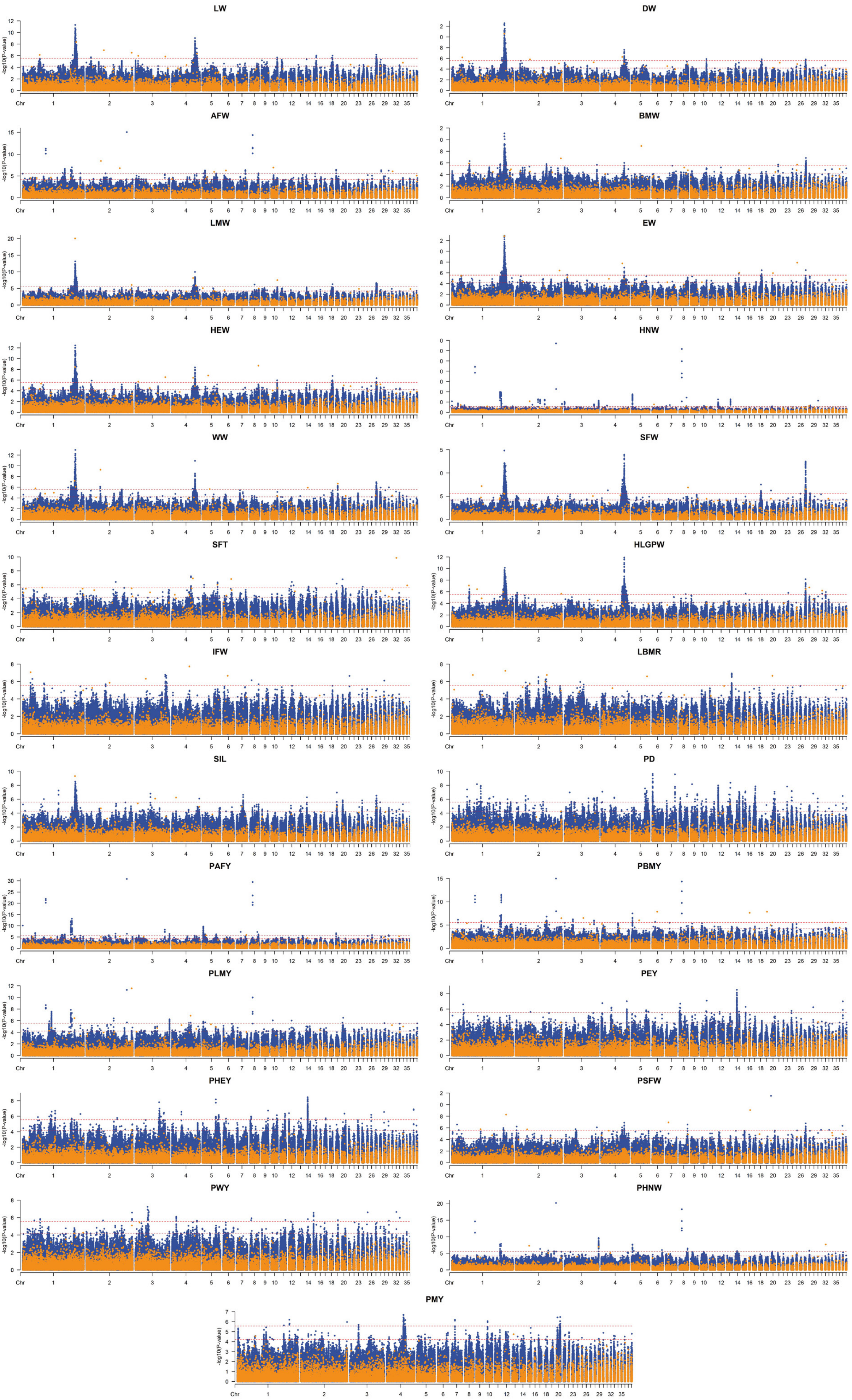
LW
DW
AFW
BMW
LMW
EW
HEW
HNW
WW
SFW
SFT
HLGPW
IFW
LBMR
SIL
PD
PAFY
PBMY
PLMY
PEY
PHEY
PSFW
PWY
PHNW
PMY
-log10(P-value)
Chr
1 2 3 4 5 6 7 8 9 10 12 14 16 18 20 23 26 29 32 35

Figure S22. Manhattan plots of SNP and SV-GWAS for 25 carcass performance traits. The blue and orange points represented SNP and SV markers. Upper and lower horizontal dashed lines are the significance threshold of SNPs and SVs (p = 1/N, N referred to number of markers remaining after LD pruning), respectively. Abbreviation: LW: Live weight before slaughter; DW: Dressed weight; AFW: Abdominal fat weight; BMW: Breast muscle weight; LMW: Leg muscle weight; EW: Eviscerated weight; HEW: Half-eviscerated weight with giblet; HNW: Head and neck weight; WW: Wing weight; SFW: Shank and foot weight; SFT: Subcutaneous fat thickness; HLGPW: Heart liver gizzard and proventriculus weight; IFW: Intermuscular fat width; LBMR: Leg to breast muscle ratio; SIL: Small intestine length; PD: Dressed percentage; PAFY: Percentage of abdominal fat yield; PBMY: Percentage of breast muscle yield; PLMY: Percentage of leg muscle yield; PEY: Percentage of eviscerated yield; PHEY: Percentage of half-eviscerated yield with giblet; PSFW: Percentage of shank and foot weight; PWY: Percentage of wing yield; PHNW: Percentage of head and neck weight; PMY: Percentage of meat yield.

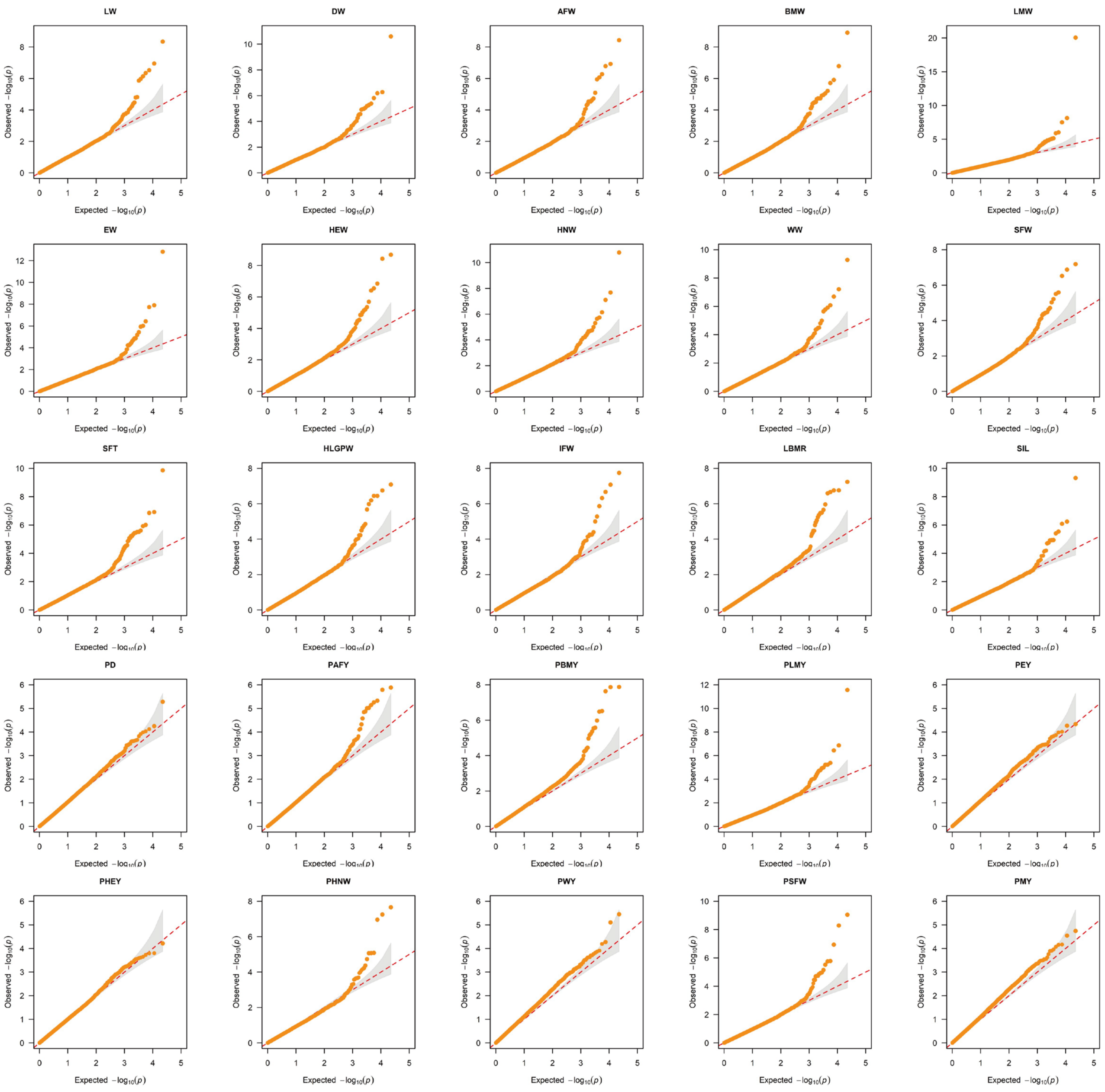

SV-GWAS

Figure S23. QQ plots of SV-GWAS for 25 carcass performance traits. The orange points represented SV markers. The significance thresholds of SV-GWAS were self-defined as p = 1/N, where N referred to number of markers remaining after LD pruning. Full names of abbreviations were listed in Figure S22.

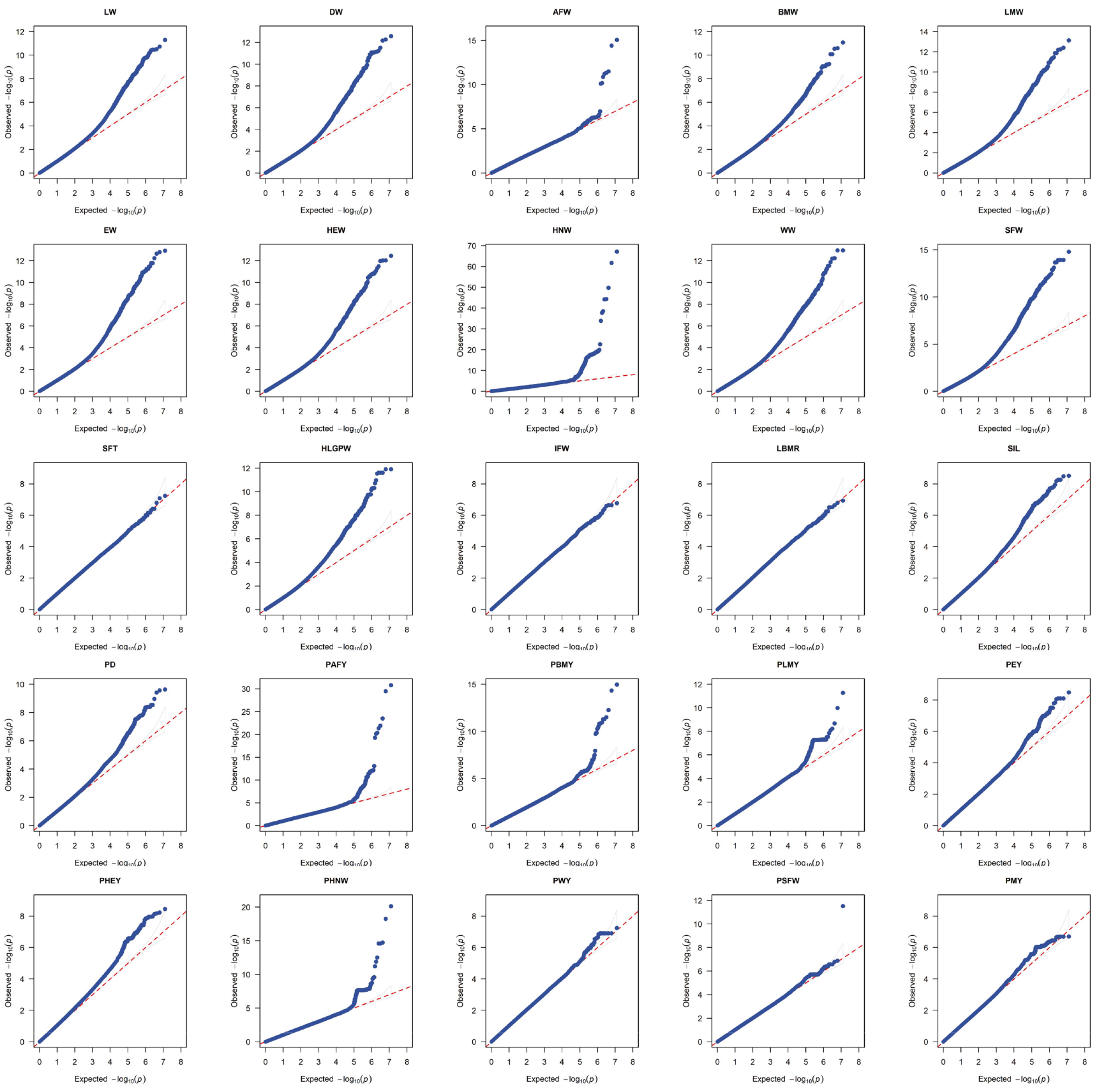

SNP-GWAS

Figure S24. QQ plots of SNP-GWAS for 25 carcass performance traits. The blue points represented SNP markers. The significance thresholds of SNP-GWAS were self-defined as p = 1/N, where N referred to number of markers remaining after LD pruning. Full names of abbreviations were listed in Figure S22.

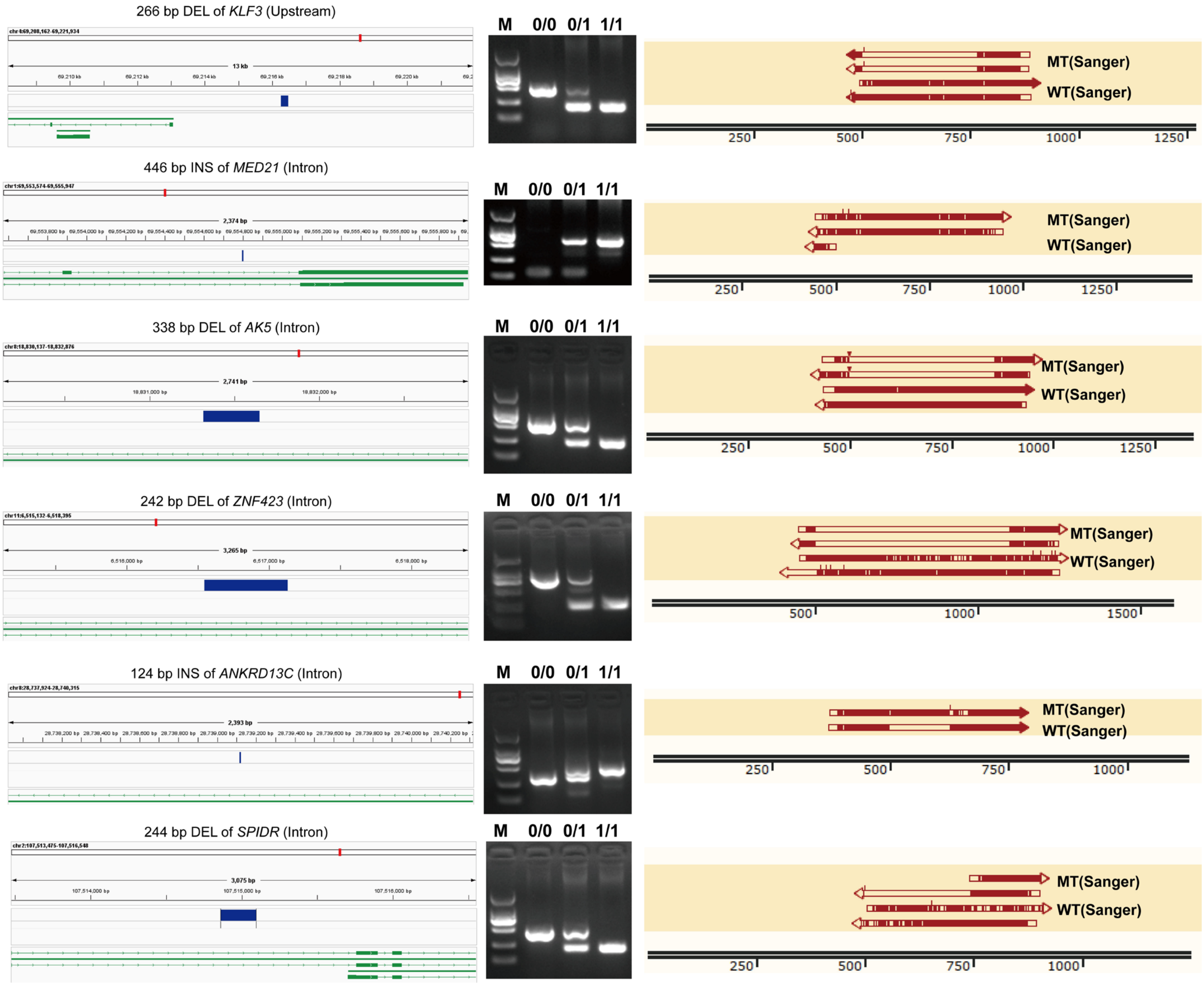

Figure S25. PCR and Sanger sequencing validation of partial candidate structural variations. *KLF3*: Kruppel-like factor 3; *MED21*: mediator complex subunit 21; *AK5*: adenylate kinase 5; *ZNF423*: zinc finger protein 423; *ANKRD13C*: ankyrin repeat domain 13C; *SPIDR*: scaffolding protein Scaffolding protein involved in DNA repair. The DNA marker bands, from top to bottom, are 2000 bp, 1000 bp, 750 bp, 500 bp, 250 bp, and 100 bp.

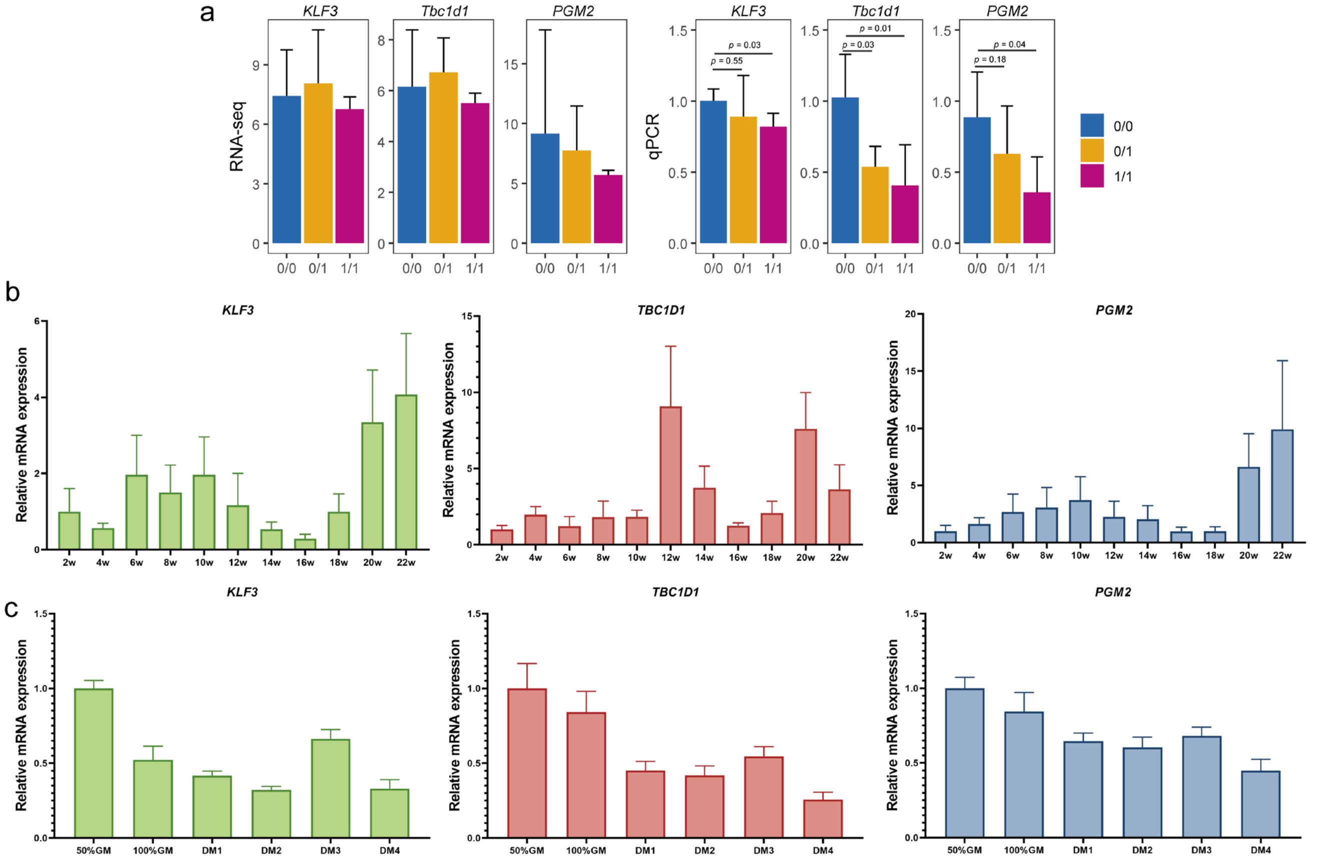

Figure S26. *KLF3*, *Tbc1d1* and *PGM2* expression profiles across different SV genotypes and stages of broiler growth and myoblast development. a) Comparison of gene expression levels of *KLF3*, *Tbc1d1* and *PGM2* among different SV genotypes (Left: RNA-seq results; Right: qPCR validation). b) Gene expression profiles of *KLF3*, *Tbc1d1* and *PGM2* in skeletal muscle of broiler at different growth stages. c) Gene expression profiles of *KLF3*, *Tbc1d1* and *PGM2* during the proliferation and differentiation stages of chicken primary myoblasts. *KLF3*: Kruppel-like factor 3; *Tbc1d1*: TBC1 domain family member 1; *PGM2*: Phosphoglucomutase 2. Significant differences were assessed by two-sided independent t-test.

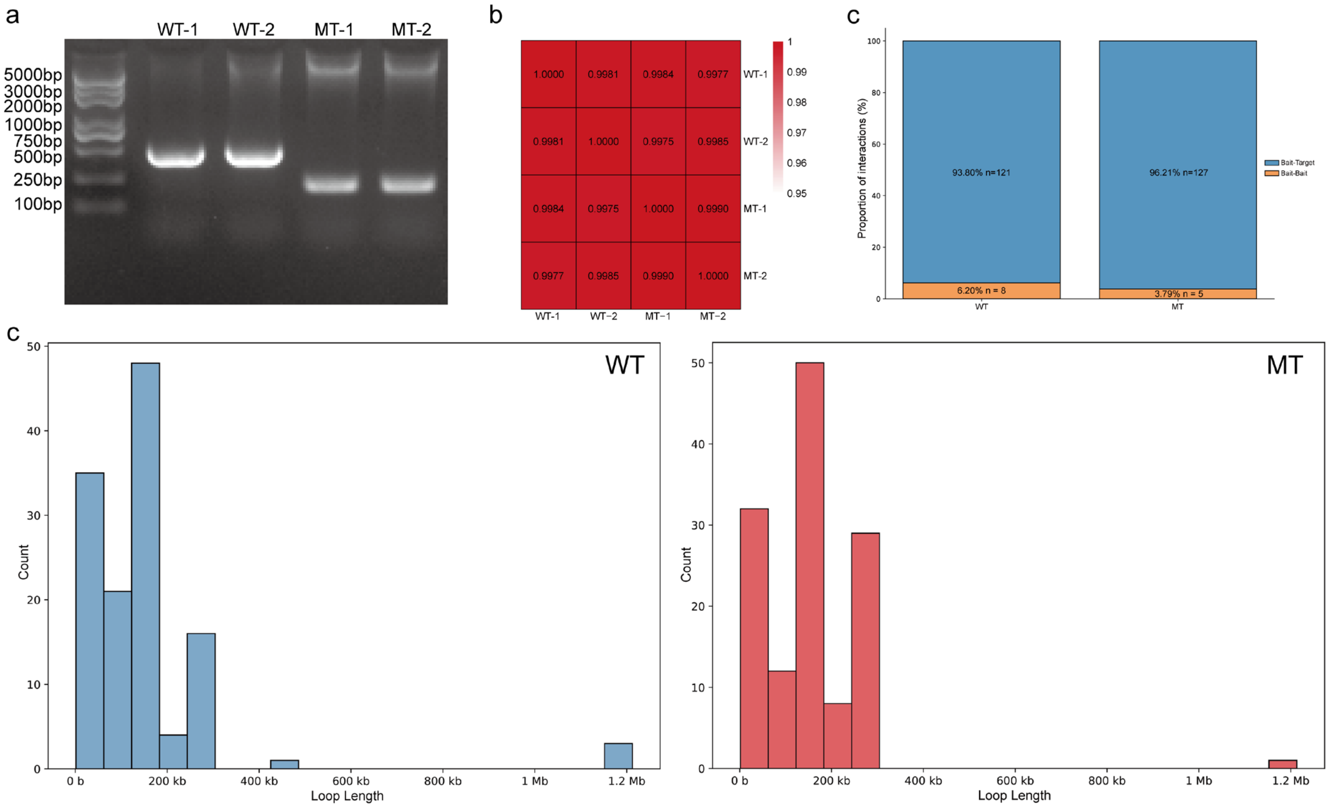

Figure S27. Characterization of Capture Hi-C data in KLF3-SV wild-type and mutant-type samples. a) PCR validation of SV genotypes of wild-type (WT) and mutant-type (MT) samples for Capture Hi-C analysis. b) Correlation matrix among Capture Hi-C libraries for WT and MT samples in HiCRep. c) Proportion of interaction type in Capture Hi-C for WT and MY groups. d) Distribution of span lengths of target chromatin loops in WT and MT groups.
